# Highly Parallel Translation of DNA Sequences into Small Molecules

**DOI:** 10.1371/journal.pone.0028056

**Published:** 2012-03-29

**Authors:** Rebecca M. Weisinger, S. Jarrett Wrenn, Pehr B. Harbury

**Affiliations:** 1 Department of Chemistry, Stanford University, Stanford, California, United States of America; 2 Department of Biochemistry, Stanford University, Stanford, California, United States of America; The Scripps Research Institute, United States of America

## Abstract

A large body of *in vitro* evolution work establishes the utility of biopolymer libraries comprising 10^10^ to 10^15^ distinct molecules for the discovery of nanomolar-affinity ligands to proteins.[1], [2], [3], [4], [5] Small-molecule libraries of comparable complexity will likely provide nanomolar-affinity small-molecule ligands.[6], [7] Unlike biopolymers, small molecules can offer the advantages of cell permeability, low immunogenicity, metabolic stability, rapid diffusion and inexpensive mass production. It is thought that such desirable *in vivo* behavior is correlated with the physical properties of small molecules, specifically a limited number of hydrogen bond donors and acceptors, a defined range of hydrophobicity, and most importantly, molecular weights less than 500 Daltons.[8] Creating a collection of 10^10^ to 10^15^ small molecules that meet these criteria requires the use of hundreds to thousands of diversity elements per step in a combinatorial synthesis of three to five steps. With this goal in mind, we have reported a set of mesofluidic devices that enable DNA-programmed combinatorial chemistry in a highly parallel 384-well plate format. Here, we demonstrate that these devices can translate DNA genes encoding 384 diversity elements per coding position into corresponding small-molecule gene products. This robust and efficient procedure yields small molecule-DNA conjugates suitable for *in vitro* evolution experiments.

## Introduction

Early results with DNA-programmed and DNA-linked chemical libraries suggest that the *in vitro* evolution of small molecules will be a promising approach to compound discovery.[9], [10], [11], [12] These developments build on earlier studies of *in vitro* biopolymer evolution [13], [14], [15], [16], [17], wherein desired binding and catalytic traits were bred into molecular populations by reenacting evolution in a test tube. Analysis of such experiments indicates an empirical relationship between library size and the quality of the resulting molecules [2], [18], [19]: the affinity and catalytic proficiency of selected hits increases with the complexity of the initial library. Biopolymer libraries of 10^10^ to 10^15^ compounds generally yield ligands with nanomolar dissociation constants. The question arises: if library size is central to the success of biopolymer discovery, does the same relationship hold for chemical libraries? Unfortunately, this question is nearly impossible to answer with traditional high-throughput screening (HTS) approaches. HTS libraries typically comprise one million compounds and yield ligands with micromolar dissociation constants. [20] Libraries of billions or trillions of compounds do not exist, and the expense and time necessary to screen collections of that size make such an experiment economically and practically unfeasible. [21]

DNA-linked chemical libraries represent an alternative means to examine whether complex small-molecule collections can be a fruitful source of high-affinity ligands. Tagging small molecules with DNA, as suggested by Lerner and Brenner in 1992, [22] allows complex chemical mixtures to be subjected to selection in bulk for binding to a target. The procedure is inexpensive and rapid. The last five years have witnessed explosive growth in the design of DNA-linked chemical libraries and the selection of molecules from those collections.[9], [10], [11], [12] However, with increasing library complexity, the task of identifying useful ligands (the “needles in the haystack”) has become increasingly difficult. In favorable cases, a bulk selection for binding to a target can enrich a ligand from non-ligands by about 1000-fold. Given a starting library of 10^10^ to 10^15^ different compounds, an enriched ligand will be present at only 1 part in 10^7^ to 1 part in 10^12^. Confidently detecting such rare molecules is hard, even with the application of next-generation sequencing techniques. The problem is exacerbated when biologically-relevant selections with fold-enrichments much smaller than 1000-fold are utilized.

Ideally, it would be possible to evolve small-molecule ligands out of DNA-linked chemical libraries in exactly the same way that biopolymer ligands are evolved from nucleic acid and protein libraries. *In vitro* evolution techniques overcome the “needle in the haystack” problem because they utilize multiple rounds of selection, reproductive amplification and library re-synthesis. Repetition provides unbounded fold-enrichments, even for inherently noisy selections. However, repetition also requires populations that can self-replicate. A subset of the existing approaches for preparing DNA-linked small-molecule libraries, those based on DNA-programmed combinatorial chemistry, fulfill this requirement. Rather than just recording the addition of chemical moieties as Lerner and Brenner originally proposed, the DNA in DNA-programmed approaches acts to direct a chemical synthesis. The DNA brings an incipient small molecule and suitable chemical building blocks into physical proximity and induces covalent bond formation between them. In so doing, the naked DNA functions as a gene: it orchestrates the assembly of a corresponding small molecule gene product. DNA genes that program highly fit small molecules can be enriched by selection, replicated by PCR, and then re-translated into DNA-linked chemical progeny. Whereas the Lerner-Brenner style DNA-linked small-molecule libraries are sterile and can only be subjected to selective pressure over one generation, DNA-programmed libraries produce many generations of offspring suitable for breeding.

One strategy for building a complex DNA-programmed chemical collection is to synthesize long polymers over many steps with a small alphabet of diversity elements. The resulting molecules then resemble biopolymers. Alternatively, a collection of equal complexity can be synthesized in a few steps using a very large alphabet of diversity elements. This latter synthetic scheme generates molecules of lower molecular weight. Although the numerical diversity of the two libraries may be equivalent, the utility of their constituent molecules in living organisms likely differs. Analysis of the World Drug Index suggests that collections of orally available pharmaceuticals are biased towards compounds with molecular weights under 500 Daltons. [8] For such molecules, the large-alphabet library is preferable. However, it is also more technically challenging to create this type of library because it requires that DNA genes direct covalent bond formation with hundreds to thousands of alternative chemical building blocks.

**Figure 1:**
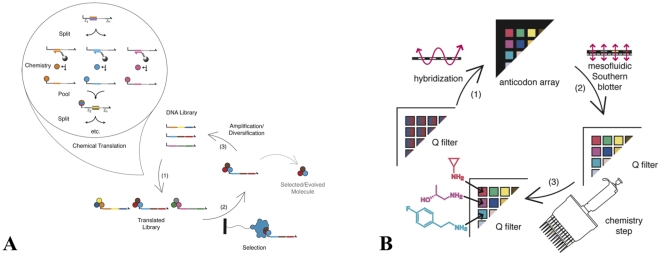
Small molecule evolution by DNA-programmed combinatorial chemistry. (**A**) A degenerate library of DNA sequences is chemically translated into small molecule-DNA conjugates. The attached small molecule corresponds to the structure encoded by the DNA “gene”. The translated molecules are selected for a desired trait, such as binding to a protein of interest. The encoding DNA is amplified and diversified. The cycle is iterated to yield small molecules with the selected property. (**B**) Translation of a single coding position by DNA-programmed combinatorial chemistry. A degenerate DNA library is split by hybridization to oligonucleotide-conjugated resins arrayed in a 384-well cassette (the anticodon array). The DNA is then transferred in a one-to-one fashion onto an anion-exchange chemistry array using a mesofluidic Southern blotter. The transferred DNA is subjected to a chemical coupling step, with a different chemical building block used in each well. The DNA is eluted and pooled. These operations are repeated until all of the coding positions in the gene have been read.

We previously reported the development of mesofluidic devices and arrayed supports for the routing and chemical modification of complex DNA populations in a 384-well plate format.[23] In principle, these devices can be used to build chemical collections that use a large-alphabet library through a DNA-programmed version of the classical “split-pool” technique (Figure 1). The process begins with DNA “genes” of varying sequence linked via polyethylene glycol to a synthetic nucleus (for example, a primary amine). The DNA genes are physically partitioned into sub-pools by hybridization of the codon at the first coding position of each gene to oligonucleotide-derivatized resins patterned in an “anticodon array.” The sub-pools are then transferred to separate wells of a 384-feature anion-exchange chemistry array. While the DNA is bound to the chemistry array by charge interactions, chemical transformations are performed on the associated synthetic nucleus. The organic chemistry is typically carried out under conditions that are incompatible with DNA solubility and with DNA base pairing. A different chemical building block is used at each of the 384 features. These operations constitute a “read” of the first coding position in a DNA-programmed synthesis. Additional reads of the remaining coding positions in the DNA genes are performed to complete the assembly of a small-molecule library. The process is analogous to the split-pool technique used for conventional combinatorial chemistry on polystyrene beads, with the exception that the physical separation of molecules into sub-pools is directed by DNA.

The accuracy of the DNA-directed partitioning and the efficiency of the encoded chemical conversions determine the fidelity with which DNA genes are translated into small molecule-DNA hybrids. Below, we demonstrate the use of the new array supports and mesofluidic devices to perform multistep, DNA-programmed chemical translation.

## Results

### Genes for a large-alphabet genetic code

**Figure 2:**
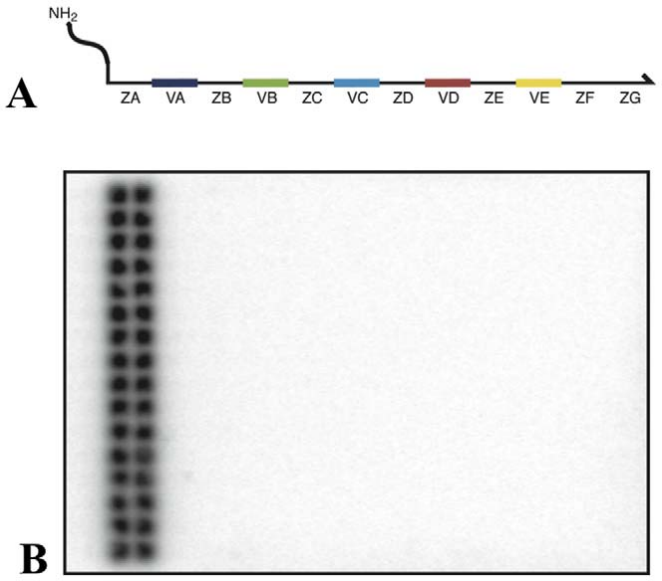
Assembly and characterization of a degenerate DNA library. (**A**) Structure of the DNA genes. The DNA genes consist of five variable positions (VA, VB, VC, VD, VE) flanked by six constant regions (ZA, ZB, ZC, ZD, ZE, ZF). The gene library was assembled with 384 different codon sequences at each of the VA–VD positions and 10 possible codon sequences at the final VE position. (**B**) Test of hybridization specificity. A sub-library of DNA genes with 32 distinct codon sequences at the B coding position (corresponding to the third and fourth columns of the B anticodon array) and 384 distinct codon sequences at the other coding positions (1194 possible codons) was hybridized to the B anticodon array, which holds 384 different oligonucleotide-conjugated resins. After hybridization, the array was imaged using a phosphor screen. Strong and roughly equal signal intensities are observed at the wells in the third and fourth columns of the anticodon array. No signal over background noise is observed at the other wells of the array.

To encode a library of high diversity, we constructed 217 billion different DNA gene sequences. The genes were 240 base pairs long comprising five coding positions that we denote A–E. Three-hundred and eighty-four different codon sequences were used at each of the first four coding positions, and ten at the last coding position (Figure 2a). The library was assembled from 3092 40-mer oligonucleotides using cross-over PCR. Coding positions A–D theoretically encode a complexity of 384^4^≈2*10^10^.

We determined the sequence of 4.6 million distinct genes from the assembled library to characterize how well it covered “genetic space”. Ninety-seven percent of the gene sequences occurred only once (the mean sequence count was 1.03), and the most abundant gene sequence occurred one hundred times. Every possible codon was observed at each coding position. Codon usage, however, deviated significantly from an expectation of random sampling with equal probability. The codon usage histograms followed a log-normal distribution, with one standard deviation in log-likelihood corresponding to two-to-three fold differences in codon frequency (Figure S1). Importantly, no correlation existed between codon identities at any pair of coding positions. Thus, the likelihood of any particular gene sequence can be well approximated by the product of the likelihoods of its constituent codons. Based on this approximation, 36% of all possible genes would be present at 100 copies or more in a 10 picomole aliquot of library material, 78% of the genes would be present at 10 copies or more, and 4% of the genes would be absent. A typical selection experiment (10 picomoles of starting material) would thus sample most of the attainable diversity.

### Hybridization specificity of a large-alphabet library

Previously reported DNA-programmed split-pool techniques were based on ∼80 different codon sequences.[9] Our large-alphabet library, however, contains 1546 different codons. We were unsure whether hybridization specificity would remain high despite the twenty-fold increase in codon diversity. To test hybridization specificity, we assembled a “drop-out” library in which 352 of the 384 B codons were omitted (see methods), while a full set of codons was retained at all other positions (1194 codons total). Radiolabelled ssDNA genes from this drop-out library were then hybridized to an array containing 384 oligonucleotide-coupled “anticodon” resins with sequences complementary to the B codon set (B1′–B384′) (Figure S2). The array was imaged on a phosphor screen (Figure 2). Inadequate hybridization specificity would be indicated by radioactive signals at positions corresponding to the 352 anticodons that were not included in the assembly, or by the absence of strong signals at the positions corresponding to the 32 B codons that were included in the assembly. As shown in Figure 2b, genes from the drop-out library hybridized at each of the 32 anticodon positions in the third and fourth columns with roughly equal signal intensity. Importantly, no signal over background was detected at the other 352 anticodon positions.

### Fluidics of a chemical translation step

**Figure 3:**
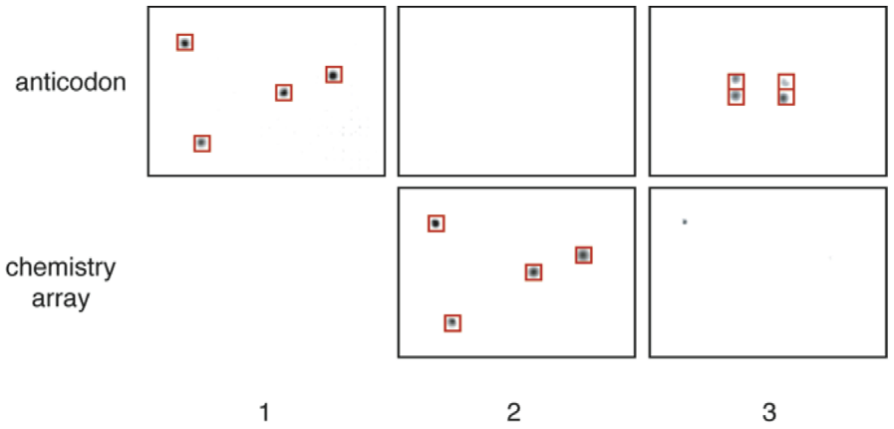
Accurate routing of DNA using mesofluidic devices. (**1**) Using the mesofluidic pump, four radiolabeled DNA sequences (ZA–A1, ZB–B2, ZC–C10, and ZD–D7) were hybridized to an anticodon array filled with 96 oligonucleotide-conjugated resins. The four spots at well positions A1, E19, G13, and O3 of the array (indicated by red boxes) were filled with resins A1′, B2′, C10′ and D7′ respectively. (**2**) Hybridized DNA was transferred to an anion-exchange chemistry array using a mesofluidic Southern blotter. (**3**) The DNA was eluted from the anion-exchange chemistry array, pooled, and split again using an anti-codon array containing the same 96 resins in a different order: well positions G9, G15, I9, and I15 of the array (indicated by red boxes) were filled respectively with the A1′, B2′, C10′, and D7′ resins. The arrays were imaged at each step.

We next checked if a full cycle of splitting by DNA hybridization and subsequent DNA blotting onto anion-exchange arrays proceeds accurately. For this test, we arbitrarily chose four 40-mer oligonucleotides: ZA–A1, ZB–B2, ZC–C10 and ZD–D7 (Fig. 2a). A mesofluidic pump was used to hybridize the radiolabelled oligonucleotides to an array containing anticodons A1′–A96′ substituted at different positions with anticodon resins corresponding to each of the 40-mers (Figure 3). Following hybridization, a mesofluidic Southern blotter was used to transfer the oligonucleotides onto an anion-exchange chemistry array. The oligonucleotides were then eluted from the chemistry array, pooled and hybridized to a second anticodon array with a different substitution pattern. At each hybridization or blotting step, the arrays were imaged on a phosphor screen. The images exhibited the expected labeling patterns, demonstrating that the DNA strands were correctly routed and transferred.

**Figure 4:**
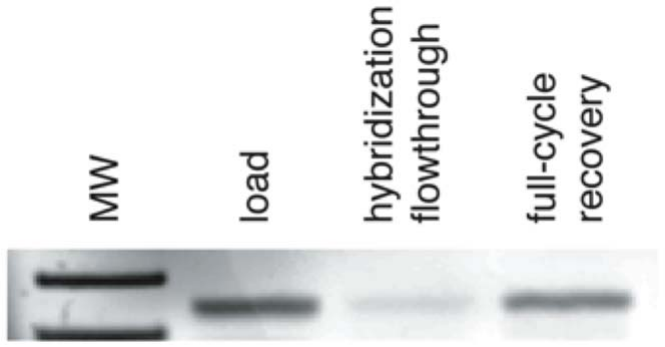
Yield of fluidic steps. A single DNA gene comprising the A1, B1, C37, D1 and E1 codons was hybridized to the A anticodon array, transferred to an anion-exchange chemistry array, eluted from that array, and then hybridized to the B anticodon array. Nucleic acid was isolated from the upper left well (corresponding to the B1′ anticodon) and quantified. Approximately 85% of the routed DNA was recovered after this routing cycle.

A remaining unknown was the overall yield for the sequence of fluidic steps. To measure yield, we constructed a single gene from codons A1, B1, C37, D1 and E1. This gene was hybridized to the A anticodon array, transferred to an anion-exchange chemistry array, eluted from the chemistry array, and then hybridized to the B anticodon array. DNA isolated from the B1′ anticodon position (which would only include material that was correctly routed in the second hybridization step) was analyzed by ethidium-stained agarose gel electrophoresis (Figure 4). As compared to an equivalent quantity of starting material, we determined that ∼85% of the routed DNA had been recovered after two hybridization steps, one blotting step and one elution step.

### A “zeroth-order” selection experiment

**Figure 5:**
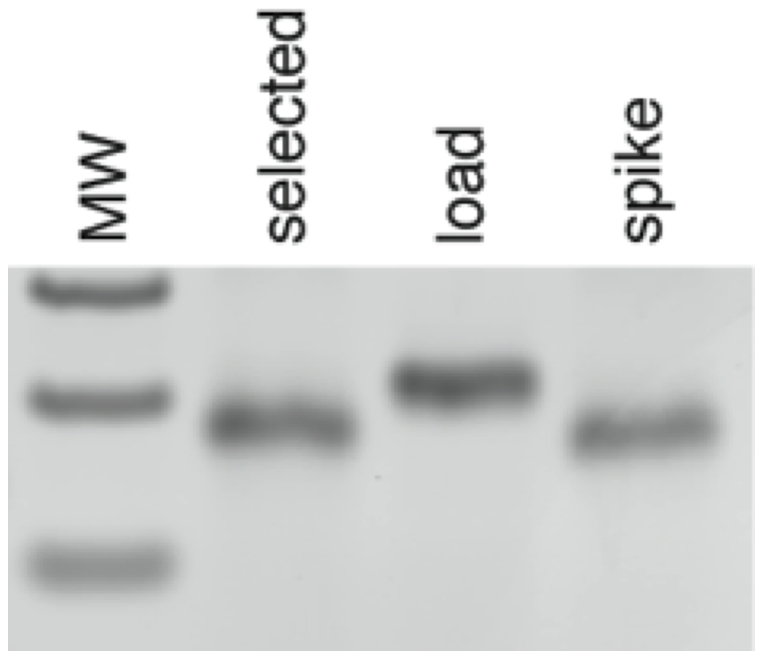
Proof-of-principle chemical selection. A degenerate library of DNA genes lacking one of the 1546 codons (C37) was mixed at 383 parts to 1 part with a short gene encoding C37 and missing one coding (VD) and one constant (ZD) position. The DNA solution was split over the C anticodon array and then blotted onto an anion-exchange chemistry array. The amine “synthetic nucleus” on the DNA in each well was acylated with chloroacetic acid. Propylamine was coupled to the DNA molecules in 383 of the wells, while the DNA in well G1 (corresponsing to the C37′ anticodon) was coupled to biotin hydrazide. The resulting peptoid-DNA conjugates were eluted, pooled and selected for binding to streptavidin-coated magnetic beads. The isolated material was amplified and analyzed on a 3% agarose gel, revealing a 13,000-fold enrichment of the sequence encoding the biotin monomer.

Highly parallel chemical translation was developed in order to facilitate the *in vitro* evolution of small molecules. To assess whether this goal was achieved, we performed a proof-of-principle chemical selection experiment. The experiment utilized a drop-out library containing all codons except C37 (1545 total codons). A “short gene” constructed from codons A1, B1, C37 and E1, but lacking the D codon and the ZD sequence, was also constructed. The 200 base-pair short gene could be distinguished from the 240 base-pair full-length genes by agarose gel electrophoresis. To create an initial genetic population, the short gene was mixed with the drop-out library genes in a ratio of 1∶384. The mixed genes were then split over the C anticodon array and transferred onto an ion-exchange chemistry array. At all positions except C37′, a propylamine peptoid monomer was coupled to the primary amine nucleus present at the 5′-terminus of each gene. At position C37′, however, biotin hydrazide was substituted for propylamine as the peptoid building block. The resulting peptoid-DNA conjugates were eluted, pooled and subjected to selection with streptavidin-coated magnetic beads. The selected material was PCR amplified and analyzed by agarose gel electrophoresis (Figure 5). While the ratio of short to long genes was initially 1∶384, it shifted to >35∶1 in the selected DNA, representing a lower limit of a 13,000-fold enrichment for the biotin-encoding sequence. To independently measure the composition of the selected DNA population, we sub-cloned the genes and Sanger sequenced 20 isolates. All of the isolates contained the C37 codon.

Aside from demonstrating that highly parallel split-pool chemical translation is suitable for *in vitro* chemical evolution, the 13,000-fold enrichment establishes some important basic points. First, it shows that the short gene is being routed to the correct positions on the anticodon and anion-exchange arrays with a specificity of at least 13,000-fold. Any lower specificity would have resulted in more of the 383 alternate gene sequences being biotinylated and enriched. Second, it shows that the individual features of the anion-exchange chemistry array remain well isolated during chemical coupling steps. A lack of isolation would have resulted in enrichment of genes hybridizing to the anticodon features adjacent to C37′. Finally, it shows that peptoid chemistry as adapted previously for synthesis on DNA[9] does not prevent the subsequent amplification of selected DNA genes.

### Multistep, DNA-programmed chemical translation

**Figure 6:**
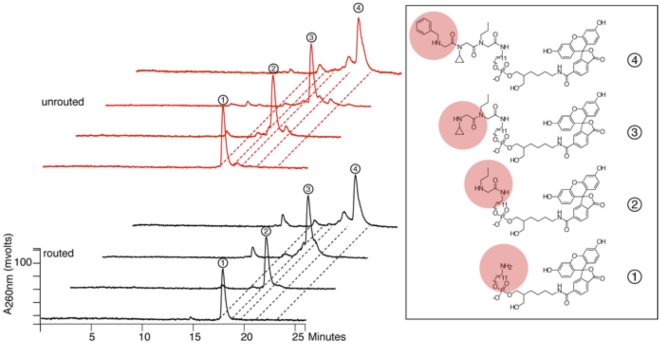
Analysis of a routed chemical translation. A single DNA gene comprising the A1, B1, C37, D1 and E1 codons was routed to the upper left well of the A anticodon array (hybridization of the first coding position to the A1′ anticodon) where a propylamine peptoid monomer was added. Similar reads of the second coding position (hybridization to the B1′ anticodon and addition of cyclopropylamine) and third coding position (hybridization to the C37′ anticodon and addition of benzylamine) completed the DNA-programmed synthesis. For comparison purposes, an identical synthesis was carried out without routing. Synthetic intermediates and products were digested with phosphodiesterase I and analyzed by reverse-phase HPLC. The major peaks for the routed products were isolated and analyzed by LC-MS (product 2 [M+H]^+^, expected 868.40, observed 868.75; product 3 [M+H]^+^, expected 965.45, observed 965.86; product 4 [M+H]^+^, expected 1112.52, observed 1112.97).

While the simplified selection experiment indicates that a number of aspects of the chemical translation process are functional, it does not provide a measure of yield for a multistep chemical translation. To obtain an estimate of yield, we analyzed the synthesis of a tri-peptoid molecule at the 5′-amine terminus of a gene. Because conjugates of small molecules with 240 base-pair DNA fragments are difficult to resolve by reverse-phase HPLC, fluorescein-labeled genes were used, and the peptoid-DNA conjugates were digested with phosphodiesterase I before analysis. This digestion step leaves the synthetic peptoid intermediate coupled to fluorescein (Figure 6), which can be separated by HPLC and quantified using a fluorescence detector.

The tripeptoid synthesis was performed on the gene sequence assembled from codons A1, B1, C37, D1 and E1. It was carried out twice, once with routing (splitting over the A, B and C anticodon arrays with subsequent blotting to an anion-exchange chemistry array) and once without routing (complete synthesis on one anion-exchange chemistry array). The intermediates (the mono-, di-, and tri-peptoids) produced at each synthetic step were analyzed by HPLC, and the major peaks were isolated and massed by LC-MS. As shown in Figure 6, the intermediates produced with and without routing exhibited identical HPLC retention times. All of the masses corresponded to the expected products. The three-step routed synthesis generated the expected tri-peptoid in 59% yield, with 81% of the starting DNA recovered (22% of the material was alternate products). The unrouted synthesis produced the expected tri-peptoid in 66% yield, with 98% of the starting DNA recovered (32% of alternate products). These data provide independent evidence that the highly parallel chemical translation process occurs with efficient routing and chemical conversion.

## Discussion

Exploration of large chemical spaces for molecules with novel and desired activities will continue to be a useful approach in academic studies and pharmaceutical investigations. Towards this end, DNA-programmed combinatorial chemistry facilitates a more rapid and efficient search process over a larger chemical space than does conventional high-throughput screening. However, for DNA-programmed combinatorial chemistry to be widely adopted, a high-fidelity, robust and general translation system must be available. This paper demonstrates a solution to that challenge.

The parallel chemical translation process described above is flexible. The devices and procedures are modular and can be used to divide a degenerate DNA population into a number of distinct sub-pools ranging from 1 to 384 at each step. This coding capacity opens the door for a wealth of chemical options and for the inclusion of diversity elements with widely varying size, hydrophobicity, charge, rigidity, aromaticity, and heteroatom content, allowing the search for ligands in a “hypothesis-free” fashion. Alternatively, the capacity can be used to elaborate a variety of subtle changes to a known compound and exhaustively probe structure-activity relationships. In this case, some elements in a synthetic scheme can be diversified while others are conserved (for example, chemical elements known to have a particular structural or electrostatic constraint, modular chemical fragments that independently bind to a protein target, metal chelating functional groups, fluorophores). By facilitating the synthesis and testing of varied chemical collections, the tools and methods reported here should accelerate the application of “designer” small molecules to problems in basic science, industrial chemistry and medicine.

## Materials and Methods

### Materials

Chemicals and solvents were purchased from Acros (Geel, Belgium), Alfa Aesar (Ward Hill, MA, USA), Novabiochem (La Jolla, CA, USA), Oakwood Chemical (West Columbia, SC, USA), Sigma-Aldrich (St. Louis, MO, USA), TCI America (Portland, OR, USA), VWR International (West Chester, PA, USA), or from the supplier indicated.

### General methods

HPLC analysis of peptoid reactions was performed on a Microsorb reverse-phase C18 analytical column (Varian; Palo Alto, CA, USA) heated to 50°C and monitored either at 260 and 280 nm using a UV detector (Spectra Focus, Spectra-Physics; Mountain View, CA, USA) or using a fluorescence detector (Spectra System FL2000, Thermo Separations Products; Saint Peters, MO) with emission and excitation wavelengths set at 488 nm and 518 nm. Linear gradients between 100 mM triethylammonium acetate pH 5.5 and 100 mM triethylammonium acetate pH 5.5, 90 percent acetonitrile were used. Peptoid-DNA hybrids were digested with phosphodiesterase I in 100 mM Tris pH 8.9, 100 mM NaCl, 15 mM MgCl_2_ for two hours at room temperature and analyzed on a Micromass ZQ LC-MS at the Vincent Coates Foundation Mass Spectrometry Laboratory (Stanford, CA, USA).

Degenerate library assembly and ssDNA preparation were performed as described previously. [24] Gasketed Q cellulose chemistry arrays and microcolumn anticodon arrays were prepared as described.[23] The hybridization, Southern blotting, and peptoid coupling steps were also performed as described.[23] Radioactive arrays were imaged with a storage phosphor screen (Molecular Dynamics; Sunnyvale, CA, USA) and a Typhoon 9400 reader (General Electric; Fairfield, CT, USA).

### Assembly of a partially degenerate library

The degenerate library used to test hybridization specificity was assembled using the fully degenerate set of codons for the A, C, D, and E positions and codons B2, B14, B26, B38, B50, B62, B74, B86, B98, B110, B122, B134, B146, B158, B170, B182, B194, B206, B218, B230, B242, B254, B266, B278, B290, B302, B314, B326, B338, B350, B362, and B374 at the B position. See Supporting Information S1 for the sequences of the codons.

### Selection for streptavidin binding

The sequence used to assemble the short gene was:

5′-ATGGTATCAAGCTTGCCACAGCCGAAGCAGACTTAATCACGTCGAGCTCTCTACTGCATAGATTAGCGTACATAGGCCCGGAACCCGGGACAAGGTGTCATGAGGTCTAACATCAGCTCCGTAATTCTGTACAGGTCGCGATAATCAGCGGGAATCAGGCGGCAGAATCTCGAGTACTAGAGAGCATGCACATATCTCCCTATAGTGAGTCGTATTAAGCGC-3′. The degenerate library material consisted of the sequence:

5′-ATGGTATCAAGCTTGCCACAXXXXXXXXXXXXXXXXXXXXGTCGAGCTCTCTACTGCATAXXXXXXXXXXXXXXXXXXXXGAACCCGGGACAAGGTGTCAXXXXXXXXXXXXXXXXXXXXTAGTGGCCTGCAGCTATGTAXXXXXXXXXXXXXXXXXXXXGTAATTCTGTACAGGTCGCGXXXXXXXXXXXXXXXXXXXXGGCAGAATCTCGAGTACTAGAGAGCATGCACATATCTCCCTATAGTGAGTCGTATTAAGCGC-3′. Groups of 20 adjacent X's represent the 20-base pair variable regions.

Biotinylated DNA was mixed with 2.5 μg BSA, 1 μg tRNA, and 2 μl of μMACS Streptavidin Magnetic Beads from the μMACS Streptavidin Kit (Miltenyi Biotec; Bergisch Gladbach, Germany). The solution was loaded onto a Miltenyi μColumn, and the column was washed four times with 100 μl of 10 mM Tris pH 7.4, 1 mM EDTA, 1 M NaCl and four times with 100 μl of 10 mM Tris pH 7.4, 1 mM EDTA, 100 mM NaCl. The beads were eluted in 150 μl 10 mM Tris pH 8.0, 1 mM EDTA and were used as the template for a PCR reaction with Phusion polymerase using the following primers: 5′-ATGGTATCAAGCTTGCCACA-3′ and 5′-CTAGTACTCGAGATTCTGCC-3′. The amplified material was run on a 3% agarose gel and imaged with ethidium bromide.

### Multistep chemical translation

The gene sequence used for the translation was:

5′-ATGGTATCAAGCTTGCCACAGCCGAAGCAGACTTAATCACGTCGAGCTCTCTACTGCATAGATTAGCGTACATAGGCCCGGAACCCGGGACAAGGTGTCATGAGGTCTAACATCAGCTCCTAGTGGCCTGCAGCTATGTAAATCACGCTTGGTAAGTTGGGTAATTCTGTACAGGTCGCGATAATCAGCGGGAATCAGGCGGCAGAATCTCGAGTACTAGAGAGCATGCACATATCTCCCTATAGTGAGTCGTATTAAGCGC-3′. The primer used for single-stranded DNA production was 5′-NFATGGTATCAAGCTTGCCACA-3′, where N denotes a 5′-amino-modifier C12 phosphoramidite (#10–1912; Glen Research, Sterling, VA, USA) and F denotes a 6-fluorescein phosphoramidite (#10–1964; Glen Research, Sterling, VA, USA).

## Supporting Information

Figure S1 **Genes for a large-alphabet genetic code.** Genes were constructed from four coding positions (VA-VD) with 384 distinct codons per position. Roughly 4.6 million isolates were sequenced. (**A**) Histograms of codon usage at each coding position. The x-axis is the natural logarithm of codon frequency: the number of times (out of the ∼4.6 million gene reads) that a given codon was observed. The y-axis shows how many of the 384 codons fall into each of the frequency bins on the x-axis. (**B**) Codon usage at different coding positions is uncorrelated. 384^2^ = 147,456 possible pairs of codons can exist at two different coding positions in a gene. Given uncorrelated random sampling, the average number of co-occurrences of a given pair, N, is the product of the two individual codon likelihoods with 4.6 million. The standard deviation from this average is N^1/2^ (the standard deviation of the Poisson distribution that results from finite sampling). The x-axis of each histogram plot shows the difference between the experimentally observed number of co-occurrences and the predicted number of co-occurrences expressed in units of standard deviation or Z-score. The y-axis shows how many of the147,456 codon pairs fall into each of the Z-score bins on the x-axis. For each graph, the two coding positions from which the data are derived are indicated in the upper left corner. Note the good agreement between the observed data (red) and the expectation for uncorrelated random sampling (the unit normal distribution plotted in black).
(TIFF)

Figure S2 **Schematic of the plating arrangement for the VB anticodon arrays.** The other variable regions were plated in the same order. Four 96-well plates of oligonucleotide-conjugated resin (red contains B1′–B96′; blue, B97′–B192′; light green, B193′–B288′; and gray, B289′–B384′) are plated in a zigzag fashion in a 394-well anticodon array. The upper left corner of the 384-well anticodon array is shown.
(TIFF)

Supporting Information S1 **Sequences used in the library assembly.**
(DOCX)
