# Supplementary figures and images for: Highly Parallel Translation of DNA Sequences into Small Molecules
Source: PLoS One. 2012 Mar 29;7(3):e28056. doi: 10.1371/journal.pone.0028056 (PMC3315553; doi:10.1371/journal.pone.0028056)

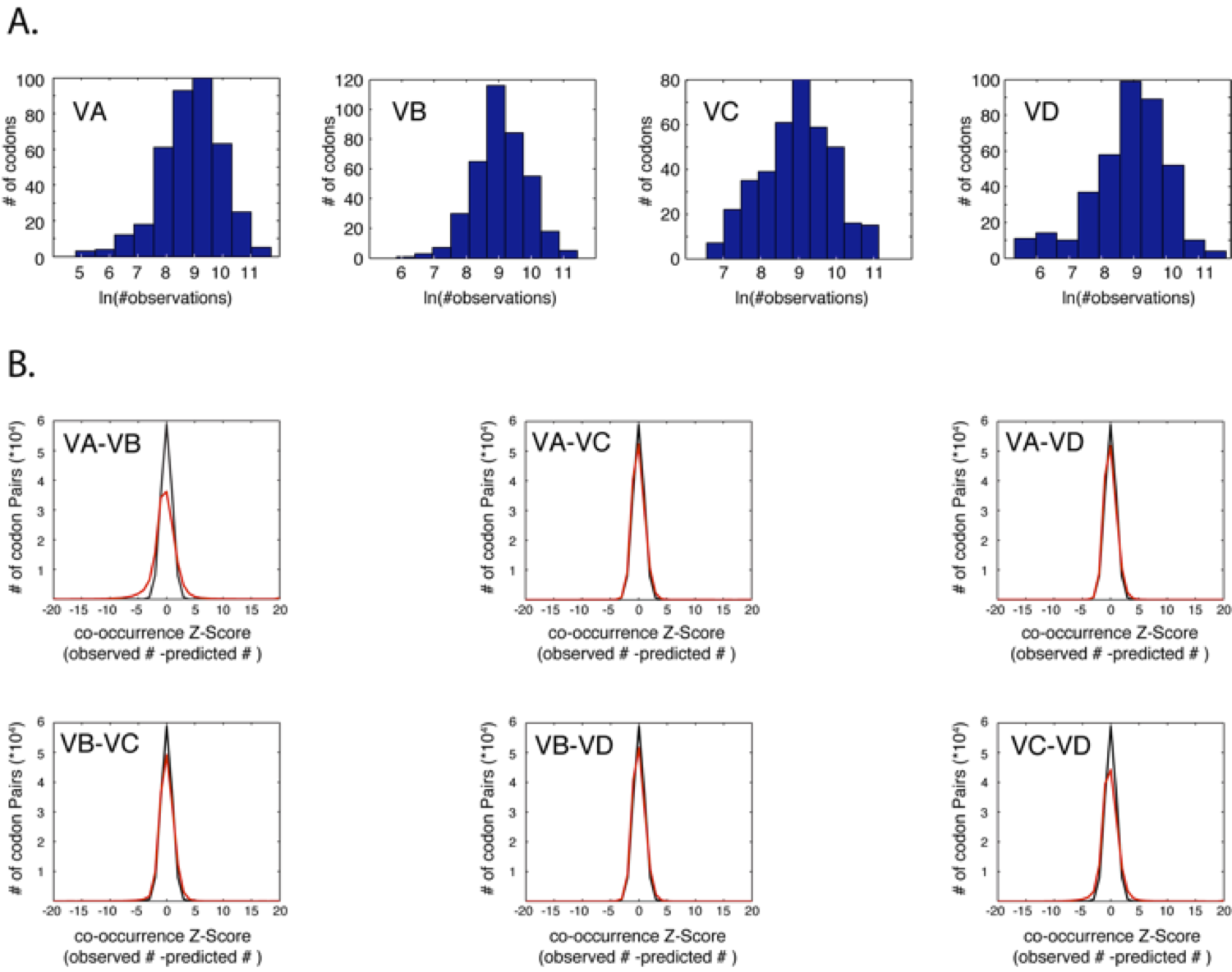

Supplement: Figure S1 — Genes for a large-alphabet genetic code. Genes were constructed from four coding positions (VA-VD) with 384 distinct codons per position. Roughly 4.6 million isolates were sequenced. (A) Histograms of codon usage at each coding position. The x-axis is the natural logarithm of codon frequency: the number of times (out of the ∼4.6 million gene reads) that a given codon was observed. The y-axis shows how many of the 384 codons fall into each of the frequency bins on the x-axis. (B) Codon usage at different coding positions is uncorrelated. 3842 = 147,456 possible pairs of codons can exist at two different coding positions in a gene. Given uncorrelated random sampling, the average number of co-occurrences of a given pair, N, is the product of the two individual codon likelihoods with 4.6 million. The standard deviation from this average is N1/2 (the standard deviation of the Poisson distribution that results from finite sampling). The x-axis of each histogram plot shows the difference between the experimentally observed number of co-occurrences and the predicted number of co-occurrences expressed in units of standard deviation or Z-score. The y-axis shows how many of the147,456 codon pairs fall into each of the Z-score bins on the x-axis. For each graph, the two coding positions from which the data are derived are indicated in the upper left corner. Note the good agreement between the observed data (red) and the expectation for uncorrelated random sampling (the unit normal distribution plotted in black). (TIFF) [file pone.0028056.s001.tif]

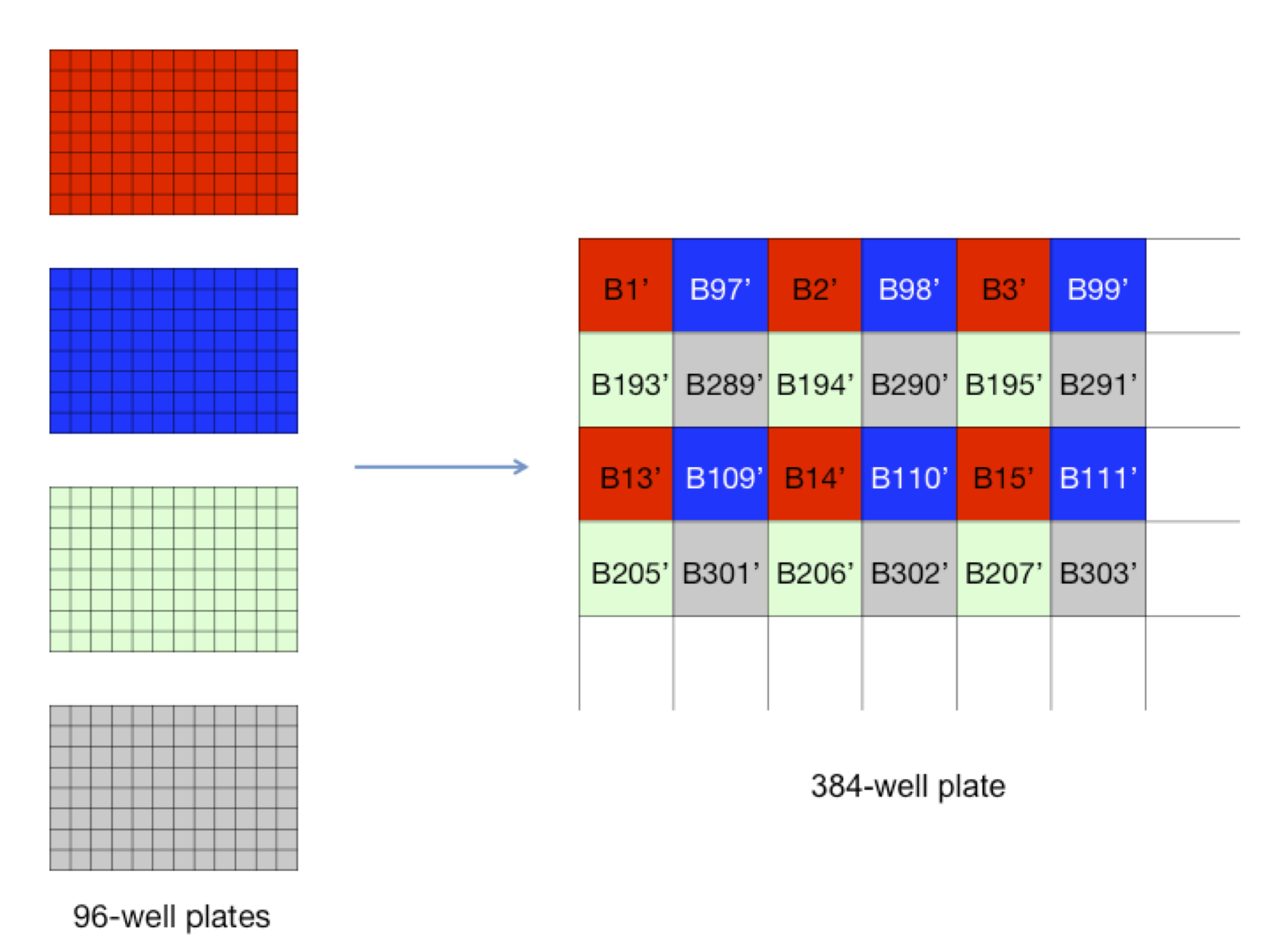

Supplement: Figure S2 — Schematic of the plating arrangement for the VB anticodon arrays. The other variable regions were plated in the same order. Four 96-well plates of oligonucleotide-conjugated resin (red contains B1′–B96′; blue, B97′–B192′; light green, B193′–B288′; and gray, B289′–B384′) are plated in a zigzag fashion in a 394-well anticodon array. The upper left corner of the 384-well anticodon array is shown. (TIFF) [file pone.0028056.s002.tif]
