# Supplementary material for: Highly Parallel Translation of DNA Sequences into Small Molecules
Source: PLoS One. 2012 Mar 29;7(3):e28056. doi: 10.1371/journal.pone.0028056 (PMC3315553; doi:10.1371/journal.pone.0028056)
Supplement: Supporting Information S1 — Sequences used in the library assembly. (DOCX) [file pone.0028056.s003.docx]

**Supporting Information S1**

| ZAA001 | ATGGTATCAAGCTTGCCACAGCCGAAGCAGACTTAATCAC |
| --- | --- |
| ZAA002 | ATGGTATCAAGCTTGCCACATTAGGCACCACAGTACGAAC |
| ZAA003 | ATGGTATCAAGCTTGCCACAGCAGAGACCATGTTAGCAAC |
| ZAA004 | ATGGTATCAAGCTTGCCACAGGAAGTCAACTGTGAGCAAC |
| ZAA005 | ATGGTATCAAGCTTGCCACAACATCAAGACCACGTTGCTA |
| ZAA006 | ATGGTATCAAGCTTGCCACAGATCCAAGCGTAGCCAACTA |
| ZAA007 | ATGGTATCAAGCTTGCCACAAGCCGCAATGACAGCTTATA |
| ZAA008 | ATGGTATCAAGCTTGCCACATCCCAGAAGACGCAATTTCA |
| ZAA009 | ATGGTATCAAGCTTGCCACATCCGAATTAACAATCCGGCA |
| ZAA010 | ATGGTATCAAGCTTGCCACATGTACCACGAAATCATGCCA |
| ZAA011 | ATGGTATCAAGCTTGCCACAGATAAGAGCACAGCTTCCCA |
| ZAA012 | ATGGTATCAAGCTTGCCACACCGACCCTTCGAGTTTAGTT |
| ZAA013 | ATGGTATCAAGCTTGCCACAACCGTCCGATTCCGTTAATT |
| ZAA014 | ATGGTATCAAGCTTGCCACAAAAGTCCGGTTAATTTGCGG |
| ZAA015 | ATGGTATCAAGCTTGCCACACGGGTGTAGAAACCGACTAA |
| ZAA016 | ATGGTATCAAGCTTGCCACAAACGGTTGCTAATGTTTCCG |
| ZAA017 | ATGGTATCAAGCTTGCCACATAATACTCCGTAAGACCGCG |
| ZAA018 | ATGGTATCAAGCTTGCCACATAACTAATCCGAAGCGGTCC |
| ZAA019 | ATGGTATCAAGCTTGCCACATGTTAAACCAACGGCCTACA |
| ZAA020 | ATGGTATCAAGCTTGCCACAGCACAACAAAGACCGTTTCT |
| ZAA021 | ATGGTATCAAGCTTGCCACACACTTGAGGTTGCGGAATTT |
| ZAA022 | ATGGTATCAAGCTTGCCACACTCGCGTAGGTTAGACTGTT |
| ZAA023 | ATGGTATCAAGCTTGCCACACTTGCTAGGCGTTAGGACTT |
| ZAA024 | ATGGTATCAAGCTTGCCACACCGCCCGTTTGCATAAATAT |
| ZAA025 | ATGGTATCAAGCTTGCCACACGCGAATACGTTTGTCCAAT |
| ZAA026 | ATGGTATCAAGCTTGCCACACCGGTGTAATAACAGCGAAC |
| ZAA027 | ATGGTATCAAGCTTGCCACACCCGGAAGGCAATTACACTA |
| ZAA028 | ATGGTATCAAGCTTGCCACAAAAGAACTTCCAGTGCCGAT |
| ZAA029 | ATGGTATCAAGCTTGCCACAAAGTCACGTTAAGGCCAGAG |
| ZAA030 | ATGGTATCAAGCTTGCCACAAAACTGGTCTGAACGTCCTC |
| ZAA031 | ATGGTATCAAGCTTGCCACAAAAGGTCGTTCAATGTTCGC |
| ZAA032 | ATGGTATCAAGCTTGCCACAAGGATTTCTCGAACTACCGC |
| ZAA033 | ATGGTATCAAGCTTGCCACAAAGTGAGTTCAACGTCCTCC |
| ZAA034 | ATGGTATCAAGCTTGCCACAGCGTGCAGTTTGTTAAGACT |
| ZAA035 | ATGGTATCAAGCTTGCCACAGCAGGTTCACGACTACTTTG |
| ZAA036 | ATGGTATCAAGCTTGCCACAGCACTTGTGTAACTACGTCG |
| ZAA037 | ATGGTATCAAGCTTGCCACAGATCCACGGACCTCGATTAG |
| ZAA038 | ATGGTATCAAGCTTGCCACAGTGTCTAACCACAAAGCGAC |
| ZAA039 | ATGGTATCAAGCTTGCCACAGCCGAACCTTCAACAGAGTA |
| ZAA040 | ATGGTATCAAGCTTGCCACACTCCGGTCGATGGATGAATT |
| ZAA041 | ATGGTATCAAGCTTGCCACACACTCTGCGTTAATGTTGGG |
| ZAA042 | ATGGTATCAAGCTTGCCACACAGCTTCTCTAAGTTTGCCG |
| ZAA043 | ATGGTATCAAGCTTGCCACATATACCGTCCGAGGTGATGT |
| ZAA044 | ATGGTATCAAGCTTGCCACATCGGAGGCTATACTGTACGT |
| ZAA045 | ATGGTATCAAGCTTGCCACATCCTTCGGAGTCGATGTAGT |
| ZAA046 | ATGGTATCAAGCTTGCCACATCTATACGAGTACGTGGGCT |
| ZAA047 | ATGGTATCAAGCTTGCCACATCTCGGTACAGAGTTTGACG |
| ZAA048 | ATGGTATCAAGCTTGCCACATCCACGCAGAGGAGTTAAAG |
| ZAA049 | ATGGTATCAAGCTTGCCACATAATTGTAGCGAAGGGCTCC |
| ZAA050 | ATGGTATCAAGCTTGCCACATCCCGCGAAGTAAACATTCA |
| ZAA051 | ATGGTATCAAGCTTGCCACACACAGTAATGAACCGTTGCC |
| ZAA052 | ATGGTATCAAGCTTGCCACACATTACCGAACATGGCGTAC |
| ZAA053 | ATGGTATCAAGCTTGCCACACCACGACGAAGTGAATTGAC |
| ZAA054 | ATGGTATCAAGCTTGCCACACCACGGATAGAACTGAGGAC |
| ZAA055 | ATGGTATCAAGCTTGCCACACCGCGACATTAAGTCAAGAC |
| ZAA056 | ATGGTATCAAGCTTGCCACACCGTGCGTAGAATGAAGAAC |
| ZAA057 | ATGGTATCAAGCTTGCCACACGGCCTTGAATGCAACTTTA |
| ZAA058 | ATGGTATCAAGCTTGCCACACAGAGCGAAGAACCCTGTTA |
| ZAA059 | ATGGTATCAAGCTTGCCACACCGAATCGAGAACAGGCTTA |
| ZAA060 | ATGGTATCAAGCTTGCCACACCTATCCGCAAAGTGGAGAA |
| ZAA061 | ATGGTATCAAGCTTGCCACACGTTCTCGCAAAGCACTAAA |
| ZAA062 | ATGGTATCAAGCTTGCCACACCTTCGCGCAAAGTATGAAA |
| ZAA063 | ATGGTATCAAGCTTGCCACAAAGCCTCGCTGAGTATGTTT |
| ZAA064 | ATGGTATCAAGCTTGCCACAATATTCGCGTCGAGTGAGTT |
| ZAA065 | ATGGTATCAAGCTTGCCACAAAACCTCGCGTCATTGATCT |
| ZAA066 | ATGGTATCAAGCTTGCCACAAATGCGGATTCAGGTTAGCT |
| ZAA067 | ATGGTATCAAGCTTGCCACAAATTAGTTCTCATGCCGCCT |
| ZAA068 | ATGGTATCAAGCTTGCCACAAGGCTTACGATCCTCAGACT |
| ZAA069 | ATGGTATCAAGCTTGCCACAACTCGTGTGTAATCCGATGG |
| ZAA070 | ATGGTATCAAGCTTGCCACAACTCATTCGAGATGTTCGGG |
| ZAA071 | ATGGTATCAAGCTTGCCACAACTTCAGCTACAGCGTTAGG |
| ZAA072 | ATGGTATCAAGCTTGCCACAACCGCGCTCTAATACTTGAG |
| ZAA073 | ATGGTATCAAGCTTGCCACAAGACTTAGTGAACGTCGCTC |
| ZAA074 | ATGGTATCAAGCTTGCCACAAGAGCCACCGAATGTTCTAC |
| ZAA075 | ATGGTATCAAGCTTGCCACATCTCGTAGCAGACTCTAGGG |
| ZAA076 | ATGGTATCAAGCTTGCCACATAATCTCATCGAACCGGAGC |
| ZAA077 | ATGGTATCAAGCTTGCCACATAGAGTAGCACACCTTTGCC |
| ZAA078 | ATGGTATCAAGCTTGCCACATGACCATACACGAACTGGAC |
| ZAA079 | ATGGTATCAAGCTTGCCACAGGCTACTTCAGCGTCACTAT |
| ZAA080 | ATGGTATCAAGCTTGCCACAGCATCGACTTAATGGTCACG |
| ZAA081 | ATGGTATCAAGCTTGCCACAGCAGTCACGCTAAGTCTTTC |
| ZAA082 | ATGGTATCAAGCTTGCCACAGAAACACTACGCACATGGTC |
| ZAA083 | ATGGTATCAAGCTTGCCACAGCGCTCGGATAACATAACAC |
| ZAA084 | ATGGTATCAAGCTTGCCACAGCCGCCATCTAAGAATGAAC |
| ZAA085 | ATGGTATCAAGCTTGCCACAGAAGATCGTCCAACAGCCTA |
| ZAA086 | ATGGTATCAAGCTTGCCACAGCTGCATGAGAACCACTAAC |
| ZAA087 | ATGGTATCAAGCTTGCCACAGCGTATCAGCAAGACCCATA |
| ZAA088 | ATGGTATCAAGCTTGCCACATGTCATCTGAAACACGACCA |
| ZAA089 | ATGGTATCAAGCTTGCCACAAGGCGATGATGGCATTTACT |
| ZAA090 | ATGGTATCAAGCTTGCCACAGCCATGCCTAAATTGCTTCA |
| ZAA091 | ATGGTATCAAGCTTGCCACATGCATCAACAGACAGCTTCA |
| ZAA092 | ATGGTATCAAGCTTGCCACAAGGCTTCAGCGATTGTATGT |
| ZAA093 | ATGGTATCAAGCTTGCCACACAGCCGAGAGTGTGATACAT |
| ZAA094 | ATGGTATCAAGCTTGCCACAATACGTCATCTCACGAGCTG |
| ZAA095 | ATGGTATCAAGCTTGCCACACCGGTCAGAGAACTGCAATA |
| ZAA096 | ATGGTATCAAGCTTGCCACATATACTGATGCAGACGGCTG |
| ZAA097 | ATGGTATCAAGCTTGCCACAAAGCATTGCGAATCATAGCC |
| ZAA098 | ATGGTATCAAGCTTGCCACAGCATCGAATCAACGCCATTA |
| ZAA099 | ATGGTATCAAGCTTGCCACAAAGCCTCACTTGTCAGCAAT |
| ZAA100 | ATGGTATCAAGCTTGCCACACGGCGCGATTATGATGAAAT |
| ZAA101 | ATGGTATCAAGCTTGCCACAGCATACCACTGCACATTGAG |
| ZAA102 | ATGGTATCAAGCTTGCCACACTATCGAGGATCACGGACTG |
| ZAA103 | ATGGTATCAAGCTTGCCACAGATTCGATCACATCTCGTGC |
| ZAA104 | ATGGTATCAAGCTTGCCACAAGAGTGCTCCTCATCTACGT |
| ZAA105 | ATGGTATCAAGCTTGCCACAGATGACATTCCACGTTCTGC |
| ZAA106 | ATGGTATCAAGCTTGCCACAACACTGATGGAACAGGTAGC |
| ZAA107 | ATGGTATCAAGCTTGCCACAACTTAGCAGACACGAGTAGC |
| ZAA108 | ATGGTATCAAGCTTGCCACAAATCATGTCCAACTCGCGTA |
| ZAA109 | ATGGTATCAAGCTTGCCACATGTCATGCCACAAGATCACA |
| ZAA110 | ATGGTATCAAGCTTGCCACATGCCAGCTAAGACACAGTAC |
| ZAA111 | ATGGTATCAAGCTTGCCACACACATCGAGGCAATGGAGTA |
| ZAA112 | ATGGTATCAAGCTTGCCACACTTCAGTGGCTACGATGAGT |
| ZAA113 | ATGGTATCAAGCTTGCCACAGACTGCTGCTCATATCGAGT |
| ZAA114 | ATGGTATCAAGCTTGCCACAGCAGTTCGCATCATACCTTG |
| ZAA115 | ATGGTATCAAGCTTGCCACAGCATGACGTGTAAGCTATCG |
| ZAA116 | ATGGTATCAAGCTTGCCACAGCGCGGGATCAATTTAATGA |
| ZAA117 | ATGGTATCAAGCTTGCCACATGTCCACATACACGCAATGA |
| ZAA118 | ATGGTATCAAGCTTGCCACACCATCTGCGATCAGTTTGTG |
| ZAA119 | ATGGTATCAAGCTTGCCACAAGCATCGAGTGTAATTTGCG |
| ZAA120 | ATGGTATCAAGCTTGCCACAAGCTACCGAGTCGATCTGAT |
| ZAA121 | ATGGTATCAAGCTTGCCACATATCACTTAGGAGCTGTGCG |
| ZAA122 | ATGGTATCAAGCTTGCCACAAGATGCACTCTAACCGTCAG |
| ZAA123 | ATGGTATCAAGCTTGCCACATAATGCGTGCGAATGTATGC |
| ZAA124 | ATGGTATCAAGCTTGCCACACACACGCTACCAAGTATGGA |
| ZAA125 | ATGGTATCAAGCTTGCCACACCGCTAGAGCAAGTAATGGA |
| ZAA126 | ATGGTATCAAGCTTGCCACACTGCCAAGCAACTTAATGCA |
| ZAA127 | ATGGTATCAAGCTTGCCACACTCAGTCAGCTCTGCATGAT |
| ZAA128 | ATGGTATCAAGCTTGCCACAATACGGAGGTACATCGCATG |
| ZAA129 | ATGGTATCAAGCTTGCCACAAATATGCACTAACGCCATGC |
| ZAA130 | ATGGTATCAAGCTTGCCACAGCTTCATGCAAATCCACGAA |
| ZAA131 | ATGGTATCAAGCTTGCCACACATGGCATTGAAGCTCACTC |
| ZAA132 | ATGGTATCAAGCTTGCCACAACAGTGCGCCAATGTTACTA |
| ZAA133 | ATGGTATCAAGCTTGCCACAAAATTGCGATCAAGGCTCAC |
| ZAA134 | ATGGTATCAAGCTTGCCACAGAGACATCGTGCAACATCAC |
| ZAA135 | ATGGTATCAAGCTTGCCACACCTGCACGTCAAGAAGACTA |
| ZAA136 | ATGGTATCAAGCTTGCCACAATGACTGTGTGCCAGGATTT |
| ZAA137 | ATGGTATCAAGCTTGCCACAGAGTGCTGAACATTTCTGGC |
| ZAA138 | ATGGTATCAAGCTTGCCACAGCGATGCTCTCATTAGCTCT |
| ZAA139 | ATGGTATCAAGCTTGCCACATGAGCACGGCTATATCTGTG |
| ZAA140 | ATGGTATCAAGCTTGCCACACATGCTAAGCGATATGGCAG |
| ZAA141 | ATGGTATCAAGCTTGCCACACCCAAATGAAGAGTCGATGC |
| ZAA142 | ATGGTATCAAGCTTGCCACATATAGCCAGACATCAGCAGC |
| ZAA143 | ATGGTATCAAGCTTGCCACAGAATCAGCTCCAAGACGCTA |
| ZAA144 | ATGGTATCAAGCTTGCCACACGCATGTTGAAATATGGGCA |
| ZAA145 | ATGGTATCAAGCTTGCCACAAGGCGACTCATCACTATTGG |
| ZAA146 | ATGGTATCAAGCTTGCCACATCACGCTAGATCACGATTGG |
| ZAA147 | ATGGTATCAAGCTTGCCACATTCTACACCATGCAGGACAG |
| ZAA148 | ATGGTATCAAGCTTGCCACACATCTGATTGAAGCAGCGTC |
| ZAA149 | ATGGTATCAAGCTTGCCACACACATATCGACAAGGTCCGA |
| ZAA150 | ATGGTATCAAGCTTGCCACACAATGGAGTCTAGCTGGCTT |
| ZAA151 | ATGGTATCAAGCTTGCCACAGCTTGAGCGAAAGGCATTAA |
| ZAA152 | ATGGTATCAAGCTTGCCACAGCTCACGCGAAATCCATTAA |
| ZAA153 | ATGGTATCAAGCTTGCCACAGATGCCATGCCATTGTATCG |
| ZAA154 | ATGGTATCAAGCTTGCCACAGCAGTCAAGACCAGCTTCTA |
| ZAA155 | ATGGTATCAAGCTTGCCACACTCCGGCAGGACAATAATCA |
| ZAA156 | ATGGTATCAAGCTTGCCACAGCGCTATTCACAAGCAATCA |
| ZAA157 | ATGGTATCAAGCTTGCCACAAGACGTGCTGAACGAGTATC |
| ZAA158 | ATGGTATCAAGCTTGCCACAAAGCTCTTAGCATCTCGGTG |
| ZAA159 | ATGGTATCAAGCTTGCCACAACCGATGACAGTTCACAGAG |
| ZAA160 | ATGGTATCAAGCTTGCCACACTTACGAGCCTCAGGTAGTG |
| ZAA161 | ATGGTATCAAGCTTGCCACATACTGGATCGCATCGTAGTG |
| ZAA162 | ATGGTATCAAGCTTGCCACAACCTACGTCGCATTCTAGTG |
| ZAA163 | ATGGTATCAAGCTTGCCACAAGCATGTCTCTAACTGGACG |
| ZAA164 | ATGGTATCAAGCTTGCCACAAACCTCTAAGCATACGGCAG |
| ZAA165 | ATGGTATCAAGCTTGCCACAAGAGCTGTCAAACGGTGTAA |
| ZAA166 | ATGGTATCAAGCTTGCCACAACATGCGTTAAATCCTCGGA |
| ZAA167 | ATGGTATCAAGCTTGCCACAGCCGACTGGAAACTCATACA |
| ZAA168 | ATGGTATCAAGCTTGCCACAATGCCACCGTTGTCATAACT |
| ZAA169 | ATGGTATCAAGCTTGCCACATGAGAAACCTAACGGGTGTC |
| ZAA170 | ATGGTATCAAGCTTGCCACAAAGCTCCAGTCAGATCATCG |
| ZAA171 | ATGGTATCAAGCTTGCCACAAAATGTCGATTAAGCTGCGG |
| ZAA172 | ATGGTATCAAGCTTGCCACATGAATACCCTCGAAGCGATC |
| ZAA173 | ATGGTATCAAGCTTGCCACACGATATGCAGAATAGCGCAC |
| ZAA174 | ATGGTATCAAGCTTGCCACAGACGGAGACCTGTATGCAAT |
| ZAA175 | ATGGTATCAAGCTTGCCACAGCCGTCGCCAGAATATAAGA |
| ZAA176 | ATGGTATCAAGCTTGCCACATCTATATCGGCATGTGGCTG |
| ZAA177 | ATGGTATCAAGCTTGCCACATCGACCACTGACTGCATTAG |
| ZAA178 | ATGGTATCAAGCTTGCCACAGAGTAGAGTCCATCACGCTC |
| ZAA179 | ATGGTATCAAGCTTGCCACAGATGACTCTCCATGTCTCGG |
| ZAA180 | ATGGTATCAAGCTTGCCACAATACAGACTGCGACACTACG |
| ZAA181 | ATGGTATCAAGCTTGCCACAGTCCCGGAGTGACAAACATA |
| ZAA182 | ATGGTATCAAGCTTGCCACAACTGCATAGTCACTGTGGTG |
| ZAA183 | ATGGTATCAAGCTTGCCACAAGATGCTTCACAATTCCCGA |
| ZAA184 | ATGGTATCAAGCTTGCCACACCTGCGTGCAAATCTAACAA |
| ZAA185 | ATGGTATCAAGCTTGCCACAGCCTGCTGCAAACCATTAAA |
| ZAA186 | ATGGTATCAAGCTTGCCACAAATGATGTTGAATGTGCCCG |
| ZAA187 | ATGGTATCAAGCTTGCCACAGGTACAAGCAACGATCTCCA |
| ZAA188 | ATGGTATCAAGCTTGCCACAGCCAGACGGACAATGTTAGA |
| ZAA189 | ATGGTATCAAGCTTGCCACACCACGCATCCAAGATTAGGA |
| ZAA190 | ATGGTATCAAGCTTGCCACAGACTGCGAGCTATGTACCTT |
| ZAA191 | ATGGTATCAAGCTTGCCACAGCACATGAGAGACATTGAGC |
| ZAA192 | ATGGTATCAAGCTTGCCACAACAGCGTTATCACTTCTGCT |
| ZAA193 | ATGGTATCAAGCTTGCCACACTTGGTAATCGACGTTTCGG |
| ZAA194 | ATGGTATCAAGCTTGCCACAACCTTGACGAGATGATTCGG |
| ZAA195 | ATGGTATCAAGCTTGCCACATGACTATGCAGATACGCTGG |
| ZAA196 | ATGGTATCAAGCTTGCCACACTCATCTACAGGGAGCGAAG |
| ZAA197 | ATGGTATCAAGCTTGCCACACGCCCAATGCAATTTGTAGA |
| ZAA198 | ATGGTATCAAGCTTGCCACATACTGCGAGATACGTGTCTG |
| ZAA199 | ATGGTATCAAGCTTGCCACACTATGACGGAGCAGATACCG |
| ZAA200 | ATGGTATCAAGCTTGCCACAACTAAGCTGTCGGAATACCG |
| ZAA201 | ATGGTATCAAGCTTGCCACAATCCGTACTGACATTGACCG |
| ZAA202 | ATGGTATCAAGCTTGCCACAAGTAACACCGTATAGCACCG |
| ZAA203 | ATGGTATCAAGCTTGCCACAACATACGAGCTTAGGAACCG |
| ZAA204 | ATGGTATCAAGCTTGCCACAGACGATAGCCTACCAGTACG |
| ZAA205 | ATGGTATCAAGCTTGCCACATACTCAGCACGACTATGACG |
| ZAA206 | ATGGTATCAAGCTTGCCACAATAACTGGGTCGAACGGATC |
| ZAA207 | ATGGTATCAAGCTTGCCACACACAGACCGACAGTAGGATC |
| ZAA208 | ATGGTATCAAGCTTGCCACATACACCGACGTAACGAGATC |
| ZAA209 | ATGGTATCAAGCTTGCCACAGAGCTGATGTCACTCTTGGT |
| ZAA210 | ATGGTATCAAGCTTGCCACAGGTGCTCAGTCATCTTCACT |
| ZAA211 | ATGGTATCAAGCTTGCCACACGAGATAAGGTGCGATCACT |
| ZAA212 | ATGGTATCAAGCTTGCCACATCATAGTACGCATGTCGAGG |
| ZAA213 | ATGGTATCAAGCTTGCCACACGATACTCCAGCAGTCAGAG |
| ZAA214 | ATGGTATCAAGCTTGCCACAAACCACCGAGTACAGTTCAG |
| ZAA215 | ATGGTATCAAGCTTGCCACAACGACCGTCTAACTATGCAG |
| ZAA216 | ATGGTATCAAGCTTGCCACATATTCGACCATGCCCAGAAG |
| ZAA217 | ATGGTATCAAGCTTGCCACAACGACACCGAGAGCTTATTC |
| ZAA218 | ATGGTATCAAGCTTGCCACAGCAAGGCAGCGTAACTATTC |
| ZAA219 | ATGGTATCAAGCTTGCCACAACGCACACTGTTTAGAATGC |
| ZAA220 | ATGGTATCAAGCTTGCCACAACCAACAGGTTTGACAATGC |
| ZAA221 | ATGGTATCAAGCTTGCCACAATAACCATTTCGCAGAACGC |
| ZAA222 | ATGGTATCAAGCTTGCCACATCAATCTAGCGGAAGTGAGC |
| ZAA223 | ATGGTATCAAGCTTGCCACAACACGTAATTCTTCGCAAGC |
| ZAA224 | ATGGTATCAAGCTTGCCACAACTGAATAGACATGACGGCC |
| ZAA225 | ATGGTATCAAGCTTGCCACAAACACTAGAGGAACTTCGCC |
| ZAA226 | ATGGTATCAAGCTTGCCACAGAACTTTGTCCAAGATCGCC |
| ZAA227 | ATGGTATCAAGCTTGCCACAACCTAACTCGAAGTGAAGCC |
| ZAA228 | ATGGTATCAAGCTTGCCACAACGATAATCACAGATGGCCC |
| ZAA229 | ATGGTATCAAGCTTGCCACATGGCACAGACTGAACACTAC |
| ZAA230 | ATGGTATCAAGCTTGCCACATGGCGAGTAATACAGACGAC |
| ZAA231 | ATGGTATCAAGCTTGCCACAGCGCCGCTAATGAATTGTTA |
| ZAA232 | ATGGTATCAAGCTTGCCACATTCAGACGAAGCAAGTGTGA |
| ZAA233 | ATGGTATCAAGCTTGCCACATCATCAACAGCAACTCGTGA |
| ZAA234 | ATGGTATCAAGCTTGCCACAGCTCAGAGAACACTACGTGA |
| ZAA235 | ATGGTATCAAGCTTGCCACAAAGCGTTGACAAGCTAGTGA |
| ZAA236 | ATGGTATCAAGCTTGCCACAAGCCCAACTCGTGTAAATGA |
| ZAA237 | ATGGTATCAAGCTTGCCACATCCTAGCATACAACGAGGGA |
| ZAA238 | ATGGTATCAAGCTTGCCACAGCCCAAGACTTCTAAACGGA |
| ZAA239 | ATGGTATCAAGCTTGCCACAATACGTGATTAACGGGTCCG |
| ZAA240 | ATGGTATCAAGCTTGCCACAACGGAAACCACAGTCCTTAG |
| ZAA241 | ATGGTATCAAGCTTGCCACAAAACCGTGTGGAAGACCTAG |
| ZAA242 | ATGGTATCAAGCTTGCCACAGGTAACACCCAACTTAGCGA |
| ZAA243 | ATGGTATCAAGCTTGCCACACACCAGGTTGTCGAAACTTC |
| ZAA244 | ATGGTATCAAGCTTGCCACAACGCTACGCTTATCGAGTTT |
| ZAA245 | ATGGTATCAAGCTTGCCACAACTCCCGGTATCGAGCTATT |
| ZAA246 | ATGGTATCAAGCTTGCCACAAGCGACGAGTTCCGATAATT |
| ZAA247 | ATGGTATCAAGCTTGCCACAAACGAGTTGTGACACTTGGT |
| ZAA248 | ATGGTATCAAGCTTGCCACAACCCTCAGACTACGTGTTTG |
| ZAA249 | ATGGTATCAAGCTTGCCACAAGATTACCGTCCACTGGTTG |
| ZAA250 | ATGGTATCAAGCTTGCCACAAAATTGGTCGTAATGCGTCG |
| ZAA251 | ATGGTATCAAGCTTGCCACAAATTACTTTGAATGGGCGCG |
| ZAA252 | ATGGTATCAAGCTTGCCACAATTTAGCGACGAGGATACCG |
| ZAA253 | ATGGTATCAAGCTTGCCACAAATCTCTTAGAACGGGCACC |
| ZAA254 | ATGGTATCAAGCTTGCCACAAACTGAACATACCCGGTGAC |
| ZAA255 | ATGGTATCAAGCTTGCCACAACAGTGCTTAAAGGTTCGGA |
| ZAA256 | ATGGTATCAAGCTTGCCACAACAGTGGTCAAACGTGGTAA |
| ZAA257 | ATGGTATCAAGCTTGCCACATATAGCCGTCTAGCCGACTT |
| ZAA258 | ATGGTATCAAGCTTGCCACAGCATGTCAGTAACGGGTTTC |
| ZAA259 | ATGGTATCAAGCTTGCCACAGAACAACCAAGACGTGTGTC |
| ZAA260 | ATGGTATCAAGCTTGCCACAGAAGCACCTTTCACGAAGTC |
| ZAA261 | ATGGTATCAAGCTTGCCACAGGATGTCACCAACTAACCGA |
| ZAA262 | ATGGTATCAAGCTTGCCACAGCGCGGTTTCAATAAAGACA |
| ZAA263 | ATGGTATCAAGCTTGCCACAGGCAATTCCAAATCGTCCAA |
| ZAA264 | ATGGTATCAAGCTTGCCACACATATTGCTTAGAGGGCCGT |
| ZAA265 | ATGGTATCAAGCTTGCCACACCTAGTTCGAGATTGCGAGT |
| ZAA266 | ATGGTATCAAGCTTGCCACACCGAGTACGCCTCATTGTAT |
| ZAA267 | ATGGTATCAAGCTTGCCACACTACTACGATGGCGTCGAAT |
| ZAA268 | ATGGTATCAAGCTTGCCACACCGTGATTGACACTACGTTG |
| ZAA269 | ATGGTATCAAGCTTGCCACACATTAAGACGACTCGAACGC |
| ZAA270 | ATGGTATCAAGCTTGCCACACCCAAGAAGTGTTAGCAAGC |
| ZAA271 | ATGGTATCAAGCTTGCCACATATGACTTCCCATTCGGTCG |
| ZAA272 | ATGGTATCAAGCTTGCCACATGCTCTACCAAAGCCGTAAA |
| ZAA273 | ATGGTATCAAGCTTGCCACAGAGCCGTCTATTCACCTGTT |
| ZAA274 | ATGGTATCAAGCTTGCCACACGGCGAAGGAACCATGTATA |
| ZAA275 | ATGGTATCAAGCTTGCCACACCGGAAGCGCAAGTAGTATA |
| ZAA276 | ATGGTATCAAGCTTGCCACACCAGTACGCCCAATGGAATA |
| ZAA277 | ATGGTATCAAGCTTGCCACACAGAGTCTTAAACGCCTGGA |
| ZAA278 | ATGGTATCAAGCTTGCCACACCTGGTTGACAAGAACGAGA |
| ZAA279 | ATGGTATCAAGCTTGCCACAAACTGGTTCGCATGTATCGT |
| ZAA280 | ATGGTATCAAGCTTGCCACAAGGCTCAGCGTACCTAGTAT |
| ZAA281 | ATGGTATCAAGCTTGCCACAAGCCTAGACTTGTTCGCAAT |
| ZAA282 | ATGGTATCAAGCTTGCCACAAAGAGTGTCTAAGTCTCGCG |
| ZAA283 | ATGGTATCAAGCTTGCCACAATACGTTAGACAGGTCAGCG |
| ZAA284 | ATGGTATCAAGCTTGCCACAACTAAGTAGCCATGACGACG |
| ZAA285 | ATGGTATCAAGCTTGCCACAAGACCTCAGCTTCAGTAACG |
| ZAA286 | ATGGTATCAAGCTTGCCACAACATGATAACCCGTACTGGC |
| ZAA287 | ATGGTATCAAGCTTGCCACAAATACGACTGAAGGATCGGC |
| ZAA288 | ATGGTATCAAGCTTGCCACAAACTACTATGAAGCGGGAGC |
| ZAA289 | ATGGTATCAAGCTTGCCACAAAGATTTGCCAACGCTCCTA |
| ZAA290 | ATGGTATCAAGCTTGCCACAAACGCAGGCCAATGGTTATA |
| ZAA291 | ATGGTATCAAGCTTGCCACAACATGGTCTAAACGCTTCGA |
| ZAA292 | ATGGTATCAAGCTTGCCACAAAGTCTGTCAAACCTTCGCA |
| ZAA293 | ATGGTATCAAGCTTGCCACATATCTCGCGGGATCTAGTGT |
| ZAA294 | ATGGTATCAAGCTTGCCACATCAGGATGTCTCAGTACGGT |
| ZAA295 | ATGGTATCAAGCTTGCCACATCATCGGGTACACGTATGTG |
| ZAA296 | ATGGTATCAAGCTTGCCACATACCTTGAGCGAGTAAGCTG |
| ZAA297 | ATGGTATCAAGCTTGCCACATAAGCATAGCGACCTACACG |
| ZAA298 | ATGGTATCAAGCTTGCCACATACATCACCGTAAGTGGCAG |
| ZAA299 | ATGGTATCAAGCTTGCCACATAGCACCGACTACAGTGTTC |
| ZAA300 | ATGGTATCAAGCTTGCCACATACAGGGTCATAAGCGTGTC |
| ZAA301 | ATGGTATCAAGCTTGCCACATAATCGCTGGGAATACTGGC |
| ZAA302 | ATGGTATCAAGCTTGCCACATCGCGCTATAAACGAATCCA |
| ZAA303 | ATGGTATCAAGCTTGCCACAGACTGTGCTAGATCCGTGTT |
| ZAA304 | ATGGTATCAAGCTTGCCACAGCAACCTACTCTGAGCTGTT |
| ZAA305 | ATGGTATCAAGCTTGCCACAGATGTCGTCCCAGACTACTG |
| ZAA306 | ATGGTATCAAGCTTGCCACAGATCGTTACCCACTTGCATG |
| ZAA307 | ATGGTATCAAGCTTGCCACAGAGTCCTGACCACTATGTCG |
| ZAA308 | ATGGTATCAAGCTTGCCACAGTCACACAGCTAAGTAACGC |
| ZAA309 | ATGGTATCAAGCTTGCCACAGCTATGGACACATTTACGCC |
| ZAA310 | ATGGTATCAAGCTTGCCACAGAGAGACTATCAAGCCCGAC |
| ZAA311 | ATGGTATCAAGCTTGCCACAGCACATGACTCAAGTGGAAC |
| ZAA312 | ATGGTATCAAGCTTGCCACAGCCTAACGAGCAAGTCCATA |
| ZAA313 | ATGGTATCAAGCTTGCCACAATCTACGGAGTGAGACAGCT |
| ZAA314 | ATGGTATCAAGCTTGCCACAGAGTATCGCACATTCGCATC |
| ZAA315 | ATGGTATCAAGCTTGCCACAGCGTCTGACATAAGAGCAAC |
| ZAA316 | ATGGTATCAAGCTTGCCACAAATTCAGTAGTGAGGCGCAT |
| ZAA317 | ATGGTATCAAGCTTGCCACAGATGCTGACACATGATGCTC |
| ZAA318 | ATGGTATCAAGCTTGCCACAATGATAGGCTGCGACATTGT |
| ZAA319 | ATGGTATCAAGCTTGCCACAAAGACGCACTAAGCATTTGC |
| ZAA320 | ATGGTATCAAGCTTGCCACAGATCGGCATCTAGCTTGACT |
| ZAA321 | ATGGTATCAAGCTTGCCACAATTCAGCTCAGTCTGCACAT |
| ZAA322 | ATGGTATCAAGCTTGCCACAGCAAGAATACTGCTCAAGCC |
| ZAA323 | ATGGTATCAAGCTTGCCACACATCGAAGTTACTGCAAGCC |
| ZAA324 | ATGGTATCAAGCTTGCCACATAGCTCGATTGAACTGCCTC |
| ZAA325 | ATGGTATCAAGCTTGCCACAGCGCACAGCTCATTACATAG |
| ZAA326 | ATGGTATCAAGCTTGCCACAAATGTGCTACAAGAGCGTGA |
| ZAA327 | ATGGTATCAAGCTTGCCACAGCGCATCACATAAATCGTCA |
| ZAA328 | ATGGTATCAAGCTTGCCACATTCAGGCGTGCATGATGTAT |
| ZAA329 | ATGGTATCAAGCTTGCCACAACGCAGCTATGCTCATGTAT |
| ZAA330 | ATGGTATCAAGCTTGCCACAGCCGCATCTGTATCCATTTG |
| ZAA331 | ATGGTATCAAGCTTGCCACACATATCAGCGAATGCAGACC |
| ZAA332 | ATGGTATCAAGCTTGCCACAACACTCATGCAAGCGATGTA |
| ZAA333 | ATGGTATCAAGCTTGCCACATCTGACAGCAAATACGCTCA |
| ZAA334 | ATGGTATCAAGCTTGCCACAACAGCGTTGAAAGTAGCTCA |
| ZAA335 | ATGGTATCAAGCTTGCCACAGTCATCATCCTCATGCGTTG |
| ZAA336 | ATGGTATCAAGCTTGCCACATATCATCTGGGACAGCGTTG |
| ZAA337 | ATGGTATCAAGCTTGCCACAAATGACGTACAATCCGCTGA |
| ZAA338 | ATGGTATCAAGCTTGCCACAATGAACAGTGCATGAAGTGC |
| ZAA339 | ATGGTATCAAGCTTGCCACAGCTACGGACAAAGATCCTCA |
| ZAA340 | ATGGTATCAAGCTTGCCACAGGCGACTAGAAATCTCCACA |
| ZAA341 | ATGGTATCAAGCTTGCCACATCACATATAGCAGGTGCGAG |
| ZAA342 | ATGGTATCAAGCTTGCCACAATCACATAGAGAGACGCTGC |
| ZAA343 | ATGGTATCAAGCTTGCCACATAGAGCCTCAGCATTCATCG |
| ZAA344 | ATGGTATCAAGCTTGCCACAGCTACACACACAGAGTTGGA |
| ZAA345 | ATGGTATCAAGCTTGCCACATCAGTCGTAGCAGTTCGATG |
| ZAA346 | ATGGTATCAAGCTTGCCACAGCAGGATCAACACGAGTCTA |
| ZAA347 | ATGGTATCAAGCTTGCCACAGCTGAATGACCATGAAGAGC |
| ZAA348 | ATGGTATCAAGCTTGCCACATGTCACAGCACTAACAGGAC |
| ZAA349 | ATGGTATCAAGCTTGCCACAGCAGGCAGAACAGTCATCTA |
| ZAA350 | ATGGTATCAAGCTTGCCACACGATCAGTACCAACGATGGA |
| ZAA351 | ATGGTATCAAGCTTGCCACAAAGAGAGCGCAATTCTGCTA |
| ZAA352 | ATGGTATCAAGCTTGCCACAAATCGCTGACAATTCTGGGA |
| ZAA353 | ATGGTATCAAGCTTGCCACAACTATGCTGAAAGTGTGGCA |
| ZAA354 | ATGGTATCAAGCTTGCCACAGCGCTTATTAAATGTGCGGA |
| ZAA355 | ATGGTATCAAGCTTGCCACACTCCATACAACAGAGTGGCA |
| ZAA356 | ATGGTATCAAGCTTGCCACATAGTTAGCCGCACTCTCATG |
| ZAA357 | ATGGTATCAAGCTTGCCACAAGATGCACCCAATTCTTCGA |
| ZAA358 | ATGGTATCAAGCTTGCCACAGGCAAGATGCTCTAACATGC |
| ZAA359 | ATGGTATCAAGCTTGCCACACTAAGTGCTGTGAATACGCG |
| ZAA360 | ATGGTATCAAGCTTGCCACACATCTGATAGCATTGCGGTG |
| ZAA361 | ATGGTATCAAGCTTGCCACAGAGTCATGCACATTTCGGTC |
| ZAA362 | ATGGTATCAAGCTTGCCACAAAAGCGCAGTGCTCATAATG |
| ZAA363 | ATGGTATCAAGCTTGCCACAACACGCAGATGATTTAAGCG |
| ZAA364 | ATGGTATCAAGCTTGCCACAATGATACTAGCATCGAGGCG |
| ZAA365 | ATGGTATCAAGCTTGCCACAAAACACTTAAACCCGTTCGG |
| ZAA366 | ATGGTATCAAGCTTGCCACAACGACTATAAATGACCGCCG |
| ZAA367 | ATGGTATCAAGCTTGCCACAATAGTAAGACACCAGCCGCG |
| ZAA368 | ATGGTATCAAGCTTGCCACACGCATGTTAATCATTGACCC |
| ZAA369 | ATGGTATCAAGCTTGCCACACGCCAGAGACGCAGATAGTA |
| ZAA370 | ATGGTATCAAGCTTGCCACACGCCATTGAAGTCGAGATAA |
| ZAA371 | ATGGTATCAAGCTTGCCACAGAATTGAATATCTCGTCCCG |
| ZAA372 | ATGGTATCAAGCTTGCCACAGAGCGTGAATGAGCCTTAAC |
| ZAA373 | ATGGTATCAAGCTTGCCACAGCTCCCGACCGTAGTGTATA |
| ZAA374 | ATGGTATCAAGCTTGCCACAGTGTCACCCTCGCGTATAAG |
| ZAA375 | ATGGTATCAAGCTTGCCACAAAATAACGAACCGCCTCTGG |
| ZAA376 | ATGGTATCAAGCTTGCCACAAACGAGTTAAACGGATCGCC |
| ZAA377 | ATGGTATCAAGCTTGCCACAAACTTTGCACAACGAACGCG |
| ZAA378 | ATGGTATCAAGCTTGCCACAAAGTCCGCAAACGTACAGCG |
| ZAA379 | ATGGTATCAAGCTTGCCACAACCGCAAGAAATATCCTCGG |
| ZAA380 | ATGGTATCAAGCTTGCCACACATCAATTAAGCAGCGAGCC |
| ZAA381 | ATGGTATCAAGCTTGCCACAGCCCGCGTAAGTTCGTCTAA |
| ZAA382 | ATGGTATCAAGCTTGCCACAGCGTGCCCACCTATTGGTAT |
| ZAA383 | ATGGTATCAAGCTTGCCACATAATTGAGCTGACGGCGCAC |
| ZAA384 | ATGGTATCAAGCTTGCCACATTCTTCTTAGGCGCTCGACG |
| ZBB001 | GTCGAGCTCTCTACTGCATAGATTAGCGTACATAGGCCCG |
| ZBB002 | GTCGAGCTCTCTACTGCATACCAAGATCGGCTAAGTCGAG |
| ZBB003 | GTCGAGCTCTCTACTGCATAATATTGGACGAATCGCCACC |
| ZBB004 | GTCGAGCTCTCTACTGCATAAAATGCTAGTCAAGCGGACC |
| ZBB005 | GTCGAGCTCTCTACTGCATAGGCGGACTACAACACATTCA |
| ZBB006 | GTCGAGCTCTCTACTGCATAAGATACAGGACATTCAGCGC |
| ZBB007 | GTCGAGCTCTCTACTGCATACATCAAAGAACACGCTGTCG |
| ZBB008 | GTCGAGCTCTCTACTGCATACAGACTCAGGTAGCATGTCG |
| ZBB009 | GTCGAGCTCTCTACTGCATATAATCACGTCGAACCTGAGC |
| ZBB010 | GTCGAGCTCTCTACTGCATAAACGACTACGGCACATATCG |
| ZBB011 | GTCGAGCTCTCTACTGCATAACGCATGACCCATTCGATAG |
| ZBB012 | GTCGAGCTCTCTACTGCATATGATCCTGAACACGATTGCC |
| ZBB013 | GTCGAGCTCTCTACTGCATAGGACTTCCACGACGTATCTG |
| ZBB014 | GTCGAGCTCTCTACTGCATAATTCTCGATAGAGCGTGCTG |
| ZBB015 | GTCGAGCTCTCTACTGCATAGAGATTTACACACATGCGCC |
| ZBB016 | GTCGAGCTCTCTACTGCATACATGATCTGAAAGACCGCCA |
| ZBB017 | GTCGAGCTCTCTACTGCATAAGTTCGACTGAATCGCCATC |
| ZBB018 | GTCGAGCTCTCTACTGCATACCACGGTCGAGACTGATATG |
| ZBB019 | GTCGAGCTCTCTACTGCATAGAGTCTCACCGACACTGATG |
| ZBB020 | GTCGAGCTCTCTACTGCATATCCATCGAGCTGGTTGAATG |
| ZBB021 | GTCGAGCTCTCTACTGCATATCAGTCCAGATTGCCGAATG |
| ZBB022 | GTCGAGCTCTCTACTGCATACAGATCCGAACACATCGTGA |
| ZBB023 | GTCGAGCTCTCTACTGCATAGACCGCGATATTTGGCAATG |
| ZBB024 | GTCGAGCTCTCTACTGCATACCTCGCGCTAGATAGATGTG |
| ZBB025 | GTCGAGCTCTCTACTGCATAAGTACACTCGCATAAGCGTC |
| ZBB026 | GTCGAGCTCTCTACTGCATATAAATCTCGCGCAAGCCTTA |
| ZBB027 | GTCGAGCTCTCTACTGCATACATACGTCACGACTAGCTGG |
| ZBB028 | GTCGAGCTCTCTACTGCATAAATACCGTTTAAGCCGTGGG |
| ZBB029 | GTCGAGCTCTCTACTGCATAGCAAACAATAGACGTTCGGG |
| ZBB030 | GTCGAGCTCTCTACTGCATAATACACATCGGGTTAAGCGG |
| ZBB031 | GTCGAGCTCTCTACTGCATAATAATCGGGCTTAACGACGG |
| ZBB032 | GTCGAGCTCTCTACTGCATAGCACACTGAGCCAACTGATA |
| ZBB033 | GTCGAGCTCTCTACTGCATAAGCCAAAGAAGACTGTGTCG |
| ZBB034 | GTCGAGCTCTCTACTGCATAGAAACAACTACAGCGTGTCG |
| ZBB035 | GTCGAGCTCTCTACTGCATATCGCAGTACGTTAAGTGTCG |
| ZBB036 | GTCGAGCTCTCTACTGCATATCTACTATCAGAGGGCGTCG |
| ZBB037 | GTCGAGCTCTCTACTGCATAACACAGGCGTTAATCTCTCG |
| ZBB038 | GTCGAGCTCTCTACTGCATATAAGAGTGCATGACAGCTCG |
| ZBB039 | GTCGAGCTCTCTACTGCATACATAGGTAGCGACACTAGCG |
| ZBB040 | GTCGAGCTCTCTACTGCATATAGTAACGTCGATGACAGCG |
| ZBB041 | GTCGAGCTCTCTACTGCATATCAGCTTTGGGAATATGCCG |
| ZBB042 | GTCGAGCTCTCTACTGCATAGCACTTGGTTCAATTCCGTC |
| ZBB043 | GTCGAGCTCTCTACTGCATAGCTTATTGAACATGCCCGTC |
| ZBB044 | GTCGAGCTCTCTACTGCATATACATGGAGATAACGAGCGC |
| ZBB045 | GTCGAGCTCTCTACTGCATACGCCTGATGAAAGACGTACA |
| ZBB046 | GTCGAGCTCTCTACTGCATACAATCTGTAGGCAGCGGAAT |
| ZBB047 | GTCGAGCTCTCTACTGCATACCAAACAACCGATTCGTGAG |
| ZBB048 | GTCGAGCTCTCTACTGCATAGAGTCTCGACCATTCAGCAG |
| ZBB049 | GTCGAGCTCTCTACTGCATAACGCGCTAAGCAGTAGTAAG |
| ZBB050 | GTCGAGCTCTCTACTGCATACGGCGTTTAGACGAACAAAG |
| ZBB051 | GTCGAGCTCTCTACTGCATAAGCTAACCGAGACGTTGTTC |
| ZBB052 | GTCGAGCTCTCTACTGCATACCAGGTTAGACATCGAGACG |
| ZBB053 | GTCGAGCTCTCTACTGCATATTTACCCAGCGGACTTAACG |
| ZBB054 | GTCGAGCTCTCTACTGCATAGAATCGCAGCTTGTCGAATC |
| ZBB055 | GTCGAGCTCTCTACTGCATAGACTTGCTAACATGCTTCGC |
| ZBB056 | GTCGAGCTCTCTACTGCATAAGATTCACACCATGAGTCGC |
| ZBB057 | GTCGAGCTCTCTACTGCATACATGAAATAACAGGTGGCGC |
| ZBB058 | GTCGAGCTCTCTACTGCATATGAGGATAAAGAACTCGCGC |
| ZBB059 | GTCGAGCTCTCTACTGCATAAGTATAAGCATAACGCCCGC |
| ZBB060 | GTCGAGCTCTCTACTGCATAAGTATCACACGAGCTTACGC |
| ZBB061 | GTCGAGCTCTCTACTGCATAGCTCAACAAACAGATGTGCC |
| ZBB062 | GTCGAGCTCTCTACTGCATAGAGCAGACAGACTCGATCAC |
| ZBB063 | GTCGAGCTCTCTACTGCATAGCTGAAGAAGTTACGGCAAC |
| ZBB064 | GTCGAGCTCTCTACTGCATATCCGCACATTCAAGCAAGTA |
| ZBB065 | GTCGAGCTCTCTACTGCATAGTCGCAACAGTGACCCAATA |
| ZBB066 | GTCGAGCTCTCTACTGCATATGCGGCCACATAAATTACCA |
| ZBB067 | GTCGAGCTCTCTACTGCATAGGGATTCGCCACAACATACA |
| ZBB068 | GTCGAGCTCTCTACTGCATAGTCCGGCCATGAAACTAACA |
| ZBB069 | GTCGAGCTCTCTACTGCATAAAACGGTCCTTCATTCGCTT |
| ZBB070 | GTCGAGCTCTCTACTGCATATAAGGGTTACGAACATCGCC |
| ZBB071 | GTCGAGCTCTCTACTGCATAGACCCTGACGTTTGCTAAGT |
| ZBB072 | GTCGAGCTCTCTACTGCATACGAGTCGGACGATACCCTAT |
| ZBB073 | GTCGAGCTCTCTACTGCATACCCGTAACAGATTGCGAAAC |
| ZBB074 | GTCGAGCTCTCTACTGCATACACGTCGTTAAAGACCTGGA |
| ZBB075 | GTCGAGCTCTCTACTGCATAACCGACCGATAATGTGGTTC |
| ZBB076 | GTCGAGCTCTCTACTGCATAACGTCAAACAAAGTCGTTGC |
| ZBB077 | GTCGAGCTCTCTACTGCATAAGGTACTTTCAACTGCGTCC |
| ZBB078 | GTCGAGCTCTCTACTGCATAGGTGCTCATTACGTTCCACT |
| ZBB079 | GTCGAGCTCTCTACTGCATAGTACGCGCAGTATCGGTAAT |
| ZBB080 | GTCGAGCTCTCTACTGCATAGCTTTCCGCAAACTGAACAA |
| ZBB081 | GTCGAGCTCTCTACTGCATACTGTACGTGCGATACTCGTT |
| ZBB082 | GTCGAGCTCTCTACTGCATACGGCTGACTATCGTTCCATT |
| ZBB083 | GTCGAGCTCTCTACTGCATACCCACGTATAGCGTACTGTG |
| ZBB084 | GTCGAGCTCTCTACTGCATACATCTACGGACACGGTATCG |
| ZBB085 | GTCGAGCTCTCTACTGCATACTTCGAGCACGGTACAGTAG |
| ZBB086 | GTCGAGCTCTCTACTGCATATCATTCCGTAGATGGGTCGT |
| ZBB087 | GTCGAGCTCTCTACTGCATATCGAGTACCGTAGATTCGCT |
| ZBB088 | GTCGAGCTCTCTACTGCATATTAGATACGCGACTCGTCCT |
| ZBB089 | GTCGAGCTCTCTACTGCATATCCGGTACGAGCAAAGTAGA |
| ZBB090 | GTCGAGCTCTCTACTGCATACGTGGCGATAATACGAAAGC |
| ZBB091 | GTCGAGCTCTCTACTGCATACCCTTGGATAAAGCGAAGCA |
| ZBB092 | GTCGAGCTCTCTACTGCATACCGGCAGCGAAACTATCTAA |
| ZBB093 | GTCGAGCTCTCTACTGCATACGGACTAGCAAACTGTCGAA |
| ZBB094 | GTCGAGCTCTCTACTGCATAACGACCGTTGCATTGATTCT |
| ZBB095 | GTCGAGCTCTCTACTGCATAAAAGGCATTGTCGCTAAGCT |
| ZBB096 | GTCGAGCTCTCTACTGCATAAACTGGTTAGCATCCGTGTG |
| ZBB097 | GTCGAGCTCTCTACTGCATAAATCGACGCTAACGACTGAG |
| ZBB098 | GTCGAGCTCTCTACTGCATAAATCAGCCGTAACGTGAGAG |
| ZBB099 | GTCGAGCTCTCTACTGCATAAATGTAACCGCATGGGTCAG |
| ZBB100 | GTCGAGCTCTCTACTGCATAAATGCGACGTAACTTTGCAG |
| ZBB101 | GTCGAGCTCTCTACTGCATAATTACATACGCGCTGGGAAG |
| ZBB102 | GTCGAGCTCTCTACTGCATAATTGAAGCGGTCGGAATCTC |
| ZBB103 | GTCGAGCTCTCTACTGCATAAGATACGTCGAACATTGCCC |
| ZBB104 | GTCGAGCTCTCTACTGCATAAACAGTCTCCAAGAGGCGTA |
| ZBB105 | GTCGAGCTCTCTACTGCATATACTTAGGCGCATCAGTTGG |
| ZBB106 | GTCGAGCTCTCTACTGCATATTACATAGTACACTGCGCGG |
| ZBB107 | GTCGAGCTCTCTACTGCATATCACAGCCTTTGAATAGCGG |
| ZBB108 | GTCGAGCTCTCTACTGCATATATTGTCGAACATGGGTGCC |
| ZBB109 | GTCGAGCTCTCTACTGCATATAATTCCAGCGAAGTCTGCC |
| ZBB110 | GTCGAGCTCTCTACTGCATATTTGGACAGACAGCGAGAAC |
| ZBB111 | GTCGAGCTCTCTACTGCATAGACCTAAGCGCAGAGACTTC |
| ZBB112 | GTCGAGCTCTCTACTGCATAGCGCCGCTTCAATTTCATTA |
| ZBB113 | GTCGAGCTCTCTACTGCATACTATTGTAGAGACGCGGCAT |
| ZBB114 | GTCGAGCTCTCTACTGCATAGATCATGCTACATTCGCACG |
| ZBB115 | GTCGAGCTCTCTACTGCATAGCATTCACGATAACGGCATC |
| ZBB116 | GTCGAGCTCTCTACTGCATAGTGCGACACATGACATCAAC |
| ZBB117 | GTCGAGCTCTCTACTGCATACAAGACGCTGTTGCATTTGT |
| ZBB118 | GTCGAGCTCTCTACTGCATACAGTGGGCAACATCGTCTTA |
| ZBB119 | GTCGAGCTCTCTACTGCATATGCATAACACGACACGTTCA |
| ZBB120 | GTCGAGCTCTCTACTGCATAGCAGCACTTACAGCTTTGTC |
| ZBB121 | GTCGAGCTCTCTACTGCATAACACGGCATCAATGTGCTTA |
| ZBB122 | GTCGAGCTCTCTACTGCATATCAGTATTAGCATGTGCCGG |
| ZBB123 | GTCGAGCTCTCTACTGCATATATGAGACGCCATGCACTTC |
| ZBB124 | GTCGAGCTCTCTACTGCATAATACTATGAGCATCGTGCGG |
| ZBB125 | GTCGAGCTCTCTACTGCATAACGAGGCACGCATTATGTAG |
| ZBB126 | GTCGAGCTCTCTACTGCATAGATAGCATCCGAGACAGCTC |
| ZBB127 | GTCGAGCTCTCTACTGCATAGAGAGCCCGACAACATTTCA |
| ZBB128 | GTCGAGCTCTCTACTGCATAGACATAGTCACAGAGTCCGC |
| ZBB129 | GTCGAGCTCTCTACTGCATAACATTTGGCACAAGTGGTCA |
| ZBB130 | GTCGAGCTCTCTACTGCATACGCACAGAGCTAATCATTGC |
| ZBB131 | GTCGAGCTCTCTACTGCATAGCATCCTCGCAATGTCTGTA |
| ZBB132 | GTCGAGCTCTCTACTGCATAGAGAATCGTCCACAACGTCA |
| ZBB133 | GTCGAGCTCTCTACTGCATAGCCCGCTGTAAACATAGACA |
| ZBB134 | GTCGAGCTCTCTACTGCATAGCAGCAGCTAAACTCAGTCA |
| ZBB135 | GTCGAGCTCTCTACTGCATAGACTGACGTGCATTCTGTTG |
| ZBB136 | GTCGAGCTCTCTACTGCATATACATAGGCATAAGGCGTGC |
| ZBB137 | GTCGAGCTCTCTACTGCATACAGCTTACGCAATCTGGGTA |
| ZBB138 | GTCGAGCTCTCTACTGCATAATTCACCTGCGAGATGGTTG |
| ZBB139 | GTCGAGCTCTCTACTGCATATCAATGGTGAGCAATGCGTA |
| ZBB140 | GTCGAGCTCTCTACTGCATAAATCGACTGGAAGTGTCTGC |
| ZBB141 | GTCGAGCTCTCTACTGCATACGACATCAGAGACCAGTAGC |
| ZBB142 | GTCGAGCTCTCTACTGCATAAGAAGACGCTCTCAATCTGC |
| ZBB143 | GTCGAGCTCTCTACTGCATACCAGGTATGAAACGCGATCA |
| ZBB144 | GTCGAGCTCTCTACTGCATATTGCGAAGAACAGAGTTGCA |
| ZBB145 | GTCGAGCTCTCTACTGCATAGATCGTCATCCACGCTTGAT |
| ZBB146 | GTCGAGCTCTCTACTGCATAACACGGTATGAACTGACTGC |
| ZBB147 | GTCGAGCTCTCTACTGCATAAATCTAGCTGAAGCGACTGC |
| ZBB148 | GTCGAGCTCTCTACTGCATACCGAGTGACACAGATAGAGC |
| ZBB149 | GTCGAGCTCTCTACTGCATATACACTGCAAGAGAGCTGGA |
| ZBB150 | GTCGAGCTCTCTACTGCATACAGATGTGCAAACGCTTGAA |
| ZBB151 | GTCGAGCTCTCTACTGCATAACTCATCTGGCATGTGTGTG |
| ZBB152 | GTCGAGCTCTCTACTGCATACCGTCTCAGAAAGCATGGAA |
| ZBB153 | GTCGAGCTCTCTACTGCATACACAGAGCGTACTCCAGATG |
| ZBB154 | GTCGAGCTCTCTACTGCATATATCAGTCCACACGTATGCG |
| ZBB155 | GTCGAGCTCTCTACTGCATACAATGCGGTACAAGCTCCTA |
| ZBB156 | GTCGAGCTCTCTACTGCATAATATCTTCGAGAGGCGCATG |
| ZBB157 | GTCGAGCTCTCTACTGCATATATGCAGACACTGAACCAGC |
| ZBB158 | GTCGAGCTCTCTACTGCATATGGCAGACACTAATCACAGC |
| ZBB159 | GTCGAGCTCTCTACTGCATACATGCAACGAACCTAGTCGA |
| ZBB160 | GTCGAGCTCTCTACTGCATAGCACAGGCTTAATTGCACTC |
| ZBB161 | GTCGAGCTCTCTACTGCATAGAACTGAATGCACTCGAAGC |
| ZBB162 | GTCGAGCTCTCTACTGCATAACACTGCGTAAATCTTGCGA |
| ZBB163 | GTCGAGCTCTCTACTGCATACATCGCTATGGATCGGTGTT |
| ZBB164 | GTCGAGCTCTCTACTGCATACAGATGCTTCGACTCCTGTT |
| ZBB165 | GTCGAGCTCTCTACTGCATAACTAGAGCTGCCTTAATGCG |
| ZBB166 | GTCGAGCTCTCTACTGCATATATATGCGAGCACAGGATGC |
| ZBB167 | GTCGAGCTCTCTACTGCATAAGACGATCACCATTCGATGC |
| ZBB168 | GTCGAGCTCTCTACTGCATAAATCACTCCTAATGCTGGCG |
| ZBB169 | GTCGAGCTCTCTACTGCATAGTAGCTCATCCACTATGGCG |
| ZBB170 | GTCGAGCTCTCTACTGCATAATCGCAACTAACGCGCTATA |
| ZBB171 | GTCGAGCTCTCTACTGCATACATATAGGAGCAGTACGCCG |
| ZBB172 | GTCGAGCTCTCTACTGCATATAATGGACATGACGCGACAG |
| ZBB173 | GTCGAGCTCTCTACTGCATAGTGCAGACTCAACACCATGA |
| ZBB174 | GTCGAGCTCTCTACTGCATACATTTGGCAAGAGCTGTGAC |
| ZBB175 | GTCGAGCTCTCTACTGCATACGCCCGACATGATGAAAGTA |
| ZBB176 | GTCGAGCTCTCTACTGCATAGCGCAATGAACAGCTTGTAA |
| ZBB177 | GTCGAGCTCTCTACTGCATACAGTCTGCGATACGCTAGTT |
| ZBB178 | GTCGAGCTCTCTACTGCATATATCTGGTACGAGTGCTGCT |
| ZBB179 | GTCGAGCTCTCTACTGCATAACCATGACTGTCCACGAATG |
| ZBB180 | GTCGAGCTCTCTACTGCATATTCATCAGAGGCTGTAAGCG |
| ZBB181 | GTCGAGCTCTCTACTGCATACTTTCAGATACGACCGGGTG |
| ZBB182 | GTCGAGCTCTCTACTGCATACGAAGCTCGTTTAATCGGTG |
| ZBB183 | GTCGAGCTCTCTACTGCATAGACACTCTCGAACTGTAGGC |
| ZBB184 | GTCGAGCTCTCTACTGCATACTGACTCACGGACGATAGTG |
| ZBB185 | GTCGAGCTCTCTACTGCATAACAGTGCAGTCATACGAGTG |
| ZBB186 | GTCGAGCTCTCTACTGCATACAGACCGACTTAGACGAGTG |
| ZBB187 | GTCGAGCTCTCTACTGCATAAGCACTCTTCACAGATGACG |
| ZBB188 | GTCGAGCTCTCTACTGCATAACAAGCGGATCTAATCGCAG |
| ZBB189 | GTCGAGCTCTCTACTGCATATTAATCGTGGTGCAGCAGTT |
| ZBB190 | GTCGAGCTCTCTACTGCATAACATTGGTTAAAGGCGCTGA |
| ZBB191 | GTCGAGCTCTCTACTGCATAAATCACGCCCAATACGTTGA |
| ZBB192 | GTCGAGCTCTCTACTGCATAGGCTACACGAAATCGCTACA |
| ZBB193 | GTCGAGCTCTCTACTGCATATACTAAGAGGCATGAAGCGC |
| ZBB194 | GTCGAGCTCTCTACTGCATAGCGATCTCGAAAGCCATACA |
| ZBB195 | GTCGAGCTCTCTACTGCATAATGCACGACTTCTGAGCAAT |
| ZBB196 | GTCGAGCTCTCTACTGCATAAGCTCGATCACAGCGTATTC |
| ZBB197 | GTCGAGCTCTCTACTGCATACGACGTTCAGCATACTAGGG |
| ZBB198 | GTCGAGCTCTCTACTGCATATATCACGTCGCATGTAGTGG |
| ZBB199 | GTCGAGCTCTCTACTGCATAAACCAGAGTTTCTAACCGCG |
| ZBB200 | GTCGAGCTCTCTACTGCATACTTGCAGACGAACAGATGGA |
| ZBB201 | GTCGAGCTCTCTACTGCATAGCACTTGACAGATGCTTGTG |
| ZBB202 | GTCGAGCTCTCTACTGCATATCGACGAAACAAGTCTAGCC |
| ZBB203 | GTCGAGCTCTCTACTGCATAAGACGTATAACATGCTGCCC |
| ZBB204 | GTCGAGCTCTCTACTGCATACATTATGCACGATGGATGCG |
| ZBB205 | GTCGAGCTCTCTACTGCATAGACTCTACGATACGTCGCTG |
| ZBB206 | GTCGAGCTCTCTACTGCATAGACTAGGACCGATTGCACTG |
| ZBB207 | GTCGAGCTCTCTACTGCATATATAATCTTGGCGAACGGGC |
| ZBB208 | GTCGAGCTCTCTACTGCATACAGCTAGTATCAGCGACCTG |
| ZBB209 | GTCGAGCTCTCTACTGCATAGCTACAAACATAAGTGCGGC |
| ZBB210 | GTCGAGCTCTCTACTGCATAGTCCGACGTTAGATCACCTG |
| ZBB211 | GTCGAGCTCTCTACTGCATAGCCGTAGCGACAATCTAAGA |
| ZBB212 | GTCGAGCTCTCTACTGCATACCGAAGCACAGACAGTGTTA |
| ZBB213 | GTCGAGCTCTCTACTGCATATGAGACGCTCTATATCGCCT |
| ZBB214 | GTCGAGCTCTCTACTGCATATACTGCTAACCATGCTTGGC |
| ZBB215 | GTCGAGCTCTCTACTGCATAGCCACGACTCAACAGTGATA |
| ZBB216 | GTCGAGCTCTCTACTGCATATATTGAGCCATACGCCACAG |
| ZBB217 | GTCGAGCTCTCTACTGCATACATGGACGGATGCCGATATT |
| ZBB218 | GTCGAGCTCTCTACTGCATACACGGCAGGCTCTTCATATT |
| ZBB219 | GTCGAGCTCTCTACTGCATACGCCATAGAGAAGTGACCAC |
| ZBB220 | GTCGAGCTCTCTACTGCATACCTGTAGACAAATGACGGCA |
| ZBB221 | GTCGAGCTCTCTACTGCATATGTGTGTCAACAACCACCAA |
| ZBB222 | GTCGAGCTCTCTACTGCATAGCCATCGGTGTCATGTACTT |
| ZBB223 | GTCGAGCTCTCTACTGCATAATTCTAATGCCACTTGCGCT |
| ZBB224 | GTCGAGCTCTCTACTGCATAAATCGTCACCAACGTGCATA |
| ZBB225 | GTCGAGCTCTCTACTGCATAAAATAACCCGCAGTAGTCGG |
| ZBB226 | GTCGAGCTCTCTACTGCATATCTAGGGTACTACGCATCGG |
| ZBB227 | GTCGAGCTCTCTACTGCATAACACGATACTCAGCATGACG |
| ZBB228 | GTCGAGCTCTCTACTGCATAAAGTCACTGGAATGCCGATC |
| ZBB229 | GTCGAGCTCTCTACTGCATAACTAGATACACATGCGTGCC |
| ZBB230 | GTCGAGCTCTCTACTGCATAGCCATCTAGCAACAGCGATA |
| ZBB231 | GTCGAGCTCTCTACTGCATATCAGTCGGTCTAATGTTGCG |
| ZBB232 | GTCGAGCTCTCTACTGCATACAGCGAGTGACATTTATGCG |
| ZBB233 | GTCGAGCTCTCTACTGCATATAGCGATTACCAGTCATGCG |
| ZBB234 | GTCGAGCTCTCTACTGCATACTAGCAGGTAGCACGATACG |
| ZBB235 | GTCGAGCTCTCTACTGCATACACGACCGACAATATGGTGA |
| ZBB236 | GTCGAGCTCTCTACTGCATAATTATCTACAGCCAGCGCAG |
| ZBB237 | GTCGAGCTCTCTACTGCATACCATCGTAACGGGACTGAAG |
| ZBB238 | GTCGAGCTCTCTACTGCATAGTCACAGACTAGCCACGAAG |
| ZBB239 | GTCGAGCTCTCTACTGCATACGCGAAGATGTCGGAAATTC |
| ZBB240 | GTCGAGCTCTCTACTGCATATAGCTGCTGGAATGCTTGTC |
| ZBB241 | GTCGAGCTCTCTACTGCATAGCACCATAGAGAGCTGTGTC |
| ZBB242 | GTCGAGCTCTCTACTGCATAGCAGGCATTATGTCCAAACG |
| ZBB243 | GTCGAGCTCTCTACTGCATACAAAGAGTTGCGAATACGGC |
| ZBB244 | GTCGAGCTCTCTACTGCATACCAAATAAAGCATGGTCGCC |
| ZBB245 | GTCGAGCTCTCTACTGCATATGAGGAACACGGTGCAATAC |
| ZBB246 | GTCGAGCTCTCTACTGCATATCAAGCGAATCCCATGTGAC |
| ZBB247 | GTCGAGCTCTCTACTGCATATTGCCATACACAGGAACGAC |
| ZBB248 | GTCGAGCTCTCTACTGCATACCAGAAGCACGTTTGACAAC |
| ZBB249 | GTCGAGCTCTCTACTGCATAGTATTCAACACCGGCGAAAC |
| ZBB250 | GTCGAGCTCTCTACTGCATAAAACGATGGGCAAGTGCTTA |
| ZBB251 | GTCGAGCTCTCTACTGCATATACGAACACCGTCGCAATTA |
| ZBB252 | GTCGAGCTCTCTACTGCATAGCCGATAACACATAGCCGTA |
| ZBB253 | GTCGAGCTCTCTACTGCATAGATACGCGAACATACCGTGA |
| ZBB254 | GTCGAGCTCTCTACTGCATATGCAGAACAGCAAGTTTGGA |
| ZBB255 | GTCGAGCTCTCTACTGCATAACGAGCGCAAGATATTTGGA |
| ZBB256 | GTCGAGCTCTCTACTGCATAGCGCATACGATACAATGGGA |
| ZBB257 | GTCGAGCTCTCTACTGCATAAAGCCGGTCGTAACTTGTAG |
| ZBB258 | GTCGAGCTCTCTACTGCATAACGGTTACCTAAGCGTTCAG |
| ZBB259 | GTCGAGCTCTCTACTGCATACGGCAGGTTGTACCAATTTG |
| ZBB260 | GTCGAGCTCTCTACTGCATACCATAGGTTTAACGCTTGCG |
| ZBB261 | GTCGAGCTCTCTACTGCATACATTAACGTGTTGGAAGCCG |
| ZBB262 | GTCGAGCTCTCTACTGCATACCATCTTCGTAAGGGCGTAG |
| ZBB263 | GTCGAGCTCTCTACTGCATAACGGACTTGCGATTAGCTTT |
| ZBB264 | GTCGAGCTCTCTACTGCATAAAATGATCGTTCAGCGGGTT |
| ZBB265 | GTCGAGCTCTCTACTGCATAACGTCGTACTCTAGCTCGTT |
| ZBB266 | GTCGAGCTCTCTACTGCATAAAACCTGAGTTCGCACAGTT |
| ZBB267 | GTCGAGCTCTCTACTGCATAAATAGCTGGTTAAGCCGTCG |
| ZBB268 | GTCGAGCTCTCTACTGCATAAAGTCCGCTTAACCTATGCG |
| ZBB269 | GTCGAGCTCTCTACTGCATAAATACTCCTTAACTGCGGCG |
| ZBB270 | GTCGAGCTCTCTACTGCATAAATGTACTGTAAGTCCGGCG |
| ZBB271 | GTCGAGCTCTCTACTGCATAAATCCTTGGTAAGAGTCGCG |
| ZBB272 | GTCGAGCTCTCTACTGCATAAAGTACGTGTAATCCTCGCG |
| ZBB273 | GTCGAGCTCTCTACTGCATAAATATCGACGAAGGGCGTAC |
| ZBB274 | GTCGAGCTCTCTACTGCATAAGACGTTCGCAATGGGTTTA |
| ZBB275 | GTCGAGCTCTCTACTGCATAAACATTCGTAAAGGTGGCGA |
| ZBB276 | GTCGAGCTCTCTACTGCATAGGCTAACGTACAGGACACTC |
| ZBB277 | GTCGAGCTCTCTACTGCATAGAAGCAATAACAGCCGTTCC |
| ZBB278 | GTCGAGCTCTCTACTGCATAGGGCATCTTAAACCACACGA |
| ZBB279 | GTCGAGCTCTCTACTGCATAGCCGTACACACAAGGTTACA |
| ZBB280 | GTCGAGCTCTCTACTGCATAGCCGTAGCCACAAGAGTTAA |
| ZBB281 | GTCGAGCTCTCTACTGCATAGGTGACATCAAACGCCCTAA |
| ZBB282 | GTCGAGCTCTCTACTGCATAGGTCTATGCAAACACCCGAA |
| ZBB283 | GTCGAGCTCTCTACTGCATACGGATTCATCGCGTACTTCT |
| ZBB284 | GTCGAGCTCTCTACTGCATACTCTAGCGGAGACGACTTTG |
| ZBB285 | GTCGAGCTCTCTACTGCATATACCGATTTACAGGTGCGTG |
| ZBB286 | GTCGAGCTCTCTACTGCATATGCGCCCTAAACAGCTTAAA |
| ZBB287 | GTCGAGCTCTCTACTGCATAGTACTCCGGTTCAGCATGTT |
| ZBB288 | GTCGAGCTCTCTACTGCATAGACCGTTAGCGGATAGCATT |
| ZBB289 | GTCGAGCTCTCTACTGCATAGCCGCTACCTTGACTGAATT |
| ZBB290 | GTCGAGCTCTCTACTGCATAGACGTTTAGGCACTACTGCT |
| ZBB291 | GTCGAGCTCTCTACTGCATACAGAATGGTAAATCCCGCGA |
| ZBB292 | GTCGAGCTCTCTACTGCATACTTTCGGAGCAAGAAGACGA |
| ZBB293 | GTCGAGCTCTCTACTGCATAAGCGCGTAGTTCAGTTGATT |
| ZBB294 | GTCGAGCTCTCTACTGCATAAGCTAGTTGTACTCAGCCGT |
| ZBB295 | GTCGAGCTCTCTACTGCATAAATTCGATGGCAGCGGTTAT |
| ZBB296 | GTCGAGCTCTCTACTGCATAATACGGAGTCGAGTCGCTAT |
| ZBB297 | GTCGAGCTCTCTACTGCATAAGACGGACGCGATCCTATAT |
| ZBB298 | GTCGAGCTCTCTACTGCATAAACTTTGCGGCAGTAGTGAT |
| ZBB299 | GTCGAGCTCTCTACTGCATAATCGAGGGACGAGTCTTCAT |
| ZBB300 | GTCGAGCTCTCTACTGCATAACGATGCACCTTCGCTAAAT |
| ZBB301 | GTCGAGCTCTCTACTGCATAAGTTATACCGCATCACGTCG |
| ZBB302 | GTCGAGCTCTCTACTGCATAAAGCTCTTCTAACGTCTGCG |
| ZBB303 | GTCGAGCTCTCTACTGCATAAATGTATCCTAATGGCCGCG |
| ZBB304 | GTCGAGCTCTCTACTGCATAATAGCTCATTAACCGGCACG |
| ZBB305 | GTCGAGCTCTCTACTGCATAACAGTTTAGGAAGCTCGTGC |
| ZBB306 | GTCGAGCTCTCTACTGCATAAACGTATCGGAAGCATAGGC |
| ZBB307 | GTCGAGCTCTCTACTGCATAACATTTAGGGAACGCAGAGC |
| ZBB308 | GTCGAGCTCTCTACTGCATATACCTGATCCGATACGCTGT |
| ZBB309 | GTCGAGCTCTCTACTGCATATAATGACCTCGAATCGTCGC |
| ZBB310 | GTCGAGCTCTCTACTGCATATCGAACTCAAGACACCTGGA |
| ZBB311 | GTCGAGCTCTCTACTGCATACAGAGTCAGCAAGTCGCATA |
| ZBB312 | GTCGAGCTCTCTACTGCATATTATCACTGGCGAGAGAGCT |
| ZBB313 | GTCGAGCTCTCTACTGCATACGTGCAGCAGAATGATGAAC |
| ZBB314 | GTCGAGCTCTCTACTGCATACTTCATGTGTCAGGGCGATT |
| ZBB315 | GTCGAGCTCTCTACTGCATATCAGTTGACGCAGTGATCTG |
| ZBB316 | GTCGAGCTCTCTACTGCATAAGCATAGATGACCATGTCGC |
| ZBB317 | GTCGAGCTCTCTACTGCATAAGATGCTCATAACGCTGCTC |
| ZBB318 | GTCGAGCTCTCTACTGCATAAAGTCTTTAGCATGGCGCAT |
| ZBB319 | GTCGAGCTCTCTACTGCATATGATCTCTACGCACTTGCTG |
| ZBB320 | GTCGAGCTCTCTACTGCATAAGAGGCCATTCATTCACACG |
| ZBB321 | GTCGAGCTCTCTACTGCATACCATCGCGTGAATTATGCTC |
| ZBB322 | GTCGAGCTCTCTACTGCATAGCTCGCCATGAATAGCAAAC |
| ZBB323 | GTCGAGCTCTCTACTGCATACGGATCGTAGCATCTTCCTG |
| ZBB324 | GTCGAGCTCTCTACTGCATATCGCAAGCAAGAGATGTTCA |
| ZBB325 | GTCGAGCTCTCTACTGCATATTCTAAGCTGCATGTAGCGG |
| ZBB326 | GTCGAGCTCTCTACTGCATAGATCAGCGTACACTCTCAGG |
| ZBB327 | GTCGAGCTCTCTACTGCATAACGCGCTATTGATATGTCGT |
| ZBB328 | GTCGAGCTCTCTACTGCATACGACGACTGCTCACGATTAT |
| ZBB329 | GTCGAGCTCTCTACTGCATAGAGTGCTATCCACGAGACTG |
| ZBB330 | GTCGAGCTCTCTACTGCATAGCCGTAGTAACAAGCTCACA |
| ZBB331 | GTCGAGCTCTCTACTGCATACGCATTGTGACAGATGTTCG |
| ZBB332 | GTCGAGCTCTCTACTGCATACACGATTCAGCATCAGTTCG |
| ZBB333 | GTCGAGCTCTCTACTGCATACCTCACGAGCGATCTGTATG |
| ZBB334 | GTCGAGCTCTCTACTGCATAATATACGCACGCATTGGGAG |
| ZBB335 | GTCGAGCTCTCTACTGCATAGAGTCGGCTACACTGCTATG |
| ZBB336 | GTCGAGCTCTCTACTGCATAATAGCTGTCACACTTGCTCG |
| ZBB337 | GTCGAGCTCTCTACTGCATACGCCTGAGCTACAAAGATGA |
| ZBB338 | GTCGAGCTCTCTACTGCATACGAGTCAAGACACTGCATGA |
| ZBB339 | GTCGAGCTCTCTACTGCATAGCCGCGTAGACAATAGATCA |
| ZBB340 | GTCGAGCTCTCTACTGCATAGGCATCACTCAACAGCAGTA |
| ZBB341 | GTCGAGCTCTCTACTGCATACTCATACGTCGAGCTGGATG |
| ZBB342 | GTCGAGCTCTCTACTGCATAGCACCGAGTAGATAGCATCG |
| ZBB343 | GTCGAGCTCTCTACTGCATACGCAATCTGATGCAACTGAC |
| ZBB344 | GTCGAGCTCTCTACTGCATAAGCACTAGGCTTGAGCATTG |
| ZBB345 | GTCGAGCTCTCTACTGCATAAGAGCACTTGAATCCACTGC |
| ZBB346 | GTCGAGCTCTCTACTGCATATTGACACATCTCCGAAGAGC |
| ZBB347 | GTCGAGCTCTCTACTGCATACTCATAGACGAAGCGAGGAC |
| ZBB348 | GTCGAGCTCTCTACTGCATATGCAGAGACACAGAGTGCTA |
| ZBB349 | GTCGAGCTCTCTACTGCATATGCCACATACGATCTGATGC |
| ZBB350 | GTCGAGCTCTCTACTGCATACAGACTAGAGAATCGCCGAC |
| ZBB351 | GTCGAGCTCTCTACTGCATATAATTCCGCACAATCTGGCA |
| ZBB352 | GTCGAGCTCTCTACTGCATATAGTATGCGTCACTCGCTCT |
| ZBB353 | GTCGAGCTCTCTACTGCATATACACTGCTGCATGTCTGTG |
| ZBB354 | GTCGAGCTCTCTACTGCATAAGAGCGCAGTCAGATTCATG |
| ZBB355 | GTCGAGCTCTCTACTGCATATGACGTGAGAGCTACTCGAT |
| ZBB356 | GTCGAGCTCTCTACTGCATACTGGAGACACGATAGACAGC |
| ZBB357 | GTCGAGCTCTCTACTGCATACTATGTAGGCGCATGACGAT |
| ZBB358 | GTCGAGCTCTCTACTGCATACAGACGCATGAACTTGAAGC |
| ZBB359 | GTCGAGCTCTCTACTGCATAGCATATAGCCAACGCGACTA |
| ZBB360 | GTCGAGCTCTCTACTGCATAGCGACAATCCAATTTACGCA |
| ZBB361 | GTCGAGCTCTCTACTGCATAACTATCGTCGAGATGGTGCT |
| ZBB362 | GTCGAGCTCTCTACTGCATACGACACGCTACAGAGGTATC |
| ZBB363 | GTCGAGCTCTCTACTGCATAACGATGGAGACATGCGTATC |
| ZBB364 | GTCGAGCTCTCTACTGCATAGCCAGATGTCAACACAGCTA |
| ZBB365 | GTCGAGCTCTCTACTGCATAAACTTCATAAACTGCACGCG |
| ZBB366 | GTCGAGCTCTCTACTGCATAAAGTTTATACAAGACGCGCC |
| ZBB367 | GTCGAGCTCTCTACTGCATAATGGCGTTACAATAAGGTCG |
| ZBB368 | GTCGAGCTCTCTACTGCATACACGGAATAATACATCCTCG |
| ZBB369 | GTCGAGCTCTCTACTGCATACCTAGCGGAAGGACAGTCAA |
| ZBB370 | GTCGAGCTCTCTACTGCATACCTGGCATACACGCTAATAG |
| ZBB371 | GTCGAGCTCTCTACTGCATAGATGCTCTAAGTTATTGGCC |
| ZBB372 | GTCGAGCTCTCTACTGCATAGATGTATTACGCACGGAGCA |
| ZBB373 | GTCGAGCTCTCTACTGCATAGCCGATTTACCCATCTGTAT |
| ZBB374 | GTCGAGCTCTCTACTGCATATTGATGTTAGCTGCCTAGCG |
| ZBB375 | GTCGAGCTCTCTACTGCATAAACCCAGAACAACTGCGGTG |
| ZBB376 | GTCGAGCTCTCTACTGCATAACCGACCAACAGATACGGGT |
| ZBB377 | GTCGAGCTCTCTACTGCATAAGCCAAGAACGAGAGCGTCT |
| ZBB378 | GTCGAGCTCTCTACTGCATACCAGACGGAAGCGAATCACT |
| ZBB379 | GTCGAGCTCTCTACTGCATACGATGTCGAGACCCACGAAT |
| ZBB380 | GTCGAGCTCTCTACTGCATAGGGTGCTCACACTTCGCATT |
| ZBB381 | GTCGAGCTCTCTACTGCATAGGTTCCGACGACTCTGCATT |
| ZBB382 | GTCGAGCTCTCTACTGCATAGGTTCGGCACCTGATCTCAT |
| ZBB383 | GTCGAGCTCTCTACTGCATATCCTCCGGCCAGTGATGTTA |
| ZBB384 | GTCGAGCTCTCTACTGCATATTCGGCGACTTGAGGTCACT |
| ZCC001 | GAACCCGGGACAAGGTGTCATCATAGATGTCAGCACTGGG |
| ZCC002 | GAACCCGGGACAAGGTGTCATAACTCAGTCTGGTAAGCGG |
| ZCC003 | GAACCCGGGACAAGGTGTCAAGTATATTGCTACCCACCGG |
| ZCC004 | GAACCCGGGACAAGGTGTCAACTATCATGTGGGTAACCGG |
| ZCC005 | GAACCCGGGACAAGGTGTCACATGATGCAGAAGAGTCCAC |
| ZCC006 | GAACCCGGGACAAGGTGTCAACTGCCTAGCAAGCATGATA |
| ZCC007 | GAACCCGGGACAAGGTGTCAGTCCATCAGACCAAGTACGA |
| ZCC008 | GAACCCGGGACAAGGTGTCACTGATGCGACTCCTAGACTT |
| ZCC009 | GAACCCGGGACAAGGTGTCAATCAGTAGTCCAGTGCAGTG |
| ZCC010 | GAACCCGGGACAAGGTGTCAGTTCCCATTCCATGTGATCG |
| ZCC011 | GAACCCGGGACAAGGTGTCATACCTACAGTTCCTGAAGCG |
| ZCC012 | GAACCCGGGACAAGGTGTCAGGGAAGAAATTAACCGTCCG |
| ZCC013 | GAACCCGGGACAAGGTGTCAAGATTTCTACCAGACTGCCG |
| ZCC014 | GAACCCGGGACAAGGTGTCAATAAGCTCTGGGAATAGCCG |
| ZCC015 | GAACCCGGGACAAGGTGTCACTACAGGGTACAGTATCCCG |
| ZCC016 | GAACCCGGGACAAGGTGTCAGATAGGACTCTCCGAAGCTC |
| ZCC017 | GAACCCGGGACAAGGTGTCAATGAGGACTTGACTGCATGT |
| ZCC018 | GAACCCGGGACAAGGTGTCAATGCTGCCAGGTGAGATAAT |
| ZCC019 | GAACCCGGGACAAGGTGTCAACTCAGTCACGATGATCCTG |
| ZCC020 | GAACCCGGGACAAGGTGTCAAGAGACGAGGCATTCCATAG |
| ZCC021 | GAACCCGGGACAAGGTGTCACTCCGTTGAATCCCATTGAG |
| ZCC022 | GAACCCGGGACAAGGTGTCACACCTATCAGGAGACGTGAG |
| ZCC023 | GAACCCGGGACAAGGTGTCAAATAGCAGATACGGCCTGAG |
| ZCC024 | GAACCCGGGACAAGGTGTCAATGTACCAGCCAGATACCAG |
| ZCC025 | GAACCCGGGACAAGGTGTCAGGATGGCTCACACTTCTTTC |
| ZCC026 | GAACCCGGGACAAGGTGTCATAAGGACGGCTAACCTCTTC |
| ZCC027 | GAACCCGGGACAAGGTGTCAAGGATTAGCCCATCTACACG |
| ZCC028 | GAACCCGGGACAAGGTGTCAGGCCAGCCATTCATATAACG |
| ZCC029 | GAACCCGGGACAAGGTGTCAAGATAGGACTTCCCACAACG |
| ZCC030 | GAACCCGGGACAAGGTGTCAGAAGACCCATGCACTGAATC |
| ZCC031 | GAACCCGGGACAAGGTGTCAACCCACAATAAAGGTGTTGC |
| ZCC032 | GAACCCGGGACAAGGTGTCACCCATTGTTTCAAGCATTGC |
| ZCC033 | GAACCCGGGACAAGGTGTCAACCATAGCTGAATACCAGGC |
| ZCC034 | GAACCCGGGACAAGGTGTCAGCATACATCAGCACTAAGGC |
| ZCC035 | GAACCCGGGACAAGGTGTCAACATAGGTAACAGGAGTCGC |
| ZCC036 | GAACCCGGGACAAGGTGTCATAGATACCACCAGAGGGTCC |
| ZCC037 | GAACCCGGGACAAGGTGTCATGAGGTCTAACATCAGCTCC |
| ZCC038 | GAACCCGGGACAAGGTGTCAAAGGCGACGGAAGTATATCC |
| ZCC039 | GAACCCGGGACAAGGTGTCATAGCAGAGACCCTAATTGCC |
| ZCC040 | GAACCCGGGACAAGGTGTCAAGCACACCATTGAATTGACC |
| ZCC041 | GAACCCGGGACAAGGTGTCATAGAGTTCCACACAGTGACC |
| ZCC042 | GAACCCGGGACAAGGTGTCAAAGGGATCTCGATAACCACC |
| ZCC043 | GAACCCGGGACAAGGTGTCAGTGCCCACATCGAATAAGAC |
| ZCC044 | GAACCCGGGACAAGGTGTCACCTCAGGAAGGAGAAGTCAC |
| ZCC045 | GAACCCGGGACAAGGTGTCATGTCCACACAGGTAGAACAC |
| ZCC046 | GAACCCGGGACAAGGTGTCAGGGAGACACATTTCACCAAC |
| ZCC047 | GAACCCGGGACAAGGTGTCAGCCCTCCGACAGAATAAGTA |
| ZCC048 | GAACCCGGGACAAGGTGTCAGGCCAGGAACTAACGATCTA |
| ZCC049 | GAACCCGGGACAAGGTGTCAAGACACCATGTGCCAATCTA |
| ZCC050 | GAACCCGGGACAAGGTGTCAGTGCCCACACTCAAGAGATA |
| ZCC051 | GAACCCGGGACAAGGTGTCACTTCGAGGGCAACAGATAGA |
| ZCC052 | GAACCCGGGACAAGGTGTCAGGGAACCACCCTAAATGTCA |
| ZCC053 | GAACCCGGGACAAGGTGTCAGTTGGTCATCCACAAACACA |
| ZCC054 | GAACCCGGGACAAGGTGTCACGGTCCAACACCAATTTGAA |
| ZCC055 | GAACCCGGGACAAGGTGTCACCCTGGGTACAAAGTGGAAA |
| ZCC056 | GAACCCGGGACAAGGTGTCAAATGGTATCGGACCCTGTTT |
| ZCC057 | GAACCCGGGACAAGGTGTCAAAGTCAGGGTTCAGTTCGTT |
| ZCC058 | GAACCCGGGACAAGGTGTCAAATTTCTAGTCACCGGGCTT |
| ZCC059 | GAACCCGGGACAAGGTGTCAAGACCGGGTTCATGGTTATT |
| ZCC060 | GAACCCGGGACAAGGTGTCATAGACCTTCCTAAGTTGCCG |
| ZCC061 | GAACCCGGGACAAGGTGTCATTCCGTTGAACAAGGGAGAA |
| ZCC062 | GAACCCGGGACAAGGTGTCAGAGGGCCTATTCCTTCCATT |
| ZCC063 | GAACCCGGGACAAGGTGTCAGCGGGCACCTTATTATACCT |
| ZCC064 | GAACCCGGGACAAGGTGTCACCGGAAGGATGTTTGCATTT |
| ZCC065 | GAACCCGGGACAAGGTGTCACACTTCTGACTAGGTGGGTT |
| ZCC066 | GAACCCGGGACAAGGTGTCACCCTTGATGGGACTGTAGTT |
| ZCC067 | GAACCCGGGACAAGGTGTCACTACCGTGGAGATTGGACTT |
| ZCC068 | GAACCCGGGACAAGGTGTCACCGTTGGAATAAGCAGGAAC |
| ZCC069 | GAACCCGGGACAAGGTGTCACCGAGTAGGAAACCTTAGCA |
| ZCC070 | GAACCCGGGACAAGGTGTCACCTGTTAGGAAACCCAGACA |
| ZCC071 | GAACCCGGGACAAGGTGTCAAATACTCTCTGACGGGAGGT |
| ZCC072 | GAACCCGGGACAAGGTGTCAAAAGGTCATGTCTCCGAAGT |
| ZCC073 | GAACCCGGGACAAGGTGTCAAGGGTCCTGTCACCTATTCT |
| ZCC074 | GAACCCGGGACAAGGTGTCAAATTAGATGGGACGTTGCCT |
| ZCC075 | GAACCCGGGACAAGGTGTCAAGATAGGTCGTCACTTCCCT |
| ZCC076 | GAACCCGGGACAAGGTGTCAATGGTGGCTACCCTATACCT |
| ZCC077 | GAACCCGGGACAAGGTGTCAACCTTGTGGGTAATGCGTAT |
| ZCC078 | GAACCCGGGACAAGGTGTCAAAGGAGATGTAACGCCCTAG |
| ZCC079 | GAACCCGGGACAAGGTGTCAACCGTACTGATAAGGGCTTC |
| ZCC080 | GAACCCGGGACAAGGTGTCAAAGAGTGACGTAACCTCCTC |
| ZCC081 | GAACCCGGGACAAGGTGTCAATAGTTGGACCACCGTACTC |
| ZCC082 | GAACCCGGGACAAGGTGTCAAAGAGGTAGTAACACTCCGC |
| ZCC083 | GAACCCGGGACAAGGTGTCAAAGGTCGGTCAAGTCTATCC |
| ZCC084 | GAACCCGGGACAAGGTGTCAAGATTCTAACCACGTAGGCC |
| ZCC085 | GAACCCGGGACAAGGTGTCAGTTCAGTGACGACTCCCTAT |
| ZCC086 | GAACCCGGGACAAGGTGTCAGAAGTCACCTTACAGGGCAT |
| ZCC087 | GAACCCGGGACAAGGTGTCAGAGGGATCTTCCACTCCTTG |
| ZCC088 | GAACCCGGGACAAGGTGTCAGAGACCCACTTAATGGCAAC |
| ZCC089 | GAACCCGGGACAAGGTGTCAGGAGAATCTACCGCAACCTA |
| ZCC090 | GAACCCGGGACAAGGTGTCAGGGAATCCGCAACACCTATA |
| ZCC091 | GAACCCGGGACAAGGTGTCAGGTCGGACACTACACCAATA |
| ZCC092 | GAACCCGGGACAAGGTGTCAGGACCTACCCAATGTCTTGA |
| ZCC093 | GAACCCGGGACAAGGTGTCACCAGCCTTGACAGTTTCTTG |
| ZCC094 | GAACCCGGGACAAGGTGTCACATCTACTACTAGGCGGGTG |
| ZCC095 | GAACCCGGGACAAGGTGTCACCCTGACGGAGATTAGTCTG |
| ZCC096 | GAACCCGGGACAAGGTGTCACCACTCTGTTAAGTTGCTGG |
| ZCC097 | GAACCCGGGACAAGGTGTCATATGGACTAGGAGCGTACCT |
| ZCC098 | GAACCCGGGACAAGGTGTCATGATTAGACCCATCCGGTTC |
| ZCC099 | GAACCCGGGACAAGGTGTCATAAGGACTGATAAGCCGGTC |
| ZCC100 | GAACCCGGGACAAGGTGTCATGACCATAACGAGTAAGGGC |
| ZCC101 | GAACCCGGGACAAGGTGTCATCCCAGGAAGTCAAGTGAAC |
| ZCC102 | GAACCCGGGACAAGGTGTCACGCCGGGATATAAAGGATCA |
| ZCC103 | GAACCCGGGACAAGGTGTCACCTCTGGCGAAATAGGAGAA |
| ZCC104 | GAACCCGGGACAAGGTGTCAACGACTCTGGGATGATGTTT |
| ZCC105 | GAACCCGGGACAAGGTGTCAAACCTATTGTGCATGGGAGT |
| ZCC106 | GAACCCGGGACAAGGTGTCAACTATCTATCGAGCGGGAGT |
| ZCC107 | GAACCCGGGACAAGGTGTCAATCCCTATGTGACGGAGAGT |
| ZCC108 | GAACCCGGGACAAGGTGTCAAAGGTCTAGTCAGTCTGCCT |
| ZCC109 | GAACCCGGGACAAGGTGTCAAGTTTAAGTGCAGTGAGCCT |
| ZCC110 | GAACCCGGGACAAGGTGTCAACTACCGTATCATGGGTGTG |
| ZCC111 | GAACCCGGGACAAGGTGTCAAAGTACGAGTCATGGACCTG |
| ZCC112 | GAACCCGGGACAAGGTGTCAATCATAGCGGTGGTAACCTG |
| ZCC113 | GAACCCGGGACAAGGTGTCAATCACTTATCGCAGGTAGGG |
| ZCC114 | GAACCCGGGACAAGGTGTCAATATGATACTCACGACCCGG |
| ZCC115 | GAACCCGGGACAAGGTGTCAATCTAAGCCTGCCGAACTAG |
| ZCC116 | GAACCCGGGACAAGGTGTCAACCCATTATACAGAGCGGTC |
| ZCC117 | GAACCCGGGACAAGGTGTCAACATAGCTTGAAGGGTCCTC |
| ZCC118 | GAACCCGGGACAAGGTGTCAAGCAGAATTAACGGCCTGTA |
| ZCC119 | GAACCCGGGACAAGGTGTCATGACCCGATCTCATTACAGG |
| ZCC120 | GAACCCGGGACAAGGTGTCATAATCATGGCGAAGGAGTCC |
| ZCC121 | GAACCCGGGACAAGGTGTCATCAGTAATAGGAAGGCAGCC |
| ZCC122 | GAACCCGGGACAAGGTGTCATGCATACCAGGGAATACACC |
| ZCC123 | GAACCCGGGACAAGGTGTCAGAGGATGCCTTCCCACATAT |
| ZCC124 | GAACCCGGGACAAGGTGTCAGACCAGGGTGTGCAATTATC |
| ZCC125 | GAACCCGGGACAAGGTGTCAGAGGGATACCCATCGACATC |
| ZCC126 | GAACCCGGGACAAGGTGTCAGGGCAACAAACATCTGCTTA |
| ZCC127 | GAACCCGGGACAAGGTGTCAGCTCGATAAGAACCCAGCTA |
| ZCC128 | GAACCCGGGACAAGGTGTCAGAGACCAGAACACTTGCCTA |
| ZCC129 | GAACCCGGGACAAGGTGTCACCCGAGTATCTGAGCTGATT |
| ZCC130 | GAACCCGGGACAAGGTGTCACCCAATGCTAAATGTGCAGA |
| ZCC131 | GAACCCGGGACAAGGTGTCAGAGCTGATCTCATGCTTCCT |
| ZCC132 | GAACCCGGGACAAGGTGTCAACAGTGGTACAATGGCTTGA |
| ZCC133 | GAACCCGGGACAAGGTGTCACTGAGAGTTCGATGACCTGT |
| ZCC134 | GAACCCGGGACAAGGTGTCAACTGTTCCAAGAGCTGCTTA |
| ZCC135 | GAACCCGGGACAAGGTGTCAAATCTCGCATTCCTGGACAT |
| ZCC136 | GAACCCGGGACAAGGTGTCATATAGGTCCACATGCAGCTC |
| ZCC137 | GAACCCGGGACAAGGTGTCAGTTTGCCATCAACACCAAGA |
| ZCC138 | GAACCCGGGACAAGGTGTCATAGTAAGAGCCAGGCACATC |
| ZCC139 | GAACCCGGGACAAGGTGTCATATAGTGCATCGTGCCCAAT |
| ZCC140 | GAACCCGGGACAAGGTGTCATATATGTCTACAGCGCCCTG |
| ZCC141 | GAACCCGGGACAAGGTGTCACAGATATGTGCAATCAGCCC |
| ZCC142 | GAACCCGGGACAAGGTGTCACCTCAGTAGAAAGCTGGACA |
| ZCC143 | GAACCCGGGACAAGGTGTCACGATGGTCTACATGGCTTTG |
| ZCC144 | GAACCCGGGACAAGGTGTCAGAATAGCTGCTCAACTGACC |
| ZCC145 | GAACCCGGGACAAGGTGTCATGCAGCAAATAATGTGGACC |
| ZCC146 | GAACCCGGGACAAGGTGTCACCATTGTCTGAAGCTGTGAG |
| ZCC147 | GAACCCGGGACAAGGTGTCAGCAAGTCATCTCAATGGACC |
| ZCC148 | GAACCCGGGACAAGGTGTCACTCGGATGGACAAGAAGTCA |
| ZCC149 | GAACCCGGGACAAGGTGTCACTCATCTGTGCAGGGAGTAT |
| ZCC150 | GAACCCGGGACAAGGTGTCAATATAGCGGCCTAAGCTGAG |
| ZCC151 | GAACCCGGGACAAGGTGTCACACAGCGAGGAATTATCACC |
| ZCC152 | GAACCCGGGACAAGGTGTCAAGATGCAGAACATGCTGGTA |
| ZCC153 | GAACCCGGGACAAGGTGTCACGACCTGGGATATGCAGTAT |
| ZCC154 | GAACCCGGGACAAGGTGTCAGGATGCCACTTCATACACTG |
| ZCC155 | GAACCCGGGACAAGGTGTCACCAGGATCGCAAGATACTGA |
| ZCC156 | GAACCCGGGACAAGGTGTCACTATTGGGCAGATGGCCTAT |
| ZCC157 | GAACCCGGGACAAGGTGTCAACATTCCATGAAGGAGGAGC |
| ZCC158 | GAACCCGGGACAAGGTGTCACTATCATTGAGGGTGCCAGT |
| ZCC159 | GAACCCGGGACAAGGTGTCATCCTAGTGAGGCACATGATG |
| ZCC160 | GAACCCGGGACAAGGTGTCACCATTGATGACAGTGCAGAG |
| ZCC161 | GAACCCGGGACAAGGTGTCAGGTCACACCAAATCATTGCA |
| ZCC162 | GAACCCGGGACAAGGTGTCAAAGCCTGCATTCACATTCAG |
| ZCC163 | GAACCCGGGACAAGGTGTCAGGCCATTACAAACTCATGCA |
| ZCC164 | GAACCCGGGACAAGGTGTCAGCCACTATGACAACTCAGGA |
| ZCC165 | GAACCCGGGACAAGGTGTCACGTGAGTACCAAGATCAGGA |
| ZCC166 | GAACCCGGGACAAGGTGTCATCACTATGCTCAGGGATGTG |
| ZCC167 | GAACCCGGGACAAGGTGTCAGCATGACATACAGGTACAGC |
| ZCC168 | GAACCCGGGACAAGGTGTCAGCCACTAGATTCAAGCAGAC |
| ZCC169 | GAACCCGGGACAAGGTGTCAGCTGAGATTAAAGCCCAGGA |
| ZCC170 | GAACCCGGGACAAGGTGTCAGCCATCTACCAATCGAAGGA |
| ZCC171 | GAACCCGGGACAAGGTGTCACTATGAAGGGAATGCACAGC |
| ZCC172 | GAACCCGGGACAAGGTGTCAACATCAGGAGAAGTGGTCAC |
| ZCC173 | GAACCCGGGACAAGGTGTCAGAAGATGACTGCAACCTCAC |
| ZCC174 | GAACCCGGGACAAGGTGTCAGAGACTAGATCCAGAGCCAG |
| ZCC175 | GAACCCGGGACAAGGTGTCAAGATGCTTAGGATGCCTGTT |
| ZCC176 | GAACCCGGGACAAGGTGTCACGTAGCATATCACCCTGGAT |
| ZCC177 | GAACCCGGGACAAGGTGTCAAGATATAGCTCACCCATGCG |
| ZCC178 | GAACCCGGGACAAGGTGTCACCTCGTCAGGTGATGAAGAT |
| ZCC179 | GAACCCGGGACAAGGTGTCATCATGGATTGCAGTCTCAGT |
| ZCC180 | GAACCCGGGACAAGGTGTCAATGAGTGAGCCCTCCCATAT |
| ZCC181 | GAACCCGGGACAAGGTGTCATCCTGAACCCTCATGTGATG |
| ZCC182 | GAACCCGGGACAAGGTGTCAGATAGCATCACATGACCTGC |
| ZCC183 | GAACCCGGGACAAGGTGTCACTGTCCAGAGAGGCAAAGTA |
| ZCC184 | GAACCCGGGACAAGGTGTCAGATAGATTCCCAGCTTTCGC |
| ZCC185 | GAACCCGGGACAAGGTGTCAATAGGACCCTCACGTTTACG |
| ZCC186 | GAACCCGGGACAAGGTGTCACCAATGGCAGGTAATGTACG |
| ZCC187 | GAACCCGGGACAAGGTGTCAACCTATTTGATGAGGCGGTT |
| ZCC188 | GAACCCGGGACAAGGTGTCACCAGTTGGCAAATCTTGTCA |
| ZCC189 | GAACCCGGGACAAGGTGTCAATATCTCCAGCATCTGAGCG |
| ZCC190 | GAACCCGGGACAAGGTGTCACTATCTACTGGCAGGAGCTG |
| ZCC191 | GAACCCGGGACAAGGTGTCAGCAGAAAGCTAAGTCACCTC |
| ZCC192 | GAACCCGGGACAAGGTGTCAAGAGTGTCCCAAGTTCCCTA |
| ZCC193 | GAACCCGGGACAAGGTGTCAGAGTGATAGCCCTCCACAAT |
| ZCC194 | GAACCCGGGACAAGGTGTCAGGCAGGCAGTTCATTCAAAT |
| ZCC195 | GAACCCGGGACAAGGTGTCAATATCATAGCCCACTGTCGG |
| ZCC196 | GAACCCGGGACAAGGTGTCAAGCACTAGCCCATTCTGTAG |
| ZCC197 | GAACCCGGGACAAGGTGTCACCAGAGGAGGAAGCATATCC |
| ZCC198 | GAACCCGGGACAAGGTGTCACAACATAGATGCCGGAAGTG |
| ZCC199 | GAACCCGGGACAAGGTGTCAGGAGTCCATCCATAGCAGTC |
| ZCC200 | GAACCCGGGACAAGGTGTCAACTAATCCAGCAGTAGCCAG |
| ZCC201 | GAACCCGGGACAAGGTGTCACCATGATCTAAAGAGCCCGA |
| ZCC202 | GAACCCGGGACAAGGTGTCAAGATAATGGAGATGCGCCTT |
| ZCC203 | GAACCCGGGACAAGGTGTCAGCACCTTCATCATGTCAGTG |
| ZCC204 | GAACCCGGGACAAGGTGTCAATGGTGCATGACCGGATATT |
| ZCC205 | GAACCCGGGACAAGGTGTCACATTCCGGGATAGCTGATGT |
| ZCC206 | GAACCCGGGACAAGGTGTCACCGCATCATGGAACATAGAC |
| ZCC207 | GAACCCGGGACAAGGTGTCAGCACCTCACCAATGATAGGA |
| ZCC208 | GAACCCGGGACAAGGTGTCAGTCTAGCCCAAATTGCACAA |
| ZCC209 | GAACCCGGGACAAGGTGTCAAGATCATACCGAAGACTGCC |
| ZCC210 | GAACCCGGGACAAGGTGTCAAGCTGTCCCTTCATCGAATT |
| ZCC211 | GAACCCGGGACAAGGTGTCACATCTACTCGCAGGATAGGG |
| ZCC212 | GAACCCGGGACAAGGTGTCACTCATCAGCTCATAGGAGGG |
| ZCC213 | GAACCCGGGACAAGGTGTCAAGAGACCACTCATTTCAGGG |
| ZCC214 | GAACCCGGGACAAGGTGTCACTATATGAGTACGGCCTCGG |
| ZCC215 | GAACCCGGGACAAGGTGTCATCAGGATGAGCCATTATCGG |
| ZCC216 | GAACCCGGGACAAGGTGTCAATCCCATAGTGTTAAGGCGG |
| ZCC217 | GAACCCGGGACAAGGTGTCAATATCTCACATGCCCGGAAG |
| ZCC218 | GAACCCGGGACAAGGTGTCACCTCAGGACAAAGTGATCCA |
| ZCC219 | GAACCCGGGACAAGGTGTCAGATCAGTGAACATCAGTGCC |
| ZCC220 | GAACCCGGGACAAGGTGTCAGGATATTGCCTCACACATCG |
| ZCC221 | GAACCCGGGACAAGGTGTCAAAGAGCATCCTACAGGTACG |
| ZCC222 | GAACCCGGGACAAGGTGTCAATGACAAGAGGGTCGAACTC |
| ZCC223 | GAACCCGGGACAAGGTGTCACATCAGGACCGTACAGAGAG |
| ZCC224 | GAACCCGGGACAAGGTGTCAACTGCCATACGAGGTAAGAG |
| ZCC225 | GAACCCGGGACAAGGTGTCAGAGAGCCGATCTAACCTCAG |
| ZCC226 | GAACCCGGGACAAGGTGTCAGGAGCCACGACCTATATCAG |
| ZCC227 | GAACCCGGGACAAGGTGTCAAGCTCATACAGGTTCCGAAG |
| ZCC228 | GAACCCGGGACAAGGTGTCATTGTACGACACCTCCAGAAG |
| ZCC229 | GAACCCGGGACAAGGTGTCAGTATTGTCCACATGGCCTTC |
| ZCC230 | GAACCCGGGACAAGGTGTCAGGAGGCAGTACACTACCTTC |
| ZCC231 | GAACCCGGGACAAGGTGTCATGACAAAGCAGATGGCATTC |
| ZCC232 | GAACCCGGGACAAGGTGTCAAAGATACACTGGCCCATGTC |
| ZCC233 | GAACCCGGGACAAGGTGTCAATCGACCCAGGCATGATTAG |
| ZCC234 | GAACCCGGGACAAGGTGTCAAGCCCACTCAGCATTAGTAG |
| ZCC235 | GAACCCGGGACAAGGTGTCAAGGAACAGACCATCTACTGC |
| ZCC236 | GAACCCGGGACAAGGTGTCAGGAGACTATCCACAGACTGC |
| ZCC237 | GAACCCGGGACAAGGTGTCAGTGACCACTACAGTTCATGC |
| ZCC238 | GAACCCGGGACAAGGTGTCAAACCTACCTCGAATAGTGGC |
| ZCC239 | GAACCCGGGACAAGGTGTCAATCCCGGTTCAAGGTATAGC |
| ZCC240 | GAACCCGGGACAAGGTGTCACTTGACAGAGGACATGAAGC |
| ZCC241 | GAACCCGGGACAAGGTGTCATGACCTAATACACCGGAAGC |
| ZCC242 | GAACCCGGGACAAGGTGTCACAGGAGACATTCCATCAAGC |
| ZCC243 | GAACCCGGGACAAGGTGTCATTTCCAAACAAAGATGGGCC |
| ZCC244 | GAACCCGGGACAAGGTGTCAATACATACGTGGAGAAGGCC |
| ZCC245 | GAACCCGGGACAAGGTGTCAAGAGGTATAACACTCACGCC |
| ZCC246 | GAACCCGGGACAAGGTGTCAAGGAGTAACTTCAACGACCC |
| ZCC247 | GAACCCGGGACAAGGTGTCATGAGTACATAGACAGCACCC |
| ZCC248 | GAACCCGGGACAAGGTGTCATGGCAGAGAAGAACCTTTGA |
| ZCC249 | GAACCCGGGACAAGGTGTCACCACGGAGAACATATCCTGA |
| ZCC250 | GAACCCGGGACAAGGTGTCAGGACATCATACCAAGCCTGA |
| ZCC251 | GAACCCGGGACAAGGTGTCATCCGATCTACCAAGACAGGA |
| ZCC252 | GAACCCGGGACAAGGTGTCATTCGGAAACCTGGCTTAACT |
| ZCC253 | GAACCCGGGACAAGGTGTCATAAGCCCACGGACTTTAAGG |
| ZCC254 | GAACCCGGGACAAGGTGTCAGTAAGCCTTTGTAACCCTCG |
| ZCC255 | GAACCCGGGACAAGGTGTCACGGGCCTTCATAGTTACCTT |
| ZCC256 | GAACCCGGGACAAGGTGTCACCCTATTTGTTAAGCGTGGG |
| ZCC257 | GAACCCGGGACAAGGTGTCAAGACTTTATACACGGACCCG |
| ZCC258 | GAACCCGGGACAAGGTGTCAACAGGTCCGTAACTTGGTAG |
| ZCC259 | GAACCCGGGACAAGGTGTCAAATACCGGCTAACCGTAGAG |
| ZCC260 | GAACCCGGGACAAGGTGTCAAATAGGACTTAACCCGCCAG |
| ZCC261 | GAACCCGGGACAAGGTGTCAAAGAGGGACGAACCTGTTAC |
| ZCC262 | GAACCCGGGACAAGGTGTCAACCCTGGTTAAATTGGTGGA |
| ZCC263 | GAACCCGGGACAAGGTGTCATAAGTCTCCCTAGCTCGGTT |
| ZCC264 | GAACCCGGGACAAGGTGTCATACTACCCTGGAGTGGGATT |
| ZCC265 | GAACCCGGGACAAGGTGTCATAGTGACTTAGACCCTCCGT |
| ZCC266 | GAACCCGGGACAAGGTGTCAGGACCCACTAAACGGTATCA |
| ZCC267 | GAACCCGGGACAAGGTGTCAGGGCACCCGAAATACTCTAA |
| ZCC268 | GAACCCGGGACAAGGTGTCACCAGACGCTTTAATTGGAGG |
| ZCC269 | GAACCCGGGACAAGGTGTCAAGACGGGATTCTGTCAGTTT |
| ZCC270 | GAACCCGGGACAAGGTGTCAAATCCCGTATGAGTGGTGTT |
| ZCC271 | GAACCCGGGACAAGGTGTCAAGGGAACTGTCGATTCTGTT |
| ZCC272 | GAACCCGGGACAAGGTGTCAAAAGCCACCTTCCAGATTGT |
| ZCC273 | GAACCCGGGACAAGGTGTCAACGCTAGACTGTTGGGAAAT |
| ZCC274 | GAACCCGGGACAAGGTGTCAATTAGTTAGCCAGCCTACCG |
| ZCC275 | GAACCCGGGACAAGGTGTCAAGACGTGTAGAAGCCCTTAC |
| ZCC276 | GAACCCGGGACAAGGTGTCAAATACCTTCAAAGTGGCCCA |
| ZCC277 | GAACCCGGGACAAGGTGTCATTACGGAGGCTTGGCATATT |
| ZCC278 | GAACCCGGGACAAGGTGTCATAGTACGGGTCAGTCTCTGT |
| ZCC279 | GAACCCGGGACAAGGTGTCAGGATAGGATACACTACCCGC |
| ZCC280 | GAACCCGGGACAAGGTGTCAGGGATAGAGCACCATAACCC |
| ZCC281 | GAACCCGGGACAAGGTGTCAGGTGCTACCAACCAACTAGA |
| ZCC282 | GAACCCGGGACAAGGTGTCAGGCCCTAATCTAAAGCCAGA |
| ZCC283 | GAACCCGGGACAAGGTGTCAGGCTAGAACCAATTCCCAGA |
| ZCC284 | GAACCCGGGACAAGGTGTCAGCCGGGCTTAAATTGAATCA |
| ZCC285 | GAACCCGGGACAAGGTGTCAGAGAATCGGAAACTCCTCCA |
| ZCC286 | GAACCCGGGACAAGGTGTCAGGTGACTCCAAAGAACTCCA |
| ZCC287 | GAACCCGGGACAAGGTGTCAGCCCGACTGAAATAGGTACA |
| ZCC288 | GAACCCGGGACAAGGTGTCACAGTATGCTAGATTCCGGGT |
| ZCC289 | GAACCCGGGACAAGGTGTCACGAGGTACATAAGGGCTCTC |
| ZCC290 | GAACCCGGGACAAGGTGTCACACAGGAGGTAACACTTTGC |
| ZCC291 | GAACCCGGGACAAGGTGTCATAATACCCGATCACTGGTGG |
| ZCC292 | GAACCCGGGACAAGGTGTCATTAATACGGATGCCCAGAGG |
| ZCC293 | GAACCCGGGACAAGGTGTCATGGCCCTCAAACCATGTAAA |
| ZCC294 | GAACCCGGGACAAGGTGTCAGGCTTTCACCGATAGTCTCT |
| ZCC295 | GAACCCGGGACAAGGTGTCAGATAGGCGTCTCACTTTCCT |
| ZCC296 | GAACCCGGGACAAGGTGTCACCTGTCGAAGAAGGAACTGA |
| ZCC297 | GAACCCGGGACAAGGTGTCACCACTGTGTAAAGACTCGGA |
| ZCC298 | GAACCCGGGACAAGGTGTCACATCAAGGGACACGTTAGGA |
| ZCC299 | GAACCCGGGACAAGGTGTCACCTGTCAGTAAACCGAGAGA |
| ZCC300 | GAACCCGGGACAAGGTGTCAAAGGATTGGTCAGATCGCTT |
| ZCC301 | GAACCCGGGACAAGGTGTCAATGTACTCCCTCTCAGGTGT |
| ZCC302 | GAACCCGGGACAAGGTGTCAAAGCTAGGGTCATTTGCTGT |
| ZCC303 | GAACCCGGGACAAGGTGTCAAAGGATCGTGTCATTCTGGT |
| ZCC304 | GAACCCGGGACAAGGTGTCAAGAGATCGGGCTATATCCGT |
| ZCC305 | GAACCCGGGACAAGGTGTCAACTGACTGTGCAGGTTTAGT |
| ZCC306 | GAACCCGGGACAAGGTGTCAAAGGAGCACTGGTTACACAT |
| ZCC307 | GAACCCGGGACAAGGTGTCAACACTCTAGTCAGGTGGTTG |
| ZCC308 | GAACCCGGGACAAGGTGTCAAAGCTCCTGTAACTCTAGCG |
| ZCC309 | GAACCCGGGACAAGGTGTCAACTCATCAGGTATGGGAACG |
| ZCC310 | GAACCCGGGACAAGGTGTCAAAGCTGGCCTAATGGTCTAG |
| ZCC311 | GAACCCGGGACAAGGTGTCAAAGGTACTGGAAGCTACTGC |
| ZCC312 | GAACCCGGGACAAGGTGTCAATAGTCCGGGAACTGTATGC |
| ZCC313 | GAACCCGGGACAAGGTGTCATATCAGGGCCTAGTGACTGT |
| ZCC314 | GAACCCGGGACAAGGTGTCATGACCATCAGTCTAACCGAG |
| ZCC315 | GAACCCGGGACAAGGTGTCATACATGGCACTAAGGGTCAG |
| ZCC316 | GAACCCGGGACAAGGTGTCATAAGAGTTCAGACCACCTGC |
| ZCC317 | GAACCCGGGACAAGGTGTCATTGTGCCAGACACCCAAATA |
| ZCC318 | GAACCCGGGACAAGGTGTCATCCCAGGAGATAAAGCCTCA |
| ZCC319 | GAACCCGGGACAAGGTGTCATGTCACCCAATAAAGTGCCA |
| ZCC320 | GAACCCGGGACAAGGTGTCATGACCATCCAAAGTGTCCAA |
| ZCC321 | GAACCCGGGACAAGGTGTCAGATAGTAGCCTCACGACCTG |
| ZCC322 | GAACCCGGGACAAGGTGTCAGACAGTCAGGGAATCCTAGC |
| ZCC323 | GAACCCGGGACAAGGTGTCAGCAACAAATGAATGGCTTCC |
| ZCC324 | GAACCCGGGACAAGGTGTCAGAGGTATGACCAGACATCCC |
| ZCC325 | GAACCCGGGACAAGGTGTCAGGCGATCACAGACATTAACC |
| ZCC326 | GAACCCGGGACAAGGTGTCACCTTGAGACCAATGCAGAGA |
| ZCC327 | GAACCCGGGACAAGGTGTCAGGCTCCCATCAATACAGAGA |
| ZCC328 | GAACCCGGGACAAGGTGTCATACAGCCTCACAGAGTGTTC |
| ZCC329 | GAACCCGGGACAAGGTGTCAGCCCTCGCATAGAAGACATA |
| ZCC330 | GAACCCGGGACAAGGTGTCAGATTGCTCACCAGACTTGTC |
| ZCC331 | GAACCCGGGACAAGGTGTCAGAGATCATGCCATTCAAGCC |
| ZCC332 | GAACCCGGGACAAGGTGTCAGAATCCATGCCAACCATTGA |
| ZCC333 | GAACCCGGGACAAGGTGTCAAGCAGAGCACCAGTCTAATC |
| ZCC334 | GAACCCGGGACAAGGTGTCAGACTGCCATAGACTGCATTC |
| ZCC335 | GAACCCGGGACAAGGTGTCACAGACAGAGACAGGTGTACC |
| ZCC336 | GAACCCGGGACAAGGTGTCAGATAGCTCTGTGCATCAGGT |
| ZCC337 | GAACCCGGGACAAGGTGTCACACTCAGAGGTAGCATCAGG |
| ZCC338 | GAACCCGGGACAAGGTGTCACAGCATCAGGAAGATTCACC |
| ZCC339 | GAACCCGGGACAAGGTGTCAACAGTGTACCAATGTGCTGA |
| ZCC340 | GAACCCGGGACAAGGTGTCAACTACTGCATAAGGGAGTGC |
| ZCC341 | GAACCCGGGACAAGGTGTCACCTGCATCAGCATTATGGAG |
| ZCC342 | GAACCCGGGACAAGGTGTCAACAGCTCTGCTAAGATGGAG |
| ZCC343 | GAACCCGGGACAAGGTGTCAAGGATTCTCACAATGCCTCA |
| ZCC344 | GAACCCGGGACAAGGTGTCAAGCTGCACTGGATTCATAGT |
| ZCC345 | GAACCCGGGACAAGGTGTCAAATCTGCATGAAGGTTGAGC |
| ZCC346 | GAACCCGGGACAAGGTGTCAACATCGTAGGAAGTCTGAGC |
| ZCC347 | GAACCCGGGACAAGGTGTCACCTTGCACAGAAGAGATGGA |
| ZCC348 | GAACCCGGGACAAGGTGTCACAACTACTGACAAGCTGGGA |
| ZCC349 | GAACCCGGGACAAGGTGTCAAAGCTCTCATCAGGAGTGTG |
| ZCC350 | GAACCCGGGACAAGGTGTCAATATCCGACTCAGCTCTGTG |
| ZCC351 | GAACCCGGGACAAGGTGTCAGATAAGTGCCAACAGCCCTA |
| ZCC352 | GAACCCGGGACAAGGTGTCACCTGACTACCAATGCAAGGA |
| ZCC353 | GAACCCGGGACAAGGTGTCACTCTAGTAGGGACCAGCATG |
| ZCC354 | GAACCCGGGACAAGGTGTCACTCCATCAGAGAGAGAGCAG |
| ZCC355 | GAACCCGGGACAAGGTGTCATAGAGTATCCCACGACATGC |
| ZCC356 | GAACCCGGGACAAGGTGTCAAGTATATCACCACGGACAGC |
| ZCC357 | GAACCCGGGACAAGGTGTCATGGCAGCTAATCAACACCTA |
| ZCC358 | GAACCCGGGACAAGGTGTCAGGACCCATGAAATCCTAGCA |
| ZCC359 | GAACCCGGGACAAGGTGTCACCATCTGGGTTAAGTGCATG |
| ZCC360 | GAACCCGGGACAAGGTGTCACCATTGATATAAGGGCGCAG |
| ZCC361 | GAACCCGGGACAAGGTGTCACAGTTATAGCCATTGAGCCG |
| ZCC362 | GAACCCGGGACAAGGTGTCAAGAGCTACAGTCAGTCACAG |
| ZCC363 | GAACCCGGGACAAGGTGTCAATAGATACCGCAGCTCACAG |
| ZCC364 | GAACCCGGGACAAGGTGTCAAGATAGTCTGAACACGCTCC |
| ZCC365 | GAACCCGGGACAAGGTGTCAAACCGTCTAAACCATACGTG |
| ZCC366 | GAACCCGGGACAAGGTGTCAAAGCTACAACAATGTCTCCG |
| ZCC367 | GAACCCGGGACAAGGTGTCAACGTGTCCAAAGGGTTATAC |
| ZCC368 | GAACCCGGGACAAGGTGTCAAGCTGGTAACAGGGATTAAC |
| ZCC369 | GAACCCGGGACAAGGTGTCACGATAAGCCAGTCTAATAGC |
| ZCC370 | GAACCCGGGACAAGGTGTCAGACTTTGTAATAGTGCTGCC |
| ZCC371 | GAACCCGGGACAAGGTGTCAGATAGCAATTAGTAGGAGCC |
| ZCC372 | GAACCCGGGACAAGGTGTCAGCTCATATACCTAGCCGTAG |
| ZCC373 | GAACCCGGGACAAGGTGTCATAGCTTCACTCATAGCCATG |
| ZCC374 | GAACCCGGGACAAGGTGTCATATTTCCTAGTAGTCCCAGC |
| ZCC375 | GAACCCGGGACAAGGTGTCAAACAATCTAAACGTGCGTCC |
| ZCC376 | GAACCCGGGACAAGGTGTCAACGATTCTAAAGGCTTCACC |
| ZCC377 | GAACCCGGGACAAGGTGTCAAGCCTCACAAATCTTTACGG |
| ZCC378 | GAACCCGGGACAAGGTGTCACAAGGTCTAAGAATCATGCC |
| ZCC379 | GAACCCGGGACAAGGTGTCACATACCAGAATAGAGTCGCC |
| ZCC380 | GAACCCGGGACAAGGTGTCACGATCTACAGAGGAATACGG |
| ZCC381 | GAACCCGGGACAAGGTGTCACTTCCTTCAATCGACTTAGG |
| ZCC382 | GAACCCGGGACAAGGTGTCAGATGAGCTAATCTGCGTCAA |
| ZCC383 | GAACCCGGGACAAGGTGTCAGCGTGAGGACGTATATTCAA |
| ZCC384 | GAACCCGGGACAAGGTGTCAGGTAGCCTAATGTGTGCTCA |
| ZCC001 | GAACCCGGGACAAGGTGTCATCATAGATGTCAGCACTGGG |
| ZCC002 | GAACCCGGGACAAGGTGTCATAACTCAGTCTGGTAAGCGG |
| ZCC003 | GAACCCGGGACAAGGTGTCAAGTATATTGCTACCCACCGG |
| ZCC004 | GAACCCGGGACAAGGTGTCAACTATCATGTGGGTAACCGG |
| ZCC005 | GAACCCGGGACAAGGTGTCACATGATGCAGAAGAGTCCAC |
| ZCC006 | GAACCCGGGACAAGGTGTCAACTGCCTAGCAAGCATGATA |
| ZCC007 | GAACCCGGGACAAGGTGTCAGTCCATCAGACCAAGTACGA |
| ZCC008 | GAACCCGGGACAAGGTGTCACTGATGCGACTCCTAGACTT |
| ZCC009 | GAACCCGGGACAAGGTGTCAATCAGTAGTCCAGTGCAGTG |
| ZCC010 | GAACCCGGGACAAGGTGTCAGTTCCCATTCCATGTGATCG |
| ZCC011 | GAACCCGGGACAAGGTGTCATACCTACAGTTCCTGAAGCG |
| ZCC012 | GAACCCGGGACAAGGTGTCAGGGAAGAAATTAACCGTCCG |
| ZCC013 | GAACCCGGGACAAGGTGTCAAGATTTCTACCAGACTGCCG |
| ZCC014 | GAACCCGGGACAAGGTGTCAATAAGCTCTGGGAATAGCCG |
| ZCC015 | GAACCCGGGACAAGGTGTCACTACAGGGTACAGTATCCCG |
| ZCC016 | GAACCCGGGACAAGGTGTCAGATAGGACTCTCCGAAGCTC |
| ZCC017 | GAACCCGGGACAAGGTGTCAATGAGGACTTGACTGCATGT |
| ZCC018 | GAACCCGGGACAAGGTGTCAATGCTGCCAGGTGAGATAAT |
| ZCC019 | GAACCCGGGACAAGGTGTCAACTCAGTCACGATGATCCTG |
| ZCC020 | GAACCCGGGACAAGGTGTCAAGAGACGAGGCATTCCATAG |
| ZCC021 | GAACCCGGGACAAGGTGTCACTCCGTTGAATCCCATTGAG |
| ZCC022 | GAACCCGGGACAAGGTGTCACACCTATCAGGAGACGTGAG |
| ZCC023 | GAACCCGGGACAAGGTGTCAAATAGCAGATACGGCCTGAG |
| ZCC024 | GAACCCGGGACAAGGTGTCAATGTACCAGCCAGATACCAG |
| ZCC025 | GAACCCGGGACAAGGTGTCAGGATGGCTCACACTTCTTTC |
| ZCC026 | GAACCCGGGACAAGGTGTCATAAGGACGGCTAACCTCTTC |
| ZCC027 | GAACCCGGGACAAGGTGTCAAGGATTAGCCCATCTACACG |
| ZCC028 | GAACCCGGGACAAGGTGTCAGGCCAGCCATTCATATAACG |
| ZCC029 | GAACCCGGGACAAGGTGTCAAGATAGGACTTCCCACAACG |
| ZCC030 | GAACCCGGGACAAGGTGTCAGAAGACCCATGCACTGAATC |
| ZCC031 | GAACCCGGGACAAGGTGTCAACCCACAATAAAGGTGTTGC |
| ZCC032 | GAACCCGGGACAAGGTGTCACCCATTGTTTCAAGCATTGC |
| ZCC033 | GAACCCGGGACAAGGTGTCAACCATAGCTGAATACCAGGC |
| ZCC034 | GAACCCGGGACAAGGTGTCAGCATACATCAGCACTAAGGC |
| ZCC035 | GAACCCGGGACAAGGTGTCAACATAGGTAACAGGAGTCGC |
| ZCC036 | GAACCCGGGACAAGGTGTCATAGATACCACCAGAGGGTCC |
| ZCC037 | GAACCCGGGACAAGGTGTCATGAGGTCTAACATCAGCTCC |
| ZCC038 | GAACCCGGGACAAGGTGTCAAAGGCGACGGAAGTATATCC |
| ZCC039 | GAACCCGGGACAAGGTGTCATAGCAGAGACCCTAATTGCC |
| ZCC040 | GAACCCGGGACAAGGTGTCAAGCACACCATTGAATTGACC |
| ZCC041 | GAACCCGGGACAAGGTGTCATAGAGTTCCACACAGTGACC |
| ZCC042 | GAACCCGGGACAAGGTGTCAAAGGGATCTCGATAACCACC |
| ZCC043 | GAACCCGGGACAAGGTGTCAGTGCCCACATCGAATAAGAC |
| ZCC044 | GAACCCGGGACAAGGTGTCACCTCAGGAAGGAGAAGTCAC |
| ZCC045 | GAACCCGGGACAAGGTGTCATGTCCACACAGGTAGAACAC |
| ZCC046 | GAACCCGGGACAAGGTGTCAGGGAGACACATTTCACCAAC |
| ZCC047 | GAACCCGGGACAAGGTGTCAGCCCTCCGACAGAATAAGTA |
| ZCC048 | GAACCCGGGACAAGGTGTCAGGCCAGGAACTAACGATCTA |
| ZCC049 | GAACCCGGGACAAGGTGTCAAGACACCATGTGCCAATCTA |
| ZCC050 | GAACCCGGGACAAGGTGTCAGTGCCCACACTCAAGAGATA |
| ZCC051 | GAACCCGGGACAAGGTGTCACTTCGAGGGCAACAGATAGA |
| ZCC052 | GAACCCGGGACAAGGTGTCAGGGAACCACCCTAAATGTCA |
| ZCC053 | GAACCCGGGACAAGGTGTCAGTTGGTCATCCACAAACACA |
| ZCC054 | GAACCCGGGACAAGGTGTCACGGTCCAACACCAATTTGAA |
| ZCC055 | GAACCCGGGACAAGGTGTCACCCTGGGTACAAAGTGGAAA |
| ZCC056 | GAACCCGGGACAAGGTGTCAAATGGTATCGGACCCTGTTT |
| ZCC057 | GAACCCGGGACAAGGTGTCAAAGTCAGGGTTCAGTTCGTT |
| ZCC058 | GAACCCGGGACAAGGTGTCAAATTTCTAGTCACCGGGCTT |
| ZCC059 | GAACCCGGGACAAGGTGTCAAGACCGGGTTCATGGTTATT |
| ZCC060 | GAACCCGGGACAAGGTGTCATAGACCTTCCTAAGTTGCCG |
| ZCC061 | GAACCCGGGACAAGGTGTCATTCCGTTGAACAAGGGAGAA |
| ZCC062 | GAACCCGGGACAAGGTGTCAGAGGGCCTATTCCTTCCATT |
| ZCC063 | GAACCCGGGACAAGGTGTCAGCGGGCACCTTATTATACCT |
| ZCC064 | GAACCCGGGACAAGGTGTCACCGGAAGGATGTTTGCATTT |
| ZCC065 | GAACCCGGGACAAGGTGTCACACTTCTGACTAGGTGGGTT |
| ZCC066 | GAACCCGGGACAAGGTGTCACCCTTGATGGGACTGTAGTT |
| ZCC067 | GAACCCGGGACAAGGTGTCACTACCGTGGAGATTGGACTT |
| ZCC068 | GAACCCGGGACAAGGTGTCACCGTTGGAATAAGCAGGAAC |
| ZCC069 | GAACCCGGGACAAGGTGTCACCGAGTAGGAAACCTTAGCA |
| ZCC070 | GAACCCGGGACAAGGTGTCACCTGTTAGGAAACCCAGACA |
| ZCC071 | GAACCCGGGACAAGGTGTCAAATACTCTCTGACGGGAGGT |
| ZCC072 | GAACCCGGGACAAGGTGTCAAAAGGTCATGTCTCCGAAGT |
| ZCC073 | GAACCCGGGACAAGGTGTCAAGGGTCCTGTCACCTATTCT |
| ZCC074 | GAACCCGGGACAAGGTGTCAAATTAGATGGGACGTTGCCT |
| ZCC075 | GAACCCGGGACAAGGTGTCAAGATAGGTCGTCACTTCCCT |
| ZCC076 | GAACCCGGGACAAGGTGTCAATGGTGGCTACCCTATACCT |
| ZCC077 | GAACCCGGGACAAGGTGTCAACCTTGTGGGTAATGCGTAT |
| ZCC078 | GAACCCGGGACAAGGTGTCAAAGGAGATGTAACGCCCTAG |
| ZCC079 | GAACCCGGGACAAGGTGTCAACCGTACTGATAAGGGCTTC |
| ZCC080 | GAACCCGGGACAAGGTGTCAAAGAGTGACGTAACCTCCTC |
| ZCC081 | GAACCCGGGACAAGGTGTCAATAGTTGGACCACCGTACTC |
| ZCC082 | GAACCCGGGACAAGGTGTCAAAGAGGTAGTAACACTCCGC |
| ZCC083 | GAACCCGGGACAAGGTGTCAAAGGTCGGTCAAGTCTATCC |
| ZCC084 | GAACCCGGGACAAGGTGTCAAGATTCTAACCACGTAGGCC |
| ZCC085 | GAACCCGGGACAAGGTGTCAGTTCAGTGACGACTCCCTAT |
| ZCC086 | GAACCCGGGACAAGGTGTCAGAAGTCACCTTACAGGGCAT |
| ZCC087 | GAACCCGGGACAAGGTGTCAGAGGGATCTTCCACTCCTTG |
| ZCC088 | GAACCCGGGACAAGGTGTCAGAGACCCACTTAATGGCAAC |
| ZCC089 | GAACCCGGGACAAGGTGTCAGGAGAATCTACCGCAACCTA |
| ZCC090 | GAACCCGGGACAAGGTGTCAGGGAATCCGCAACACCTATA |
| ZCC091 | GAACCCGGGACAAGGTGTCAGGTCGGACACTACACCAATA |
| ZCC092 | GAACCCGGGACAAGGTGTCAGGACCTACCCAATGTCTTGA |
| ZCC093 | GAACCCGGGACAAGGTGTCACCAGCCTTGACAGTTTCTTG |
| ZCC094 | GAACCCGGGACAAGGTGTCACATCTACTACTAGGCGGGTG |
| ZCC095 | GAACCCGGGACAAGGTGTCACCCTGACGGAGATTAGTCTG |
| ZCC096 | GAACCCGGGACAAGGTGTCACCACTCTGTTAAGTTGCTGG |
| ZCC097 | GAACCCGGGACAAGGTGTCATATGGACTAGGAGCGTACCT |
| ZCC098 | GAACCCGGGACAAGGTGTCATGATTAGACCCATCCGGTTC |
| ZCC099 | GAACCCGGGACAAGGTGTCATAAGGACTGATAAGCCGGTC |
| ZCC100 | GAACCCGGGACAAGGTGTCATGACCATAACGAGTAAGGGC |
| ZCC101 | GAACCCGGGACAAGGTGTCATCCCAGGAAGTCAAGTGAAC |
| ZCC102 | GAACCCGGGACAAGGTGTCACGCCGGGATATAAAGGATCA |
| ZCC103 | GAACCCGGGACAAGGTGTCACCTCTGGCGAAATAGGAGAA |
| ZCC104 | GAACCCGGGACAAGGTGTCAACGACTCTGGGATGATGTTT |
| ZCC105 | GAACCCGGGACAAGGTGTCAAACCTATTGTGCATGGGAGT |
| ZCC106 | GAACCCGGGACAAGGTGTCAACTATCTATCGAGCGGGAGT |
| ZCC107 | GAACCCGGGACAAGGTGTCAATCCCTATGTGACGGAGAGT |
| ZCC108 | GAACCCGGGACAAGGTGTCAAAGGTCTAGTCAGTCTGCCT |
| ZCC109 | GAACCCGGGACAAGGTGTCAAGTTTAAGTGCAGTGAGCCT |
| ZCC110 | GAACCCGGGACAAGGTGTCAACTACCGTATCATGGGTGTG |
| ZCC111 | GAACCCGGGACAAGGTGTCAAAGTACGAGTCATGGACCTG |
| ZCC112 | GAACCCGGGACAAGGTGTCAATCATAGCGGTGGTAACCTG |
| ZCC113 | GAACCCGGGACAAGGTGTCAATCACTTATCGCAGGTAGGG |
| ZCC114 | GAACCCGGGACAAGGTGTCAATATGATACTCACGACCCGG |
| ZCC115 | GAACCCGGGACAAGGTGTCAATCTAAGCCTGCCGAACTAG |
| ZCC116 | GAACCCGGGACAAGGTGTCAACCCATTATACAGAGCGGTC |
| ZCC117 | GAACCCGGGACAAGGTGTCAACATAGCTTGAAGGGTCCTC |
| ZCC118 | GAACCCGGGACAAGGTGTCAAGCAGAATTAACGGCCTGTA |
| ZCC119 | GAACCCGGGACAAGGTGTCATGACCCGATCTCATTACAGG |
| ZCC120 | GAACCCGGGACAAGGTGTCATAATCATGGCGAAGGAGTCC |
| ZCC121 | GAACCCGGGACAAGGTGTCATCAGTAATAGGAAGGCAGCC |
| ZCC122 | GAACCCGGGACAAGGTGTCATGCATACCAGGGAATACACC |
| ZCC123 | GAACCCGGGACAAGGTGTCAGAGGATGCCTTCCCACATAT |
| ZCC124 | GAACCCGGGACAAGGTGTCAGACCAGGGTGTGCAATTATC |
| ZCC125 | GAACCCGGGACAAGGTGTCAGAGGGATACCCATCGACATC |
| ZCC126 | GAACCCGGGACAAGGTGTCAGGGCAACAAACATCTGCTTA |
| ZCC127 | GAACCCGGGACAAGGTGTCAGCTCGATAAGAACCCAGCTA |
| ZCC128 | GAACCCGGGACAAGGTGTCAGAGACCAGAACACTTGCCTA |
| ZCC129 | GAACCCGGGACAAGGTGTCACCCGAGTATCTGAGCTGATT |
| ZCC130 | GAACCCGGGACAAGGTGTCACCCAATGCTAAATGTGCAGA |
| ZCC131 | GAACCCGGGACAAGGTGTCAGAGCTGATCTCATGCTTCCT |
| ZCC132 | GAACCCGGGACAAGGTGTCAACAGTGGTACAATGGCTTGA |
| ZCC133 | GAACCCGGGACAAGGTGTCACTGAGAGTTCGATGACCTGT |
| ZCC134 | GAACCCGGGACAAGGTGTCAACTGTTCCAAGAGCTGCTTA |
| ZCC135 | GAACCCGGGACAAGGTGTCAAATCTCGCATTCCTGGACAT |
| ZCC136 | GAACCCGGGACAAGGTGTCATATAGGTCCACATGCAGCTC |
| ZCC137 | GAACCCGGGACAAGGTGTCAGTTTGCCATCAACACCAAGA |
| ZCC138 | GAACCCGGGACAAGGTGTCATAGTAAGAGCCAGGCACATC |
| ZCC139 | GAACCCGGGACAAGGTGTCATATAGTGCATCGTGCCCAAT |
| ZCC140 | GAACCCGGGACAAGGTGTCATATATGTCTACAGCGCCCTG |
| ZCC141 | GAACCCGGGACAAGGTGTCACAGATATGTGCAATCAGCCC |
| ZCC142 | GAACCCGGGACAAGGTGTCACCTCAGTAGAAAGCTGGACA |
| ZCC143 | GAACCCGGGACAAGGTGTCACGATGGTCTACATGGCTTTG |
| ZCC144 | GAACCCGGGACAAGGTGTCAGAATAGCTGCTCAACTGACC |
| ZCC145 | GAACCCGGGACAAGGTGTCATGCAGCAAATAATGTGGACC |
| ZCC146 | GAACCCGGGACAAGGTGTCACCATTGTCTGAAGCTGTGAG |
| ZCC147 | GAACCCGGGACAAGGTGTCAGCAAGTCATCTCAATGGACC |
| ZCC148 | GAACCCGGGACAAGGTGTCACTCGGATGGACAAGAAGTCA |
| ZCC149 | GAACCCGGGACAAGGTGTCACTCATCTGTGCAGGGAGTAT |
| ZCC150 | GAACCCGGGACAAGGTGTCAATATAGCGGCCTAAGCTGAG |
| ZCC151 | GAACCCGGGACAAGGTGTCACACAGCGAGGAATTATCACC |
| ZCC152 | GAACCCGGGACAAGGTGTCAAGATGCAGAACATGCTGGTA |
| ZCC153 | GAACCCGGGACAAGGTGTCACGACCTGGGATATGCAGTAT |
| ZCC154 | GAACCCGGGACAAGGTGTCAGGATGCCACTTCATACACTG |
| ZCC155 | GAACCCGGGACAAGGTGTCACCAGGATCGCAAGATACTGA |
| ZCC156 | GAACCCGGGACAAGGTGTCACTATTGGGCAGATGGCCTAT |
| ZCC157 | GAACCCGGGACAAGGTGTCAACATTCCATGAAGGAGGAGC |
| ZCC158 | GAACCCGGGACAAGGTGTCACTATCATTGAGGGTGCCAGT |
| ZCC159 | GAACCCGGGACAAGGTGTCATCCTAGTGAGGCACATGATG |
| ZCC160 | GAACCCGGGACAAGGTGTCACCATTGATGACAGTGCAGAG |
| ZCC161 | GAACCCGGGACAAGGTGTCAGGTCACACCAAATCATTGCA |
| ZCC162 | GAACCCGGGACAAGGTGTCAAAGCCTGCATTCACATTCAG |
| ZCC163 | GAACCCGGGACAAGGTGTCAGGCCATTACAAACTCATGCA |
| ZCC164 | GAACCCGGGACAAGGTGTCAGCCACTATGACAACTCAGGA |
| ZCC165 | GAACCCGGGACAAGGTGTCACGTGAGTACCAAGATCAGGA |
| ZCC166 | GAACCCGGGACAAGGTGTCATCACTATGCTCAGGGATGTG |
| ZCC167 | GAACCCGGGACAAGGTGTCAGCATGACATACAGGTACAGC |
| ZCC168 | GAACCCGGGACAAGGTGTCAGCCACTAGATTCAAGCAGAC |
| ZCC169 | GAACCCGGGACAAGGTGTCAGCTGAGATTAAAGCCCAGGA |
| ZCC170 | GAACCCGGGACAAGGTGTCAGCCATCTACCAATCGAAGGA |
| ZCC171 | GAACCCGGGACAAGGTGTCACTATGAAGGGAATGCACAGC |
| ZCC172 | GAACCCGGGACAAGGTGTCAACATCAGGAGAAGTGGTCAC |
| ZCC173 | GAACCCGGGACAAGGTGTCAGAAGATGACTGCAACCTCAC |
| ZCC174 | GAACCCGGGACAAGGTGTCAGAGACTAGATCCAGAGCCAG |
| ZCC175 | GAACCCGGGACAAGGTGTCAAGATGCTTAGGATGCCTGTT |
| ZCC176 | GAACCCGGGACAAGGTGTCACGTAGCATATCACCCTGGAT |
| ZCC177 | GAACCCGGGACAAGGTGTCAAGATATAGCTCACCCATGCG |
| ZCC178 | GAACCCGGGACAAGGTGTCACCTCGTCAGGTGATGAAGAT |
| ZCC179 | GAACCCGGGACAAGGTGTCATCATGGATTGCAGTCTCAGT |
| ZCC180 | GAACCCGGGACAAGGTGTCAATGAGTGAGCCCTCCCATAT |
| ZCC181 | GAACCCGGGACAAGGTGTCATCCTGAACCCTCATGTGATG |
| ZCC182 | GAACCCGGGACAAGGTGTCAGATAGCATCACATGACCTGC |
| ZCC183 | GAACCCGGGACAAGGTGTCACTGTCCAGAGAGGCAAAGTA |
| ZCC184 | GAACCCGGGACAAGGTGTCAGATAGATTCCCAGCTTTCGC |
| ZCC185 | GAACCCGGGACAAGGTGTCAATAGGACCCTCACGTTTACG |
| ZCC186 | GAACCCGGGACAAGGTGTCACCAATGGCAGGTAATGTACG |
| ZCC187 | GAACCCGGGACAAGGTGTCAACCTATTTGATGAGGCGGTT |
| ZCC188 | GAACCCGGGACAAGGTGTCACCAGTTGGCAAATCTTGTCA |
| ZCC189 | GAACCCGGGACAAGGTGTCAATATCTCCAGCATCTGAGCG |
| ZCC190 | GAACCCGGGACAAGGTGTCACTATCTACTGGCAGGAGCTG |
| ZCC191 | GAACCCGGGACAAGGTGTCAGCAGAAAGCTAAGTCACCTC |
| ZCC192 | GAACCCGGGACAAGGTGTCAAGAGTGTCCCAAGTTCCCTA |
| ZCC193 | GAACCCGGGACAAGGTGTCAGAGTGATAGCCCTCCACAAT |
| ZCC194 | GAACCCGGGACAAGGTGTCAGGCAGGCAGTTCATTCAAAT |
| ZCC195 | GAACCCGGGACAAGGTGTCAATATCATAGCCCACTGTCGG |
| ZCC196 | GAACCCGGGACAAGGTGTCAAGCACTAGCCCATTCTGTAG |
| ZCC197 | GAACCCGGGACAAGGTGTCACCAGAGGAGGAAGCATATCC |
| ZCC198 | GAACCCGGGACAAGGTGTCACAACATAGATGCCGGAAGTG |
| ZCC199 | GAACCCGGGACAAGGTGTCAGGAGTCCATCCATAGCAGTC |
| ZCC200 | GAACCCGGGACAAGGTGTCAACTAATCCAGCAGTAGCCAG |
| ZCC201 | GAACCCGGGACAAGGTGTCACCATGATCTAAAGAGCCCGA |
| ZCC202 | GAACCCGGGACAAGGTGTCAAGATAATGGAGATGCGCCTT |
| ZCC203 | GAACCCGGGACAAGGTGTCAGCACCTTCATCATGTCAGTG |
| ZCC204 | GAACCCGGGACAAGGTGTCAATGGTGCATGACCGGATATT |
| ZCC205 | GAACCCGGGACAAGGTGTCACATTCCGGGATAGCTGATGT |
| ZCC206 | GAACCCGGGACAAGGTGTCACCGCATCATGGAACATAGAC |
| ZCC207 | GAACCCGGGACAAGGTGTCAGCACCTCACCAATGATAGGA |
| ZCC208 | GAACCCGGGACAAGGTGTCAGTCTAGCCCAAATTGCACAA |
| ZCC209 | GAACCCGGGACAAGGTGTCAAGATCATACCGAAGACTGCC |
| ZCC210 | GAACCCGGGACAAGGTGTCAAGCTGTCCCTTCATCGAATT |
| ZCC211 | GAACCCGGGACAAGGTGTCACATCTACTCGCAGGATAGGG |
| ZCC212 | GAACCCGGGACAAGGTGTCACTCATCAGCTCATAGGAGGG |
| ZCC213 | GAACCCGGGACAAGGTGTCAAGAGACCACTCATTTCAGGG |
| ZCC214 | GAACCCGGGACAAGGTGTCACTATATGAGTACGGCCTCGG |
| ZCC215 | GAACCCGGGACAAGGTGTCATCAGGATGAGCCATTATCGG |
| ZCC216 | GAACCCGGGACAAGGTGTCAATCCCATAGTGTTAAGGCGG |
| ZCC217 | GAACCCGGGACAAGGTGTCAATATCTCACATGCCCGGAAG |
| ZCC218 | GAACCCGGGACAAGGTGTCACCTCAGGACAAAGTGATCCA |
| ZCC219 | GAACCCGGGACAAGGTGTCAGATCAGTGAACATCAGTGCC |
| ZCC220 | GAACCCGGGACAAGGTGTCAGGATATTGCCTCACACATCG |
| ZCC221 | GAACCCGGGACAAGGTGTCAAAGAGCATCCTACAGGTACG |
| ZCC222 | GAACCCGGGACAAGGTGTCAATGACAAGAGGGTCGAACTC |
| ZCC223 | GAACCCGGGACAAGGTGTCACATCAGGACCGTACAGAGAG |
| ZCC224 | GAACCCGGGACAAGGTGTCAACTGCCATACGAGGTAAGAG |
| ZCC225 | GAACCCGGGACAAGGTGTCAGAGAGCCGATCTAACCTCAG |
| ZCC226 | GAACCCGGGACAAGGTGTCAGGAGCCACGACCTATATCAG |
| ZCC227 | GAACCCGGGACAAGGTGTCAAGCTCATACAGGTTCCGAAG |
| ZCC228 | GAACCCGGGACAAGGTGTCATTGTACGACACCTCCAGAAG |
| ZCC229 | GAACCCGGGACAAGGTGTCAGTATTGTCCACATGGCCTTC |
| ZCC230 | GAACCCGGGACAAGGTGTCAGGAGGCAGTACACTACCTTC |
| ZCC231 | GAACCCGGGACAAGGTGTCATGACAAAGCAGATGGCATTC |
| ZCC232 | GAACCCGGGACAAGGTGTCAAAGATACACTGGCCCATGTC |
| ZCC233 | GAACCCGGGACAAGGTGTCAATCGACCCAGGCATGATTAG |
| ZCC234 | GAACCCGGGACAAGGTGTCAAGCCCACTCAGCATTAGTAG |
| ZCC235 | GAACCCGGGACAAGGTGTCAAGGAACAGACCATCTACTGC |
| ZCC236 | GAACCCGGGACAAGGTGTCAGGAGACTATCCACAGACTGC |
| ZCC237 | GAACCCGGGACAAGGTGTCAGTGACCACTACAGTTCATGC |
| ZCC238 | GAACCCGGGACAAGGTGTCAAACCTACCTCGAATAGTGGC |
| ZCC239 | GAACCCGGGACAAGGTGTCAATCCCGGTTCAAGGTATAGC |
| ZCC240 | GAACCCGGGACAAGGTGTCACTTGACAGAGGACATGAAGC |
| ZCC241 | GAACCCGGGACAAGGTGTCATGACCTAATACACCGGAAGC |
| ZCC242 | GAACCCGGGACAAGGTGTCACAGGAGACATTCCATCAAGC |
| ZCC243 | GAACCCGGGACAAGGTGTCATTTCCAAACAAAGATGGGCC |
| ZCC244 | GAACCCGGGACAAGGTGTCAATACATACGTGGAGAAGGCC |
| ZCC245 | GAACCCGGGACAAGGTGTCAAGAGGTATAACACTCACGCC |
| ZCC246 | GAACCCGGGACAAGGTGTCAAGGAGTAACTTCAACGACCC |
| ZCC247 | GAACCCGGGACAAGGTGTCATGAGTACATAGACAGCACCC |
| ZCC248 | GAACCCGGGACAAGGTGTCATGGCAGAGAAGAACCTTTGA |
| ZCC249 | GAACCCGGGACAAGGTGTCACCACGGAGAACATATCCTGA |
| ZCC250 | GAACCCGGGACAAGGTGTCAGGACATCATACCAAGCCTGA |
| ZCC251 | GAACCCGGGACAAGGTGTCATCCGATCTACCAAGACAGGA |
| ZCC252 | GAACCCGGGACAAGGTGTCATTCGGAAACCTGGCTTAACT |
| ZCC253 | GAACCCGGGACAAGGTGTCATAAGCCCACGGACTTTAAGG |
| ZCC254 | GAACCCGGGACAAGGTGTCAGTAAGCCTTTGTAACCCTCG |
| ZCC255 | GAACCCGGGACAAGGTGTCACGGGCCTTCATAGTTACCTT |
| ZCC256 | GAACCCGGGACAAGGTGTCACCCTATTTGTTAAGCGTGGG |
| ZCC257 | GAACCCGGGACAAGGTGTCAAGACTTTATACACGGACCCG |
| ZCC258 | GAACCCGGGACAAGGTGTCAACAGGTCCGTAACTTGGTAG |
| ZCC259 | GAACCCGGGACAAGGTGTCAAATACCGGCTAACCGTAGAG |
| ZCC260 | GAACCCGGGACAAGGTGTCAAATAGGACTTAACCCGCCAG |
| ZCC261 | GAACCCGGGACAAGGTGTCAAAGAGGGACGAACCTGTTAC |
| ZCC262 | GAACCCGGGACAAGGTGTCAACCCTGGTTAAATTGGTGGA |
| ZCC263 | GAACCCGGGACAAGGTGTCATAAGTCTCCCTAGCTCGGTT |
| ZCC264 | GAACCCGGGACAAGGTGTCATACTACCCTGGAGTGGGATT |
| ZCC265 | GAACCCGGGACAAGGTGTCATAGTGACTTAGACCCTCCGT |
| ZCC266 | GAACCCGGGACAAGGTGTCAGGACCCACTAAACGGTATCA |
| ZCC267 | GAACCCGGGACAAGGTGTCAGGGCACCCGAAATACTCTAA |
| ZCC268 | GAACCCGGGACAAGGTGTCACCAGACGCTTTAATTGGAGG |
| ZCC269 | GAACCCGGGACAAGGTGTCAAGACGGGATTCTGTCAGTTT |
| ZCC270 | GAACCCGGGACAAGGTGTCAAATCCCGTATGAGTGGTGTT |
| ZCC271 | GAACCCGGGACAAGGTGTCAAGGGAACTGTCGATTCTGTT |
| ZCC272 | GAACCCGGGACAAGGTGTCAAAAGCCACCTTCCAGATTGT |
| ZCC273 | GAACCCGGGACAAGGTGTCAACGCTAGACTGTTGGGAAAT |
| ZCC274 | GAACCCGGGACAAGGTGTCAATTAGTTAGCCAGCCTACCG |
| ZCC275 | GAACCCGGGACAAGGTGTCAAGACGTGTAGAAGCCCTTAC |
| ZCC276 | GAACCCGGGACAAGGTGTCAAATACCTTCAAAGTGGCCCA |
| ZCC277 | GAACCCGGGACAAGGTGTCATTACGGAGGCTTGGCATATT |
| ZCC278 | GAACCCGGGACAAGGTGTCATAGTACGGGTCAGTCTCTGT |
| ZCC279 | GAACCCGGGACAAGGTGTCAGGATAGGATACACTACCCGC |
| ZCC280 | GAACCCGGGACAAGGTGTCAGGGATAGAGCACCATAACCC |
| ZCC281 | GAACCCGGGACAAGGTGTCAGGTGCTACCAACCAACTAGA |
| ZCC282 | GAACCCGGGACAAGGTGTCAGGCCCTAATCTAAAGCCAGA |
| ZCC283 | GAACCCGGGACAAGGTGTCAGGCTAGAACCAATTCCCAGA |
| ZCC284 | GAACCCGGGACAAGGTGTCAGCCGGGCTTAAATTGAATCA |
| ZCC285 | GAACCCGGGACAAGGTGTCAGAGAATCGGAAACTCCTCCA |
| ZCC286 | GAACCCGGGACAAGGTGTCAGGTGACTCCAAAGAACTCCA |
| ZCC287 | GAACCCGGGACAAGGTGTCAGCCCGACTGAAATAGGTACA |
| ZCC288 | GAACCCGGGACAAGGTGTCACAGTATGCTAGATTCCGGGT |
| ZCC289 | GAACCCGGGACAAGGTGTCACGAGGTACATAAGGGCTCTC |
| ZCC290 | GAACCCGGGACAAGGTGTCACACAGGAGGTAACACTTTGC |
| ZCC291 | GAACCCGGGACAAGGTGTCATAATACCCGATCACTGGTGG |
| ZCC292 | GAACCCGGGACAAGGTGTCATTAATACGGATGCCCAGAGG |
| ZCC293 | GAACCCGGGACAAGGTGTCATGGCCCTCAAACCATGTAAA |
| ZCC294 | GAACCCGGGACAAGGTGTCAGGCTTTCACCGATAGTCTCT |
| ZCC295 | GAACCCGGGACAAGGTGTCAGATAGGCGTCTCACTTTCCT |
| ZCC296 | GAACCCGGGACAAGGTGTCACCTGTCGAAGAAGGAACTGA |
| ZCC297 | GAACCCGGGACAAGGTGTCACCACTGTGTAAAGACTCGGA |
| ZCC298 | GAACCCGGGACAAGGTGTCACATCAAGGGACACGTTAGGA |
| ZCC299 | GAACCCGGGACAAGGTGTCACCTGTCAGTAAACCGAGAGA |
| ZCC300 | GAACCCGGGACAAGGTGTCAAAGGATTGGTCAGATCGCTT |
| ZCC301 | GAACCCGGGACAAGGTGTCAATGTACTCCCTCTCAGGTGT |
| ZCC302 | GAACCCGGGACAAGGTGTCAAAGCTAGGGTCATTTGCTGT |
| ZCC303 | GAACCCGGGACAAGGTGTCAAAGGATCGTGTCATTCTGGT |
| ZCC304 | GAACCCGGGACAAGGTGTCAAGAGATCGGGCTATATCCGT |
| ZCC305 | GAACCCGGGACAAGGTGTCAACTGACTGTGCAGGTTTAGT |
| ZCC306 | GAACCCGGGACAAGGTGTCAAAGGAGCACTGGTTACACAT |
| ZCC307 | GAACCCGGGACAAGGTGTCAACACTCTAGTCAGGTGGTTG |
| ZCC308 | GAACCCGGGACAAGGTGTCAAAGCTCCTGTAACTCTAGCG |
| ZCC309 | GAACCCGGGACAAGGTGTCAACTCATCAGGTATGGGAACG |
| ZCC310 | GAACCCGGGACAAGGTGTCAAAGCTGGCCTAATGGTCTAG |
| ZCC311 | GAACCCGGGACAAGGTGTCAAAGGTACTGGAAGCTACTGC |
| ZCC312 | GAACCCGGGACAAGGTGTCAATAGTCCGGGAACTGTATGC |
| ZCC313 | GAACCCGGGACAAGGTGTCATATCAGGGCCTAGTGACTGT |
| ZCC314 | GAACCCGGGACAAGGTGTCATGACCATCAGTCTAACCGAG |
| ZCC315 | GAACCCGGGACAAGGTGTCATACATGGCACTAAGGGTCAG |
| ZCC316 | GAACCCGGGACAAGGTGTCATAAGAGTTCAGACCACCTGC |
| ZCC317 | GAACCCGGGACAAGGTGTCATTGTGCCAGACACCCAAATA |
| ZCC318 | GAACCCGGGACAAGGTGTCATCCCAGGAGATAAAGCCTCA |
| ZCC319 | GAACCCGGGACAAGGTGTCATGTCACCCAATAAAGTGCCA |
| ZCC320 | GAACCCGGGACAAGGTGTCATGACCATCCAAAGTGTCCAA |
| ZCC321 | GAACCCGGGACAAGGTGTCAGATAGTAGCCTCACGACCTG |
| ZCC322 | GAACCCGGGACAAGGTGTCAGACAGTCAGGGAATCCTAGC |
| ZCC323 | GAACCCGGGACAAGGTGTCAGCAACAAATGAATGGCTTCC |
| ZCC324 | GAACCCGGGACAAGGTGTCAGAGGTATGACCAGACATCCC |
| ZCC325 | GAACCCGGGACAAGGTGTCAGGCGATCACAGACATTAACC |
| ZCC326 | GAACCCGGGACAAGGTGTCACCTTGAGACCAATGCAGAGA |
| ZCC327 | GAACCCGGGACAAGGTGTCAGGCTCCCATCAATACAGAGA |
| ZCC328 | GAACCCGGGACAAGGTGTCATACAGCCTCACAGAGTGTTC |
| ZCC329 | GAACCCGGGACAAGGTGTCAGCCCTCGCATAGAAGACATA |
| ZCC330 | GAACCCGGGACAAGGTGTCAGATTGCTCACCAGACTTGTC |
| ZCC331 | GAACCCGGGACAAGGTGTCAGAGATCATGCCATTCAAGCC |
| ZCC332 | GAACCCGGGACAAGGTGTCAGAATCCATGCCAACCATTGA |
| ZCC333 | GAACCCGGGACAAGGTGTCAAGCAGAGCACCAGTCTAATC |
| ZCC334 | GAACCCGGGACAAGGTGTCAGACTGCCATAGACTGCATTC |
| ZCC335 | GAACCCGGGACAAGGTGTCACAGACAGAGACAGGTGTACC |
| ZCC336 | GAACCCGGGACAAGGTGTCAGATAGCTCTGTGCATCAGGT |
| ZCC337 | GAACCCGGGACAAGGTGTCACACTCAGAGGTAGCATCAGG |
| ZCC338 | GAACCCGGGACAAGGTGTCACAGCATCAGGAAGATTCACC |
| ZCC339 | GAACCCGGGACAAGGTGTCAACAGTGTACCAATGTGCTGA |
| ZCC340 | GAACCCGGGACAAGGTGTCAACTACTGCATAAGGGAGTGC |
| ZCC341 | GAACCCGGGACAAGGTGTCACCTGCATCAGCATTATGGAG |
| ZCC342 | GAACCCGGGACAAGGTGTCAACAGCTCTGCTAAGATGGAG |
| ZCC343 | GAACCCGGGACAAGGTGTCAAGGATTCTCACAATGCCTCA |
| ZCC344 | GAACCCGGGACAAGGTGTCAAGCTGCACTGGATTCATAGT |
| ZCC345 | GAACCCGGGACAAGGTGTCAAATCTGCATGAAGGTTGAGC |
| ZCC346 | GAACCCGGGACAAGGTGTCAACATCGTAGGAAGTCTGAGC |
| ZCC347 | GAACCCGGGACAAGGTGTCACCTTGCACAGAAGAGATGGA |
| ZCC348 | GAACCCGGGACAAGGTGTCACAACTACTGACAAGCTGGGA |
| ZCC349 | GAACCCGGGACAAGGTGTCAAAGCTCTCATCAGGAGTGTG |
| ZCC350 | GAACCCGGGACAAGGTGTCAATATCCGACTCAGCTCTGTG |
| ZCC351 | GAACCCGGGACAAGGTGTCAGATAAGTGCCAACAGCCCTA |
| ZCC352 | GAACCCGGGACAAGGTGTCACCTGACTACCAATGCAAGGA |
| ZCC353 | GAACCCGGGACAAGGTGTCACTCTAGTAGGGACCAGCATG |
| ZCC354 | GAACCCGGGACAAGGTGTCACTCCATCAGAGAGAGAGCAG |
| ZCC355 | GAACCCGGGACAAGGTGTCATAGAGTATCCCACGACATGC |
| ZCC356 | GAACCCGGGACAAGGTGTCAAGTATATCACCACGGACAGC |
| ZCC357 | GAACCCGGGACAAGGTGTCATGGCAGCTAATCAACACCTA |
| ZCC358 | GAACCCGGGACAAGGTGTCAGGACCCATGAAATCCTAGCA |
| ZCC359 | GAACCCGGGACAAGGTGTCACCATCTGGGTTAAGTGCATG |
| ZCC360 | GAACCCGGGACAAGGTGTCACCATTGATATAAGGGCGCAG |
| ZCC361 | GAACCCGGGACAAGGTGTCACAGTTATAGCCATTGAGCCG |
| ZCC362 | GAACCCGGGACAAGGTGTCAAGAGCTACAGTCAGTCACAG |
| ZCC363 | GAACCCGGGACAAGGTGTCAATAGATACCGCAGCTCACAG |
| ZCC364 | GAACCCGGGACAAGGTGTCAAGATAGTCTGAACACGCTCC |
| ZCC365 | GAACCCGGGACAAGGTGTCAAACCGTCTAAACCATACGTG |
| ZCC366 | GAACCCGGGACAAGGTGTCAAAGCTACAACAATGTCTCCG |
| ZCC367 | GAACCCGGGACAAGGTGTCAACGTGTCCAAAGGGTTATAC |
| ZCC368 | GAACCCGGGACAAGGTGTCAAGCTGGTAACAGGGATTAAC |
| ZCC369 | GAACCCGGGACAAGGTGTCACGATAAGCCAGTCTAATAGC |
| ZCC370 | GAACCCGGGACAAGGTGTCAGACTTTGTAATAGTGCTGCC |
| ZCC371 | GAACCCGGGACAAGGTGTCAGATAGCAATTAGTAGGAGCC |
| ZCC372 | GAACCCGGGACAAGGTGTCAGCTCATATACCTAGCCGTAG |
| ZCC373 | GAACCCGGGACAAGGTGTCATAGCTTCACTCATAGCCATG |
| ZCC374 | GAACCCGGGACAAGGTGTCATATTTCCTAGTAGTCCCAGC |
| ZCC375 | GAACCCGGGACAAGGTGTCAAACAATCTAAACGTGCGTCC |
| ZCC376 | GAACCCGGGACAAGGTGTCAACGATTCTAAAGGCTTCACC |
| ZCC377 | GAACCCGGGACAAGGTGTCAAGCCTCACAAATCTTTACGG |
| ZCC378 | GAACCCGGGACAAGGTGTCACAAGGTCTAAGAATCATGCC |
| ZCC379 | GAACCCGGGACAAGGTGTCACATACCAGAATAGAGTCGCC |
| ZCC380 | GAACCCGGGACAAGGTGTCACGATCTACAGAGGAATACGG |
| ZCC381 | GAACCCGGGACAAGGTGTCACTTCCTTCAATCGACTTAGG |
| ZCC382 | GAACCCGGGACAAGGTGTCAGATGAGCTAATCTGCGTCAA |
| ZCC383 | GAACCCGGGACAAGGTGTCAGCGTGAGGACGTATATTCAA |
| ZCC384 | GAACCCGGGACAAGGTGTCAGGTAGCCTAATGTGTGCTCA |
| ZB’A1’ | TATGCAGTAGAGAGCTCGACGTGATTAAGTCTGCTTCGGC |
| ZB’A2’ | TATGCAGTAGAGAGCTCGACGTTCGTACTGTGGTGCCTAA |
| ZB’A3’ | TATGCAGTAGAGAGCTCGACGTTGCTAACATGGTCTCTGC |
| ZB’A4’ | TATGCAGTAGAGAGCTCGACGTTGCTCACAGTTGACTTCC |
| ZB’A5’ | TATGCAGTAGAGAGCTCGACTAGCAACGTGGTCTTGATGT |
| ZB’A6’ | TATGCAGTAGAGAGCTCGACTAGTTGGCTACGCTTGGATC |
| ZB’A7’ | TATGCAGTAGAGAGCTCGACTATAAGCTGTCATTGCGGCT |
| ZB’A8’ | TATGCAGTAGAGAGCTCGACTGAAATTGCGTCTTCTGGGA |
| ZB’A9’ | TATGCAGTAGAGAGCTCGACTGCCGGATTGTTAATTCGGA |
| ZB’A10’ | TATGCAGTAGAGAGCTCGACTGGCATGATTTCGTGGTACA |
| ZB’A11’ | TATGCAGTAGAGAGCTCGACTGGGAAGCTGTGCTCTTATC |
| ZB’A12’ | TATGCAGTAGAGAGCTCGACAACTAAACTCGAAGGGTCGG |
| ZB’A13’ | TATGCAGTAGAGAGCTCGACAATTAACGGAATCGGACGGT |
| ZB’A14’ | TATGCAGTAGAGAGCTCGACCCGCAAATTAACCGGACTTT |
| ZB’A15’ | TATGCAGTAGAGAGCTCGACTTAGTCGGTTTCTACACCCG |
| ZB’A16’ | TATGCAGTAGAGAGCTCGACCGGAAACATTAGCAACCGTT |
| ZB’A17’ | TATGCAGTAGAGAGCTCGACCGCGGTCTTACGGAGTATTA |
| ZB’A18’ | TATGCAGTAGAGAGCTCGACGGACCGCTTCGGATTAGTTA |
| ZB’A19’ | TATGCAGTAGAGAGCTCGACTGTAGGCCGTTGGTTTAACA |
| ZB’A20’ | TATGCAGTAGAGAGCTCGACAGAAACGGTCTTTGTTGTGC |
| ZB’A21’ | TATGCAGTAGAGAGCTCGACAAATTCCGCAACCTCAAGTG |
| ZB’A22’ | TATGCAGTAGAGAGCTCGACAACAGTCTAACCTACGCGAG |
| ZB’A23’ | TATGCAGTAGAGAGCTCGACAAGTCCTAACGCCTAGCAAG |
| ZB’A24’ | TATGCAGTAGAGAGCTCGACATATTTATGCAAACGGGCGG |
| ZB’A25’ | TATGCAGTAGAGAGCTCGACATTGGACAAACGTATTCGCG |
| ZB’A26’ | TATGCAGTAGAGAGCTCGACGTTCGCTGTTATTACACCGG |
| ZB’A27’ | TATGCAGTAGAGAGCTCGACTAGTGTAATTGCCTTCCGGG |
| ZB’A28’ | TATGCAGTAGAGAGCTCGACATCGGCACTGGAAGTTCTTT |
| ZB’A29’ | TATGCAGTAGAGAGCTCGACCTCTGGCCTTAACGTGACTT |
| ZB’A30’ | TATGCAGTAGAGAGCTCGACGAGGACGTTCAGACCAGTTT |
| ZB’A31’ | TATGCAGTAGAGAGCTCGACGCGAACATTGAACGACCTTT |
| ZB’A32’ | TATGCAGTAGAGAGCTCGACGCGGTAGTTCGAGAAATCCT |
| ZB’A33’ | TATGCAGTAGAGAGCTCGACGGAGGACGTTGAACTCACTT |
| ZB’A34’ | TATGCAGTAGAGAGCTCGACAGTCTTAACAAACTGCACGC |
| ZB’A35’ | TATGCAGTAGAGAGCTCGACCAAAGTAGTCGTGAACCTGC |
| ZB’A36’ | TATGCAGTAGAGAGCTCGACCGACGTAGTTACACAAGTGC |
| ZB’A37’ | TATGCAGTAGAGAGCTCGACCTAATCGAGGTCCGTGGATC |
| ZB’A38’ | TATGCAGTAGAGAGCTCGACGTCGCTTTGTGGTTAGACAC |
| ZB’A39’ | TATGCAGTAGAGAGCTCGACTACTCTGTTGAAGGTTCGGC |
| ZB’A40’ | TATGCAGTAGAGAGCTCGACAATTCATCCATCGACCGGAG |
| ZB’A41’ | TATGCAGTAGAGAGCTCGACCCCAACATTAACGCAGAGTG |
| ZB’A42’ | TATGCAGTAGAGAGCTCGACCGGCAAACTTAGAGAAGCTG |
| ZB’A43’ | TATGCAGTAGAGAGCTCGACACATCACCTCGGACGGTATA |
| ZB’A44’ | TATGCAGTAGAGAGCTCGACACGTACAGTATAGCCTCCGA |
| ZB’A45’ | TATGCAGTAGAGAGCTCGACACTACATCGACTCCGAAGGA |
| ZB’A46’ | TATGCAGTAGAGAGCTCGACAGCCCACGTACTCGTATAGA |
| ZB’A47’ | TATGCAGTAGAGAGCTCGACCGTCAAACTCTGTACCGAGA |
| ZB’A48’ | TATGCAGTAGAGAGCTCGACCTTTAACTCCTCTGCGTGGA |
| ZB’A49’ | TATGCAGTAGAGAGCTCGACGGAGCCCTTCGCTACAATTA |
| ZB’A50’ | TATGCAGTAGAGAGCTCGACTGAATGTTTACTTCGCGGGA |
| ZB’A51’ | TATGCAGTAGAGAGCTCGACGGCAACGGTTCATTACTGTG |
| ZB’A52’ | TATGCAGTAGAGAGCTCGACGTACGCCATGTTCGGTAATG |
| ZB’A53’ | TATGCAGTAGAGAGCTCGACGTCAATTCACTTCGTCGTGG |
| ZB’A54’ | TATGCAGTAGAGAGCTCGACGTCCTCAGTTCTATCCGTGG |
| ZB’A55’ | TATGCAGTAGAGAGCTCGACGTCTTGACTTAATGTCGCGG |
| ZB’A56’ | TATGCAGTAGAGAGCTCGACGTTCTTCATTCTACGCACGG |
| ZB’A57’ | TATGCAGTAGAGAGCTCGACTAAAGTTGCATTCAAGGCCG |
| ZB’A58’ | TATGCAGTAGAGAGCTCGACTAACAGGGTTCTTCGCTCTG |
| ZB’A59’ | TATGCAGTAGAGAGCTCGACTAAGCCTGTTCTCGATTCGG |
| ZB’A60’ | TATGCAGTAGAGAGCTCGACTTCTCCACTTTGCGGATAGG |
| ZB’A61’ | TATGCAGTAGAGAGCTCGACTTTAGTGCTTTGCGAGAACG |
| ZB’A62’ | TATGCAGTAGAGAGCTCGACTTTCATACTTTGCGCGAAGG |
| ZB’A63’ | TATGCAGTAGAGAGCTCGACAAACATACTCAGCGAGGCTT |
| ZB’A64’ | TATGCAGTAGAGAGCTCGACAACTCACTCGACGCGAATAT |
| ZB’A65’ | TATGCAGTAGAGAGCTCGACAGATCAATGACGCGAGGTTT |
| ZB’A66’ | TATGCAGTAGAGAGCTCGACAGCTAACCTGAATCCGCATT |
| ZB’A67’ | TATGCAGTAGAGAGCTCGACAGGCGGCATGAGAACTAATT |
| ZB’A68’ | TATGCAGTAGAGAGCTCGACAGTCTGAGGATCGTAAGCCT |
| ZB’A69’ | TATGCAGTAGAGAGCTCGACCCATCGGATTACACACGAGT |
| ZB’A70’ | TATGCAGTAGAGAGCTCGACCCCGAACATCTCGAATGAGT |
| ZB’A71’ | TATGCAGTAGAGAGCTCGACCCTAACGCTGTAGCTGAAGT |
| ZB’A72’ | TATGCAGTAGAGAGCTCGACCTCAAGTATTAGAGCGCGGT |
| ZB’A73’ | TATGCAGTAGAGAGCTCGACGAGCGACGTTCACTAAGTCT |
| ZB’A74’ | TATGCAGTAGAGAGCTCGACGTAGAACATTCGGTGGCTCT |
| ZB’A75’ | TATGCAGTAGAGAGCTCGACCCCTAGAGTCTGCTACGAGA |
| ZB’A76’ | TATGCAGTAGAGAGCTCGACGCTCCGGTTCGATGAGATTA |
| ZB’A77’ | TATGCAGTAGAGAGCTCGACGGCAAAGGTGTGCTACTCTA |
| ZB’A78’ | TATGCAGTAGAGAGCTCGACGTCCAGTTCGTGTATGGTCA |
| ZB’A79’ | TATGCAGTAGAGAGCTCGACATAGTGACGCTGAAGTAGCC |
| ZB’A80’ | TATGCAGTAGAGAGCTCGACCGTGACCATTAAGTCGATGC |
| ZB’A81’ | TATGCAGTAGAGAGCTCGACGAAAGACTTAGCGTGACTGC |
| ZB’A82’ | TATGCAGTAGAGAGCTCGACGACCATGTGCGTAGTGTTTC |
| ZB’A83’ | TATGCAGTAGAGAGCTCGACGTGTTATGTTATCCGAGCGC |
| ZB’A84’ | TATGCAGTAGAGAGCTCGACGTTCATTCTTAGATGGCGGC |
| ZB’A85’ | TATGCAGTAGAGAGCTCGACTAGGCTGTTGGACGATCTTC |
| ZB’A86’ | TATGCAGTAGAGAGCTCGACGTTAGTGGTTCTCATGCAGC |
| ZB’A87’ | TATGCAGTAGAGAGCTCGACTATGGGTCTTGCTGATACGC |
| ZB’A88’ | TATGCAGTAGAGAGCTCGACTGGTCGTGTTTCAGATGACA |
| ZB’A89’ | TATGCAGTAGAGAGCTCGACAGTAAATGCCATCATCGCCT |
| ZB’A90’ | TATGCAGTAGAGAGCTCGACTGAAGCAATTTAGGCATGGC |
| ZB’A91’ | TATGCAGTAGAGAGCTCGACTGAAGCTGTCTGTTGATGCA |
| ZB’A92’ | TATGCAGTAGAGAGCTCGACACATACAATCGCTGAAGCCT |
| ZB’A93’ | TATGCAGTAGAGAGCTCGACATGTATCACACTCTCGGCTG |
| ZB’A94’ | TATGCAGTAGAGAGCTCGACCAGCTCGTGAGATGACGTAT |
| ZB’A95’ | TATGCAGTAGAGAGCTCGACTATTGCAGTTCTCTGACCGG |
| ZB’A96’ | TATGCAGTAGAGAGCTCGACCAGCCGTCTGCATCAGTATA |
| ZB’A97’ | TATGCAGTAGAGAGCTCGACGGCTATGATTCGCAATGCTT |
| ZB’A98’ | TATGCAGTAGAGAGCTCGACTAATGGCGTTGATTCGATGC |
| ZB’A99’ | TATGCAGTAGAGAGCTCGACATTGCTGACAAGTGAGGCTT |
| ZB’A100’ | TATGCAGTAGAGAGCTCGACATTTCATCATAATCGCGCCG |
| ZB’A101’ | TATGCAGTAGAGAGCTCGACCTCAATGTGCAGTGGTATGC |
| ZB’A102’ | TATGCAGTAGAGAGCTCGACCAGTCCGTGATCCTCGATAG |
| ZB’A103’ | TATGCAGTAGAGAGCTCGACGCACGAGATGTGATCGAATC |
| ZB’A104’ | TATGCAGTAGAGAGCTCGACACGTAGATGAGGAGCACTCT |
| ZB’A105’ | TATGCAGTAGAGAGCTCGACGCAGAACGTGGAATGTCATC |
| ZB’A106’ | TATGCAGTAGAGAGCTCGACGCTACCTGTTCCATCAGTGT |
| ZB’A107’ | TATGCAGTAGAGAGCTCGACGCTACTCGTGTCTGCTAAGT |
| ZB’A108’ | TATGCAGTAGAGAGCTCGACTACGCGAGTTGGACATGATT |
| ZB’A109’ | TATGCAGTAGAGAGCTCGACTGTGATCTTGTGGCATGACA |
| ZB’A110’ | TATGCAGTAGAGAGCTCGACGTACTGTGTCTTAGCTGGCA |
| ZB’A111’ | TATGCAGTAGAGAGCTCGACTACTCCATTGCCTCGATGTG |
| ZB’A112’ | TATGCAGTAGAGAGCTCGACACTCATCGTAGCCACTGAAG |
| ZB’A113’ | TATGCAGTAGAGAGCTCGACACTCGATATGAGCAGCAGTC |
| ZB’A114’ | TATGCAGTAGAGAGCTCGACCAAGGTATGATGCGAACTGC |
| ZB’A115’ | TATGCAGTAGAGAGCTCGACCGATAGCTTACACGTCATGC |
| ZB’A116’ | TATGCAGTAGAGAGCTCGACTCATTAAATTGATCCCGCGC |
| ZB’A117’ | TATGCAGTAGAGAGCTCGACTCATTGCGTGTATGTGGACA |
| ZB’A118’ | TATGCAGTAGAGAGCTCGACCACAAACTGATCGCAGATGG |
| ZB’A119’ | TATGCAGTAGAGAGCTCGACCGCAAATTACACTCGATGCT |
| ZB’A120’ | TATGCAGTAGAGAGCTCGACATCAGATCGACTCGGTAGCT |
| ZB’A121’ | TATGCAGTAGAGAGCTCGACCGCACAGCTCCTAAGTGATA |
| ZB’A122’ | TATGCAGTAGAGAGCTCGACCTGACGGTTAGAGTGCATCT |
| ZB’A123’ | TATGCAGTAGAGAGCTCGACGCATACATTCGCACGCATTA |
| ZB’A124’ | TATGCAGTAGAGAGCTCGACTCCATACTTGGTAGCGTGTG |
| ZB’A125’ | TATGCAGTAGAGAGCTCGACTCCATTACTTGCTCTAGCGG |
| ZB’A126’ | TATGCAGTAGAGAGCTCGACTGCATTAAGTTGCTTGGCAG |
| ZB’A127’ | TATGCAGTAGAGAGCTCGACATCATGCAGAGCTGACTGAG |
| ZB’A128’ | TATGCAGTAGAGAGCTCGACCATGCGATGTACCTCCGTAT |
| ZB’A129’ | TATGCAGTAGAGAGCTCGACGCATGGCGTTAGTGCATATT |
| ZB’A130’ | TATGCAGTAGAGAGCTCGACTTCGTGGATTTGCATGAAGC |
| ZB’A131’ | TATGCAGTAGAGAGCTCGACGAGTGAGCTTCAATGCCATG |
| ZB’A132’ | TATGCAGTAGAGAGCTCGACTAGTAACATTGGCGCACTGT |
| ZB’A133’ | TATGCAGTAGAGAGCTCGACGTGAGCCTTGATCGCAATTT |
| ZB’A134’ | TATGCAGTAGAGAGCTCGACGTGATGTTGCACGATGTCTC |
| ZB’A135’ | TATGCAGTAGAGAGCTCGACTAGTCTTCTTGACGTGCAGG |
| ZB’A136’ | TATGCAGTAGAGAGCTCGACAAATCCTGGCACACAGTCAT |
| ZB’A137’ | TATGCAGTAGAGAGCTCGACGCCAGAAATGTTCAGCACTC |
| ZB’A138’ | TATGCAGTAGAGAGCTCGACAGAGCTAATGAGAGCATCGC |
| ZB’A139’ | TATGCAGTAGAGAGCTCGACCACAGATATAGCCGTGCTCA |
| ZB’A140’ | TATGCAGTAGAGAGCTCGACCTGCCATATCGCTTAGCATG |
| ZB’A141’ | TATGCAGTAGAGAGCTCGACGCATCGACTCTTCATTTGGG |
| ZB’A142’ | TATGCAGTAGAGAGCTCGACGCTGCTGATGTCTGGCTATA |
| ZB’A143’ | TATGCAGTAGAGAGCTCGACTAGCGTCTTGGAGCTGATTC |
| ZB’A144’ | TATGCAGTAGAGAGCTCGACTGCCCATATTTCAACATGCG |
| ZB’A145’ | TATGCAGTAGAGAGCTCGACCCAATAGTGATGAGTCGCCT |
| ZB’A146’ | TATGCAGTAGAGAGCTCGACCCAATCGTGATCTAGCGTGA |
| ZB’A147’ | TATGCAGTAGAGAGCTCGACCTGTCCTGCATGGTGTAGAA |
| ZB’A148’ | TATGCAGTAGAGAGCTCGACGACGCTGCTTCAATCAGATG |
| ZB’A149’ | TATGCAGTAGAGAGCTCGACTCGGACCTTGTCGATATGTG |
| ZB’A150’ | TATGCAGTAGAGAGCTCGACAAGCCAGCTAGACTCCATTG |
| ZB’A151’ | TATGCAGTAGAGAGCTCGACTTAATGCCTTTCGCTCAAGC |
| ZB’A152’ | TATGCAGTAGAGAGCTCGACTTAATGGATTTCGCGTGAGC |
| ZB’A153’ | TATGCAGTAGAGAGCTCGACCGATACAATGGCATGGCATC |
| ZB’A154’ | TATGCAGTAGAGAGCTCGACTAGAAGCTGGTCTTGACTGC |
| ZB’A155’ | TATGCAGTAGAGAGCTCGACTGATTATTGTCCTGCCGGAG |
| ZB’A156’ | TATGCAGTAGAGAGCTCGACTGATTGCTTGTGAATAGCGC |
| ZB’A157’ | TATGCAGTAGAGAGCTCGACGATACTCGTTCAGCACGTCT |
| ZB’A158’ | TATGCAGTAGAGAGCTCGACCACCGAGATGCTAAGAGCTT |
| ZB’A159’ | TATGCAGTAGAGAGCTCGACCTCTGTGAACTGTCATCGGT |
| ZB’A160’ | TATGCAGTAGAGAGCTCGACCACTACCTGAGGCTCGTAAG |
| ZB’A161’ | TATGCAGTAGAGAGCTCGACCACTACGATGCGATCCAGTA |
| ZB’A162’ | TATGCAGTAGAGAGCTCGACCACTAGAATGCGACGTAGGT |
| ZB’A163’ | TATGCAGTAGAGAGCTCGACCGTCCAGTTAGAGACATGCT |
| ZB’A164’ | TATGCAGTAGAGAGCTCGACCTGCCGTATGCTTAGAGGTT |
| ZB’A165’ | TATGCAGTAGAGAGCTCGACTTACACCGTTTGACAGCTCT |
| ZB’A166’ | TATGCAGTAGAGAGCTCGACTCCGAGGATTTAACGCATGT |
| ZB’A167’ | TATGCAGTAGAGAGCTCGACTGTATGAGTTTCCAGTCGGC |
| ZB’A168’ | TATGCAGTAGAGAGCTCGACAGTTATGACAACGGTGGCAT |
| ZB’A169’ | TATGCAGTAGAGAGCTCGACGACACCCGTTAGGTTTCTCA |
| ZB’A170’ | TATGCAGTAGAGAGCTCGACCGATGATCTGACTGGAGCTT |
| ZB’A171’ | TATGCAGTAGAGAGCTCGACCCGCAGCTTAATCGACATTT |
| ZB’A172’ | TATGCAGTAGAGAGCTCGACGATCGCTTCGAGGGTATTCA |
| ZB’A173’ | TATGCAGTAGAGAGCTCGACGTGCGCTATTCTGCATATCG |
| ZB’A174’ | TATGCAGTAGAGAGCTCGACATTGCATACAGGTCTCCGTC |
| ZB’A175’ | TATGCAGTAGAGAGCTCGACTCTTATATTCTGGCGACGGC |
| ZB’A176’ | TATGCAGTAGAGAGCTCGACCAGCCACATGCCGATATAGA |
| ZB’A177’ | TATGCAGTAGAGAGCTCGACCTAATGCAGTCAGTGGTCGA |
| ZB’A178’ | TATGCAGTAGAGAGCTCGACGAGCGTGATGGACTCTACTC |
| ZB’A179’ | TATGCAGTAGAGAGCTCGACCCGAGACATGGAGAGTCATC |
| ZB’A180’ | TATGCAGTAGAGAGCTCGACCGTAGTGTCGCAGTCTGTAT |
| ZB’A181’ | TATGCAGTAGAGAGCTCGACTATGTTTGTCACTCCGGGAC |
| ZB’A182’ | TATGCAGTAGAGAGCTCGACCACCACAGTGACTATGCAGT |
| ZB’A183’ | TATGCAGTAGAGAGCTCGACTCGGGAATTGTGAAGCATCT |
| ZB’A184’ | TATGCAGTAGAGAGCTCGACTTGTTAGATTTGCACGCAGG |
| ZB’A185’ | TATGCAGTAGAGAGCTCGACTTTAATGGTTTGCAGCAGGC |
| ZB’A186’ | TATGCAGTAGAGAGCTCGACCGGGCACATTCAACATCATT |
| ZB’A187’ | TATGCAGTAGAGAGCTCGACTGGAGATCGTTGCTTGTACC |
| ZB’A188’ | TATGCAGTAGAGAGCTCGACTCTAACATTGTCCGTCTGGC |
| ZB’A189’ | TATGCAGTAGAGAGCTCGACTCCTAATCTTGGATGCGTGG |
| ZB’A190’ | TATGCAGTAGAGAGCTCGACAAGGTACATAGCTCGCAGTC |
| ZB’A191’ | TATGCAGTAGAGAGCTCGACGCTCAATGTCTCTCATGTGC |
| ZB’A192’ | TATGCAGTAGAGAGCTCGACAGCAGAAGTGATAACGCTGT |
| ZB’A193’ | TATGCAGTAGAGAGCTCGACCCGAAACGTCGATTACCAAG |
| ZB’A194’ | TATGCAGTAGAGAGCTCGACCCGAATCATCTCGTCAAGGT |
| ZB’A195’ | TATGCAGTAGAGAGCTCGACCCAGCGTATCTGCATAGTCA |
| ZB’A196’ | TATGCAGTAGAGAGCTCGACCTTCGCTCCCTGTAGATGAG |
| ZB’A197’ | TATGCAGTAGAGAGCTCGACTCTACAAATTGCATTGGGCG |
| ZB’A198’ | TATGCAGTAGAGAGCTCGACCAGACACGTATCTCGCAGTA |
| ZB’A199’ | TATGCAGTAGAGAGCTCGACCGGTATCTGCTCCGTCATAG |
| ZB’A200’ | TATGCAGTAGAGAGCTCGACCGGTATTCCGACAGCTTAGT |
| ZB’A201’ | TATGCAGTAGAGAGCTCGACCGGTCAATGTCAGTACGGAT |
| ZB’A202’ | TATGCAGTAGAGAGCTCGACCGGTGCTATACGGTGTTACT |
| ZB’A203’ | TATGCAGTAGAGAGCTCGACCGGTTCCTAAGCTCGTATGT |
| ZB’A204’ | TATGCAGTAGAGAGCTCGACCGTACTGGTAGGCTATCGTC |
| ZB’A205’ | TATGCAGTAGAGAGCTCGACCGTCATAGTCGTGCTGAGTA |
| ZB’A206’ | TATGCAGTAGAGAGCTCGACGATCCGTTCGACCCAGTTAT |
| ZB’A207’ | TATGCAGTAGAGAGCTCGACGATCCTACTGTCGGTCTGTG |
| ZB’A208’ | TATGCAGTAGAGAGCTCGACGATCTCGTTACGTCGGTGTA |
| ZB’A209’ | TATGCAGTAGAGAGCTCGACACCAAGAGTGACATCAGCTC |
| ZB’A210’ | TATGCAGTAGAGAGCTCGACAGTGAAGATGACTGAGCACC |
| ZB’A211’ | TATGCAGTAGAGAGCTCGACAGTGATCGCACCTTATCTCG |
| ZB’A212’ | TATGCAGTAGAGAGCTCGACCCTCGACATGCGTACTATGA |
| ZB’A213’ | TATGCAGTAGAGAGCTCGACCTCTGACTGCTGGAGTATCG |
| ZB’A214’ | TATGCAGTAGAGAGCTCGACCTGAACTGTACTCGGTGGTT |
| ZB’A215’ | TATGCAGTAGAGAGCTCGACCTGCATAGTTAGACGGTCGT |
| ZB’A216’ | TATGCAGTAGAGAGCTCGACCTTCTGGGCATGGTCGAATA |
| ZB’A217’ | TATGCAGTAGAGAGCTCGACGAATAAGCTCTCGGTGTCGT |
| ZB’A218’ | TATGCAGTAGAGAGCTCGACGAATAGTTACGCTGCCTTGC |
| ZB’A219’ | TATGCAGTAGAGAGCTCGACGCATTCTAAACAGTGTGCGT |
| ZB’A220’ | TATGCAGTAGAGAGCTCGACGCATTGTCAAACCTGTTGGT |
| ZB’A221’ | TATGCAGTAGAGAGCTCGACGCGTTCTGCGAAATGGTTAT |
| ZB’A222’ | TATGCAGTAGAGAGCTCGACGCTCACTTCCGCTAGATTGA |
| ZB’A223’ | TATGCAGTAGAGAGCTCGACGCTTGCGAAGAATTACGTGT |
| ZB’A224’ | TATGCAGTAGAGAGCTCGACGGCCGTCATGTCTATTCAGT |
| ZB’A225’ | TATGCAGTAGAGAGCTCGACGGCGAAGTTCCTCTAGTGTT |
| ZB’A226’ | TATGCAGTAGAGAGCTCGACGGCGATCTTGGACAAAGTTC |
| ZB’A227’ | TATGCAGTAGAGAGCTCGACGGCTTCACTTCGAGTTAGGT |
| ZB’A228’ | TATGCAGTAGAGAGCTCGACGGGCCATCTGTGATTATCGT |
| ZB’A229’ | TATGCAGTAGAGAGCTCGACGTAGTGTTCAGTCTGTGCCA |
| ZB’A230’ | TATGCAGTAGAGAGCTCGACGTCGTCTGTATTACTCGCCA |
| ZB’A231’ | TATGCAGTAGAGAGCTCGACTAACAATTCATTAGCGGCGC |
| ZB’A232’ | TATGCAGTAGAGAGCTCGACTCACACTTGCTTCGTCTGAA |
| ZB’A233’ | TATGCAGTAGAGAGCTCGACTCACGAGTTGCTGTTGATGA |
| ZB’A234’ | TATGCAGTAGAGAGCTCGACTCACGTAGTGTTCTCTGAGC |
| ZB’A235’ | TATGCAGTAGAGAGCTCGACTCACTAGCTTGTCAACGCTT |
| ZB’A236’ | TATGCAGTAGAGAGCTCGACTCATTTACACGAGTTGGGCT |
| ZB’A237’ | TATGCAGTAGAGAGCTCGACTCCCTCGTTGTATGCTAGGA |
| ZB’A238’ | TATGCAGTAGAGAGCTCGACTCCGTTTAGAAGTCTTGGGC |
| ZB’A239’ | TATGCAGTAGAGAGCTCGACCGGACCCGTTAATCACGTAT |
| ZB’A240’ | TATGCAGTAGAGAGCTCGACCTAAGGACTGTGGTTTCCGT |
| ZB’A241’ | TATGCAGTAGAGAGCTCGACCTAGGTCTTCCACACGGTTT |
| ZB’A242’ | TATGCAGTAGAGAGCTCGACTCGCTAAGTTGGGTGTTACC |
| ZB’A243’ | TATGCAGTAGAGAGCTCGACGAAGTTTCGACAACCTGGTG |
| ZB’A244’ | TATGCAGTAGAGAGCTCGACAAACTCGATAAGCGTAGCGT |
| ZB’A245’ | TATGCAGTAGAGAGCTCGACAATAGCTCGATACCGGGAGT |
| ZB’A246’ | TATGCAGTAGAGAGCTCGACAATTATCGGAACTCGTCGCT |
| ZB’A247’ | TATGCAGTAGAGAGCTCGACACCAAGTGTCACAACTCGTT |
| ZB’A248’ | TATGCAGTAGAGAGCTCGACCAAACACGTAGTCTGAGGGT |
| ZB’A249’ | TATGCAGTAGAGAGCTCGACCAACCAGTGGACGGTAATCT |
| ZB’A250’ | TATGCAGTAGAGAGCTCGACCGACGCATTACGACCAATTT |
| ZB’A251’ | TATGCAGTAGAGAGCTCGACCGCGCCCATTCAAAGTAATT |
| ZB’A252’ | TATGCAGTAGAGAGCTCGACCGGTATCCTCGTCGCTAAAT |
| ZB’A253’ | TATGCAGTAGAGAGCTCGACGGTGCCCGTTCTAAGAGATT |
| ZB’A254’ | TATGCAGTAGAGAGCTCGACGTCACCGGGTATGTTCAGTT |
| ZB’A255’ | TATGCAGTAGAGAGCTCGACTCCGAACCTTTAAGCACTGT |
| ZB’A256’ | TATGCAGTAGAGAGCTCGACTTACCACGTTTGACCACTGT |
| ZB’A257’ | TATGCAGTAGAGAGCTCGACAAGTCGGCTAGACGGCTATA |
| ZB’A258’ | TATGCAGTAGAGAGCTCGACGAAACCCGTTACTGACATGC |
| ZB’A259’ | TATGCAGTAGAGAGCTCGACGACACACGTCTTGGTTGTTC |
| ZB’A260’ | TATGCAGTAGAGAGCTCGACGACTTCGTGAAAGGTGCTTC |
| ZB’A261’ | TATGCAGTAGAGAGCTCGACTCGGTTAGTTGGTGACATCC |
| ZB’A262’ | TATGCAGTAGAGAGCTCGACTGTCTTTATTGAAACCGCGC |
| ZB’A263’ | TATGCAGTAGAGAGCTCGACTTGGACGATTTGGAATTGCC |
| ZB’A264’ | TATGCAGTAGAGAGCTCGACACGGCCCTCTAAGCAATATG |
| ZB’A265’ | TATGCAGTAGAGAGCTCGACACTCGCAATCTCGAACTAGG |
| ZB’A266’ | TATGCAGTAGAGAGCTCGACATACAATGAGGCGTACTCGG |
| ZB’A267’ | TATGCAGTAGAGAGCTCGACATTCGACGCCATCGTAGTAG |
| ZB’A268’ | TATGCAGTAGAGAGCTCGACCAACGTAGTGTCAATCACGG |
| ZB’A269’ | TATGCAGTAGAGAGCTCGACGCGTTCGAGTCGTCTTAATG |
| ZB’A270’ | TATGCAGTAGAGAGCTCGACGCTTGCTAACACTTCTTGGG |
| ZB’A271’ | TATGCAGTAGAGAGCTCGACCGACCGAATGGGAAGTCATA |
| ZB’A272’ | TATGCAGTAGAGAGCTCGACTTTACGGCTTTGGTAGAGCA |
| ZB’A273’ | TATGCAGTAGAGAGCTCGACAACAGGTGAATAGACGGCTC |
| ZB’A274’ | TATGCAGTAGAGAGCTCGACTATACATGGTTCCTTCGCCG |
| ZB’A275’ | TATGCAGTAGAGAGCTCGACTATACTACTTGCGCTTCCGG |
| ZB’A276’ | TATGCAGTAGAGAGCTCGACTATTCCATTGGGCGTACTGG |
| ZB’A277’ | TATGCAGTAGAGAGCTCGACTCCAGGCGTTTAAGACTCTG |
| ZB’A278’ | TATGCAGTAGAGAGCTCGACTCTCGTTCTTGTCAACCAGG |
| ZB’A279’ | TATGCAGTAGAGAGCTCGACACGATACATGCGAACCAGTT |
| ZB’A280’ | TATGCAGTAGAGAGCTCGACATACTAGGTACGCTGAGCCT |
| ZB’A281’ | TATGCAGTAGAGAGCTCGACATTGCGAACAAGTCTAGGCT |
| ZB’A282’ | TATGCAGTAGAGAGCTCGACCGCGAGACTTAGACACTCTT |
| ZB’A283’ | TATGCAGTAGAGAGCTCGACCGCTGACCTGTCTAACGTAT |
| ZB’A284’ | TATGCAGTAGAGAGCTCGACCGTCGTCATGGCTACTTAGT |
| ZB’A285’ | TATGCAGTAGAGAGCTCGACCGTTACTGAAGCTGAGGTCT |
| ZB’A286’ | TATGCAGTAGAGAGCTCGACGCCAGTACGGGTTATCATGT |
| ZB’A287’ | TATGCAGTAGAGAGCTCGACGCCGATCCTTCAGTCGTATT |
| ZB’A288’ | TATGCAGTAGAGAGCTCGACGCTCCCGCTTCATAGTAGTT |
| ZB’A289’ | TATGCAGTAGAGAGCTCGACTAGGAGCGTTGGCAAATCTT |
| ZB’A290’ | TATGCAGTAGAGAGCTCGACTATAACCATTGGCCTGCGTT |
| ZB’A291’ | TATGCAGTAGAGAGCTCGACTCGAAGCGTTTAGACCATGT |
| ZB’A292’ | TATGCAGTAGAGAGCTCGACTGCGAAGGTTTGACAGACTT |
| ZB’A293’ | TATGCAGTAGAGAGCTCGACACACTAGATCCCGCGAGATA |
| ZB’A294’ | TATGCAGTAGAGAGCTCGACACCGTACTGAGACATCCTGA |
| ZB’A295’ | TATGCAGTAGAGAGCTCGACCACATACGTGTACCCGATGA |
| ZB’A296’ | TATGCAGTAGAGAGCTCGACCAGCTTACTCGCTCAAGGTA |
| ZB’A297’ | TATGCAGTAGAGAGCTCGACCGTGTAGGTCGCTATGCTTA |
| ZB’A298’ | TATGCAGTAGAGAGCTCGACCTGCCACTTACGGTGATGTA |
| ZB’A299’ | TATGCAGTAGAGAGCTCGACGAACACTGTAGTCGGTGCTA |
| ZB’A300’ | TATGCAGTAGAGAGCTCGACGACACGCTTATGACCCTGTA |
| ZB’A301’ | TATGCAGTAGAGAGCTCGACGCCAGTATTCCCAGCGATTA |
| ZB’A302’ | TATGCAGTAGAGAGCTCGACTGGATTCGTTTATAGCGCGA |
| ZB’A303’ | TATGCAGTAGAGAGCTCGACAACACGGATCTAGCACAGTC |
| ZB’A304’ | TATGCAGTAGAGAGCTCGACAACAGCTCAGAGTAGGTTGC |
| ZB’A305’ | TATGCAGTAGAGAGCTCGACCAGTAGTCTGGGACGACATC |
| ZB’A306’ | TATGCAGTAGAGAGCTCGACCATGCAAGTGGGTAACGATC |
| ZB’A307’ | TATGCAGTAGAGAGCTCGACCGACATAGTGGTCAGGACTC |
| ZB’A308’ | TATGCAGTAGAGAGCTCGACGCGTTACTTAGCTGTGTGAC |
| ZB’A309’ | TATGCAGTAGAGAGCTCGACGGCGTAAATGTGTCCATAGC |
| ZB’A310’ | TATGCAGTAGAGAGCTCGACGTCGGGCTTGATAGTCTCTC |
| ZB’A311’ | TATGCAGTAGAGAGCTCGACGTTCCACTTGAGTCATGTGC |
| ZB’A312’ | TATGCAGTAGAGAGCTCGACTATGGACTTGCTCGTTAGGC |
| ZB’A313’ | TATGCAGTAGAGAGCTCGACAGCTGTCTCACTCCGTAGAT |
| ZB’A314’ | TATGCAGTAGAGAGCTCGACGATGCGAATGTGCGATACTC |
| ZB’A315’ | TATGCAGTAGAGAGCTCGACGTTGCTCTTATGTCAGACGC |
| ZB’A316’ | TATGCAGTAGAGAGCTCGACATGCGCCTCACTACTGAATT |
| ZB’A317’ | TATGCAGTAGAGAGCTCGACGAGCATCATGTGTCAGCATC |
| ZB’A318’ | TATGCAGTAGAGAGCTCGACACAATGTCGCAGCCTATCAT |
| ZB’A319’ | TATGCAGTAGAGAGCTCGACGCAAATGCTTAGTGCGTCTT |
| ZB’A320’ | TATGCAGTAGAGAGCTCGACAGTCAAGCTAGATGCCGATC |
| ZB’A321’ | TATGCAGTAGAGAGCTCGACATGTGCAGACTGAGCTGAAT |
| ZB’A322’ | TATGCAGTAGAGAGCTCGACGGCTTGAGCAGTATTCTTGC |
| ZB’A323’ | TATGCAGTAGAGAGCTCGACGGCTTGCAGTAACTTCGATG |
| ZB’A324’ | TATGCAGTAGAGAGCTCGACGAGGCAGTTCAATCGAGCTA |
| ZB’A325’ | TATGCAGTAGAGAGCTCGACCTATGTAATGAGCTGTGCGC |
| ZB’A326’ | TATGCAGTAGAGAGCTCGACTCACGCTCTTGTAGCACATT |
| ZB’A327’ | TATGCAGTAGAGAGCTCGACTGACGATTTATGTGATGCGC |
| ZB’A328’ | TATGCAGTAGAGAGCTCGACATACATCATGCACGCCTGAA |
| ZB’A329’ | TATGCAGTAGAGAGCTCGACATACATGAGCATAGCTGCGT |
| ZB’A330’ | TATGCAGTAGAGAGCTCGACCAAATGGATACAGATGCGGC |
| ZB’A331’ | TATGCAGTAGAGAGCTCGACGGTCTGCATTCGCTGATATG |
| ZB’A332’ | TATGCAGTAGAGAGCTCGACTACATCGCTTGCATGAGTGT |
| ZB’A333’ | TATGCAGTAGAGAGCTCGACTGAGCGTATTTGCTGTCAGA |
| ZB’A334’ | TATGCAGTAGAGAGCTCGACTGAGCTACTTTCAACGCTGT |
| ZB’A335’ | TATGCAGTAGAGAGCTCGACCAACGCATGAGGATGATGAC |
| ZB’A336’ | TATGCAGTAGAGAGCTCGACCAACGCTGTCCCAGATGATA |
| ZB’A337’ | TATGCAGTAGAGAGCTCGACTCAGCGGATTGTACGTCATT |
| ZB’A338’ | TATGCAGTAGAGAGCTCGACGCACTTCATGCACTGTTCAT |
| ZB’A339’ | TATGCAGTAGAGAGCTCGACTGAGGATCTTTGTCCGTAGC |
| ZB’A340’ | TATGCAGTAGAGAGCTCGACTGTGGAGATTTCTAGTCGCC |
| ZB’A341’ | TATGCAGTAGAGAGCTCGACCTCGCACCTGCTATATGTGA |
| ZB’A342’ | TATGCAGTAGAGAGCTCGACGCAGCGTCTCTCTATGTGAT |
| ZB’A343’ | TATGCAGTAGAGAGCTCGACCGATGAATGCTGAGGCTCTA |
| ZB’A344’ | TATGCAGTAGAGAGCTCGACTCCAACTCTGTGTGTGTAGC |
| ZB’A345’ | TATGCAGTAGAGAGCTCGACCATCGAACTGCTACGACTGA |
| ZB’A346’ | TATGCAGTAGAGAGCTCGACTAGACTCGTGTTGATCCTGC |
| ZB’A347’ | TATGCAGTAGAGAGCTCGACGCTCTTCATGGTCATTCAGC |
| ZB’A348’ | TATGCAGTAGAGAGCTCGACGTCCTGTTAGTGCTGTGACA |
| ZB’A349’ | TATGCAGTAGAGAGCTCGACTAGATGACTGTTCTGCCTGC |
| ZB’A350’ | TATGCAGTAGAGAGCTCGACTCCATCGTTGGTACTGATCG |
| ZB’A351’ | TATGCAGTAGAGAGCTCGACTAGCAGAATTGCGCTCTCTT |
| ZB’A352’ | TATGCAGTAGAGAGCTCGACTCCCAGAATTGTCAGCGATT |
| ZB’A353’ | TATGCAGTAGAGAGCTCGACTGCCACACTTTCAGCATAGT |
| ZB’A354’ | TATGCAGTAGAGAGCTCGACTCCGCACATTTAATAAGCGC |
| ZB’A355’ | TATGCAGTAGAGAGCTCGACTGCCACTCTGTTGTATGGAG |
| ZB’A356’ | TATGCAGTAGAGAGCTCGACCATGAGAGTGCGGCTAACTA |
| ZB’A357’ | TATGCAGTAGAGAGCTCGACTCGAAGAATTGGGTGCATCT |
| ZB’A358’ | TATGCAGTAGAGAGCTCGACGCATGTTAGAGCATCTTGCC |
| ZB’A359’ | TATGCAGTAGAGAGCTCGACCGCGTATTCACAGCACTTAG |
| ZB’A360’ | TATGCAGTAGAGAGCTCGACCACCGCAATGCTATCAGATG |
| ZB’A361’ | TATGCAGTAGAGAGCTCGACGACCGAAATGTGCATGACTC |
| ZB’A362’ | TATGCAGTAGAGAGCTCGACCATTATGAGCACTGCGCTTT |
| ZB’A363’ | TATGCAGTAGAGAGCTCGACCGCTTAAATCATCTGCGTGT |
| ZB’A364’ | TATGCAGTAGAGAGCTCGACCGCCTCGATGCTAGTATCAT |
| ZB’A365’ | TATGCAGTAGAGAGCTCGACCCGAACGGGTTTAAGTGTTT |
| ZB’A366’ | TATGCAGTAGAGAGCTCGACCGGCGGTCATTTATAGTCGT |
| ZB’A367’ | TATGCAGTAGAGAGCTCGACCGCGGCTGGTGTCTTACTAT |
| ZB’A368’ | TATGCAGTAGAGAGCTCGACGGGTCAATGATTAACATGCG |
| ZB’A369’ | TATGCAGTAGAGAGCTCGACTACTATCTGCGTCTCTGGCG |
| ZB’A370’ | TATGCAGTAGAGAGCTCGACTTATCTCGACTTCAATGGCG |
| ZB’A371’ | TATGCAGTAGAGAGCTCGACCGGGACGAGATATTCAATTC |
| ZB’A372’ | TATGCAGTAGAGAGCTCGACGTTAAGGCTCATTCACGCTC |
| ZB’A373’ | TATGCAGTAGAGAGCTCGACTATACACTACGGTCGGGAGC |
| ZB’A374’ | TATGCAGTAGAGAGCTCGACCTTATACGCGAGGGTGACAC |
| ZB’A375’ | TATGCAGTAGAGAGCTCGACCCAGAGGCGGTTCGTTATTT |
| ZB’A376’ | TATGCAGTAGAGAGCTCGACGGCGATCCGTTTAACTCGTT |
| ZB’A377’ | TATGCAGTAGAGAGCTCGACCGCGTTCGTTGTGCAAAGTT |
| ZB’A378’ | TATGCAGTAGAGAGCTCGACCGCTGTACGTTTGCGGACTT |
| ZB’A379’ | TATGCAGTAGAGAGCTCGACCCGAGGATATTTCTTGCGGT |
| ZB’A380’ | TATGCAGTAGAGAGCTCGACGGCTCGCTGCTTAATTGATG |
| ZB’A381’ | TATGCAGTAGAGAGCTCGACTTAGACGAACTTACGCGGGC |
| ZB’A382’ | TATGCAGTAGAGAGCTCGACATACCAATAGGTGGGCACGC |
| ZB’A383’ | TATGCAGTAGAGAGCTCGACGTGCGCCGTCAGCTCAATTA |
| ZB’A384’ | TATGCAGTAGAGAGCTCGACCGTCGAGCGCCTAAGAAGAA |
| ZC’B1’ | TGACACCTTGTCCCGGGTTCCGGGCCTATGTACGCTAATC |
| ZC’B2’ | TGACACCTTGTCCCGGGTTCCTCGACTTAGCCGATCTTGG |
| ZC’B3’ | TGACACCTTGTCCCGGGTTCGGTGGCGATTCGTCCAATAT |
| ZC’B4’ | TGACACCTTGTCCCGGGTTCGGTCCGCTTGACTAGCATTT |
| ZC’B5’ | TGACACCTTGTCCCGGGTTCTGAATGTGTTGTAGTCCGCC |
| ZC’B6’ | TGACACCTTGTCCCGGGTTCGCGCTGAATGTCCTGTATCT |
| ZC’B7’ | TGACACCTTGTCCCGGGTTCCGACAGCGTGTTCTTTGATG |
| ZC’B8’ | TGACACCTTGTCCCGGGTTCCGACATGCTACCTGAGTCTG |
| ZC’B9’ | TGACACCTTGTCCCGGGTTCGCTCAGGTTCGACGTGATTA |
| ZC’B10’ | TGACACCTTGTCCCGGGTTCCGATATGTGCCGTAGTCGTT |
| ZC’B11’ | TGACACCTTGTCCCGGGTTCCTATCGAATGGGTCATGCGT |
| ZC’B12’ | TGACACCTTGTCCCGGGTTCGGCAATCGTGTTCAGGATCA |
| ZC’B13’ | TGACACCTTGTCCCGGGTTCCAGATACGTCGTGGAAGTCC |
| ZC’B14’ | TGACACCTTGTCCCGGGTTCCAGCACGCTCTATCGAGAAT |
| ZC’B15’ | TGACACCTTGTCCCGGGTTCGGCGCATGTGTGTAAATCTC |
| ZC’B16’ | TGACACCTTGTCCCGGGTTCTGGCGGTCTTTCAGATCATG |
| ZC’B17’ | TGACACCTTGTCCCGGGTTCGATGGCGATTCAGTCGAACT |
| ZC’B18’ | TGACACCTTGTCCCGGGTTCCATATCAGTCTCGACCGTGG |
| ZC’B19’ | TGACACCTTGTCCCGGGTTCCATCAGTGTCGGTGAGACTC |
| ZC’B20’ | TGACACCTTGTCCCGGGTTCCATTCAACCAGCTCGATGGA |
| ZC’B21’ | TGACACCTTGTCCCGGGTTCCATTCGGCAATCTGGACTGA |
| ZC’B22’ | TGACACCTTGTCCCGGGTTCTCACGATGTGTTCGGATCTG |
| ZC’B23’ | TGACACCTTGTCCCGGGTTCCATTGCCAAATATCGCGGTC |
| ZC’B24’ | TGACACCTTGTCCCGGGTTCCACATCTATCTAGCGCGAGG |
| ZC’B25’ | TGACACCTTGTCCCGGGTTCGACGCTTATGCGAGTGTACT |
| ZC’B26’ | TGACACCTTGTCCCGGGTTCTAAGGCTTGCGCGAGATTTA |
| ZC’B27’ | TGACACCTTGTCCCGGGTTCCCAGCTAGTCGTGACGTATG |
| ZC’B28’ | TGACACCTTGTCCCGGGTTCCCCACGGCTTAAACGGTATT |
| ZC’B29’ | TGACACCTTGTCCCGGGTTCCCCGAACGTCTATTGTTTGC |
| ZC’B30’ | TGACACCTTGTCCCGGGTTCCCGCTTAACCCGATGTGTAT |
| ZC’B31’ | TGACACCTTGTCCCGGGTTCCCGTCGTTAAGCCCGATTAT |
| ZC’B32’ | TGACACCTTGTCCCGGGTTCTATCAGTTGGCTCAGTGTGC |
| ZC’B33’ | TGACACCTTGTCCCGGGTTCCGACACAGTCTTCTTTGGCT |
| ZC’B34’ | TGACACCTTGTCCCGGGTTCCGACACGCTGTAGTTGTTTC |
| ZC’B35’ | TGACACCTTGTCCCGGGTTCCGACACTTAACGTACTGCGA |
| ZC’B36’ | TGACACCTTGTCCCGGGTTCCGACGCCCTCTGATAGTAGA |
| ZC’B37’ | TGACACCTTGTCCCGGGTTCCGAGAGATTAACGCCTGTGT |
| ZC’B38’ | TGACACCTTGTCCCGGGTTCCGAGCTGTCATGCACTCTTA |
| ZC’B39’ | TGACACCTTGTCCCGGGTTCCGCTAGTGTCGCTACCTATG |
| ZC’B40’ | TGACACCTTGTCCCGGGTTCCGCTGTCATCGACGTTACTA |
| ZC’B41’ | TGACACCTTGTCCCGGGTTCCGGCATATTCCCAAAGCTGA |
| ZC’B42’ | TGACACCTTGTCCCGGGTTCGACGGAATTGAACCAAGTGC |
| ZC’B43’ | TGACACCTTGTCCCGGGTTCGACGGGCATGTTCAATAAGC |
| ZC’B44’ | TGACACCTTGTCCCGGGTTCGCGCTCGTTATCTCCATGTA |
| ZC’B45’ | TGACACCTTGTCCCGGGTTCTGTACGTCTTTCATCAGGCG |
| ZC’B46’ | TGACACCTTGTCCCGGGTTCATTCCGCTGCCTACAGATTG |
| ZC’B47’ | TGACACCTTGTCCCGGGTTCCTCACGAATCGGTTGTTTGG |
| ZC’B48’ | TGACACCTTGTCCCGGGTTCCTGCTGAATGGTCGAGACTC |
| ZC’B49’ | TGACACCTTGTCCCGGGTTCCTTACTACTGCTTAGCGCGT |
| ZC’B50’ | TGACACCTTGTCCCGGGTTCCTTTGTTCGTCTAAACGCCG |
| ZC’B51’ | TGACACCTTGTCCCGGGTTCGAACAACGTCTCGGTTAGCT |
| ZC’B52’ | TGACACCTTGTCCCGGGTTCCGTCTCGATGTCTAACCTGG |
| ZC’B53’ | TGACACCTTGTCCCGGGTTCCGTTAAGTCCGCTGGGTAAA |
| ZC’B54’ | TGACACCTTGTCCCGGGTTCGATTCGACAAGCTGCGATTC |
| ZC’B55’ | TGACACCTTGTCCCGGGTTCGCGAAGCATGTTAGCAAGTC |
| ZC’B56’ | TGACACCTTGTCCCGGGTTCGCGACTCATGGTGTGAATCT |
| ZC’B57’ | TGACACCTTGTCCCGGGTTCGCGCCACCTGTTATTTCATG |
| ZC’B58’ | TGACACCTTGTCCCGGGTTCGCGCGAGTTCTTTATCCTCA |
| ZC’B59’ | TGACACCTTGTCCCGGGTTCGCGGGCGTTATGCTTATACT |
| ZC’B60’ | TGACACCTTGTCCCGGGTTCGCGTAAGCTCGTGTGATACT |
| ZC’B61’ | TGACACCTTGTCCCGGGTTCGGCACATCTGTTTGTTGAGC |
| ZC’B62’ | TGACACCTTGTCCCGGGTTCGTGATCGAGTCTGTCTGCTC |
| ZC’B63’ | TGACACCTTGTCCCGGGTTCGTTGCCGTAACTTCTTCAGC |
| ZC’B64’ | TGACACCTTGTCCCGGGTTCTACTTGCTTGAATGTGCGGA |
| ZC’B65’ | TGACACCTTGTCCCGGGTTCTATTGGGTCACTGTTGCGAC |
| ZC’B66’ | TGACACCTTGTCCCGGGTTCTGGTAATTTATGTGGCCGCA |
| ZC’B67’ | TGACACCTTGTCCCGGGTTCTGTATGTTGTGGCGAATCCC |
| ZC’B68’ | TGACACCTTGTCCCGGGTTCTGTTAGTTTCATGGCCGGAC |
| ZC’B69’ | TGACACCTTGTCCCGGGTTCAAGCGAATGAAGGACCGTTT |
| ZC’B70’ | TGACACCTTGTCCCGGGTTCGGCGATGTTCGTAACCCTTA |
| ZC’B71’ | TGACACCTTGTCCCGGGTTCACTTAGCAAACGTCAGGGTC |
| ZC’B72’ | TGACACCTTGTCCCGGGTTCATAGGGTATCGTCCGACTCG |
| ZC’B73’ | TGACACCTTGTCCCGGGTTCGTTTCGCAATCTGTTACGGG |
| ZC’B74’ | TGACACCTTGTCCCGGGTTCTCCAGGTCTTTAACGACGTG |
| ZC’B75’ | TGACACCTTGTCCCGGGTTCGAACCACATTATCGGTCGGT |
| ZC’B76’ | TGACACCTTGTCCCGGGTTCGCAACGACTTTGTTTGACGT |
| ZC’B77’ | TGACACCTTGTCCCGGGTTCGGACGCAGTTGAAAGTACCT |
| ZC’B78’ | TGACACCTTGTCCCGGGTTCAGTGGAACGTAATGAGCACC |
| ZC’B79’ | TGACACCTTGTCCCGGGTTCATTACCGATACTGCGCGTAC |
| ZC’B80’ | TGACACCTTGTCCCGGGTTCTTGTTCAGTTTGCGGAAAGC |
| ZC’B81’ | TGACACCTTGTCCCGGGTTCAACGAGTATCGCACGTACAG |
| ZC’B82’ | TGACACCTTGTCCCGGGTTCAATGGAACGATAGTCAGCCG |
| ZC’B83’ | TGACACCTTGTCCCGGGTTCCACAGTACGCTATACGTGGG |
| ZC’B84’ | TGACACCTTGTCCCGGGTTCCGATACCGTGTCCGTAGATG |
| ZC’B85’ | TGACACCTTGTCCCGGGTTCCTACTGTACCGTGCTCGAAG |
| ZC’B86’ | TGACACCTTGTCCCGGGTTCACGACCCATCTACGGAATGA |
| ZC’B87’ | TGACACCTTGTCCCGGGTTCAGCGAATCTACGGTACTCGA |
| ZC’B88’ | TGACACCTTGTCCCGGGTTCAGGACGAGTCGCGTATCTAA |
| ZC’B89’ | TGACACCTTGTCCCGGGTTCTCTACTTTGCTCGTACCGGA |
| ZC’B90’ | TGACACCTTGTCCCGGGTTCGCTTTCGTATTATCGCCACG |
| ZC’B91’ | TGACACCTTGTCCCGGGTTCTGCTTCGCTTTATCCAAGGG |
| ZC’B92’ | TGACACCTTGTCCCGGGTTCTTAGATAGTTTCGCTGCCGG |
| ZC’B93’ | TGACACCTTGTCCCGGGTTCTTCGACAGTTTGCTAGTCCG |
| ZC’B94’ | TGACACCTTGTCCCGGGTTCAGAATCAATGCAACGGTCGT |
| ZC’B95’ | TGACACCTTGTCCCGGGTTCAGCTTAGCGACAATGCCTTT |
| ZC’B96’ | TGACACCTTGTCCCGGGTTCCACACGGATGCTAACCAGTT |
| ZC’B97’ | TGACACCTTGTCCCGGGTTCCTCAGTCGTTAGCGTCGATT |
| ZC’B98’ | TGACACCTTGTCCCGGGTTCCTCTCACGTTACGGCTGATT |
| ZC’B99’ | TGACACCTTGTCCCGGGTTCCTGACCCATGCGGTTACATT |
| ZC’B100’ | TGACACCTTGTCCCGGGTTCCTGCAAAGTTACGTCGCATT |
| ZC’B101’ | TGACACCTTGTCCCGGGTTCCTTCCCAGCGCGTATGTAAT |
| ZC’B102’ | TGACACCTTGTCCCGGGTTCGAGATTCCGACCGCTTCAAT |
| ZC’B103’ | TGACACCTTGTCCCGGGTTCGGGCAATGTTCGACGTATCT |
| ZC’B104’ | TGACACCTTGTCCCGGGTTCTACGCCTCTTGGAGACTGTT |
| ZC’B105’ | TGACACCTTGTCCCGGGTTCCCAACTGATGCGCCTAAGTA |
| ZC’B106’ | TGACACCTTGTCCCGGGTTCCCGCGCAGTGTACTATGTAA |
| ZC’B107’ | TGACACCTTGTCCCGGGTTCCCGCTATTCAAAGGCTGTGA |
| ZC’B108’ | TGACACCTTGTCCCGGGTTCGGCACCCATGTTCGACAATA |
| ZC’B109’ | TGACACCTTGTCCCGGGTTCGGCAGACTTCGCTGGAATTA |
| ZC’B110’ | TGACACCTTGTCCCGGGTTCGTTCTCGCTGTCTGTCCAAA |
| ZC’B111’ | TGACACCTTGTCCCGGGTTCGAAGTCTCTGCGCTTAGGTC |
| ZC’B112’ | TGACACCTTGTCCCGGGTTCTAATGAAATTGAAGCGGCGC |
| ZC’B113’ | TGACACCTTGTCCCGGGTTCATGCCGCGTCTCTACAATAG |
| ZC’B114’ | TGACACCTTGTCCCGGGTTCCGTGCGAATGTAGCATGATC |
| ZC’B115’ | TGACACCTTGTCCCGGGTTCGATGCCGTTATCGTGAATGC |
| ZC’B116’ | TGACACCTTGTCCCGGGTTCGTTGATGTCATGTGTCGCAC |
| ZC’B117’ | TGACACCTTGTCCCGGGTTCACAAATGCAACAGCGTCTTG |
| ZC’B118’ | TGACACCTTGTCCCGGGTTCTAAGACGATGTTGCCCACTG |
| ZC’B119’ | TGACACCTTGTCCCGGGTTCTGAACGTGTCGTGTTATGCA |
| ZC’B120’ | TGACACCTTGTCCCGGGTTCGACAAAGCTGTAAGTGCTGC |
| ZC’B121’ | TGACACCTTGTCCCGGGTTCTAAGCACATTGATGCCGTGT |
| ZC’B122’ | TGACACCTTGTCCCGGGTTCCCGGCACATGCTAATACTGA |
| ZC’B123’ | TGACACCTTGTCCCGGGTTCGAAGTGCATGGCGTCTCATA |
| ZC’B124’ | TGACACCTTGTCCCGGGTTCCCGCACGATGCTCATAGTAT |
| ZC’B125’ | TGACACCTTGTCCCGGGTTCCTACATAATGCGTGCCTCGT |
| ZC’B126’ | TGACACCTTGTCCCGGGTTCGAGCTGTCTCGGATGCTATC |
| ZC’B127’ | TGACACCTTGTCCCGGGTTCTGAAATGTTGTCGGGCTCTC |
| ZC’B128’ | TGACACCTTGTCCCGGGTTCGCGGACTCTGTGACTATGTC |
| ZC’B129’ | TGACACCTTGTCCCGGGTTCTGACCACTTGTGCCAAATGT |
| ZC’B130’ | TGACACCTTGTCCCGGGTTCGCAATGATTAGCTCTGTGCG |
| ZC’B131’ | TGACACCTTGTCCCGGGTTCTACAGACATTGCGAGGATGC |
| ZC’B132’ | TGACACCTTGTCCCGGGTTCTGACGTTGTGGACGATTCTC |
| ZC’B133’ | TGACACCTTGTCCCGGGTTCTGTCTATGTTTACAGCGGGC |
| ZC’B134’ | TGACACCTTGTCCCGGGTTCTGACTGAGTTTAGCTGCTGC |
| ZC’B135’ | TGACACCTTGTCCCGGGTTCCAACAGAATGCACGTCAGTC |
| ZC’B136’ | TGACACCTTGTCCCGGGTTCGCACGCCTTATGCCTATGTA |
| ZC’B137’ | TGACACCTTGTCCCGGGTTCTACCCAGATTGCGTAAGCTG |
| ZC’B138’ | TGACACCTTGTCCCGGGTTCCAACCATCTCGCAGGTGAAT |
| ZC’B139’ | TGACACCTTGTCCCGGGTTCTACGCATTGCTCACCATTGA |
| ZC’B140’ | TGACACCTTGTCCCGGGTTCGCAGACACTTCCAGTCGATT |
| ZC’B141’ | TGACACCTTGTCCCGGGTTCGCTACTGGTCTCTGATGTCG |
| ZC’B142’ | TGACACCTTGTCCCGGGTTCGCAGATTGAGAGCGTCTTCT |
| ZC’B143’ | TGACACCTTGTCCCGGGTTCTGATCGCGTTTCATACCTGG |
| ZC’B144’ | TGACACCTTGTCCCGGGTTCTGCAACTCTGTTCTTCGCAA |
| ZC’B145’ | TGACACCTTGTCCCGGGTTCATCAAGCGTGGATGACGATC |
| ZC’B146’ | TGACACCTTGTCCCGGGTTCGCAGTCAGTTCATACCGTGT |
| ZC’B147’ | TGACACCTTGTCCCGGGTTCGCAGTCGCTTCAGCTAGATT |
| ZC’B148’ | TGACACCTTGTCCCGGGTTCGCTCTATCTGTGTCACTCGG |
| ZC’B149’ | TGACACCTTGTCCCGGGTTCTCCAGCTCTCTTGCAGTGTA |
| ZC’B150’ | TGACACCTTGTCCCGGGTTCTTCAAGCGTTTGCACATCTG |
| ZC’B151’ | TGACACCTTGTCCCGGGTTCCACACACATGCCAGATGAGT |
| ZC’B152’ | TGACACCTTGTCCCGGGTTCTTCCATGCTTTCTGAGACGG |
| ZC’B153’ | TGACACCTTGTCCCGGGTTCCATCTGGAGTACGCTCTGTG |
| ZC’B154’ | TGACACCTTGTCCCGGGTTCCGCATACGTGTGGACTGATA |
| ZC’B155’ | TGACACCTTGTCCCGGGTTCTAGGAGCTTGTACCGCATTG |
| ZC’B156’ | TGACACCTTGTCCCGGGTTCCATGCGCCTCTCGAAGATAT |
| ZC’B157’ | TGACACCTTGTCCCGGGTTCGCTGGTTCAGTGTCTGCATA |
| ZC’B158’ | TGACACCTTGTCCCGGGTTCGCTGTGATTAGTGTCTGCCA |
| ZC’B159’ | TGACACCTTGTCCCGGGTTCTCGACTAGGTTCGTTGCATG |
| ZC’B160’ | TGACACCTTGTCCCGGGTTCGAGTGCAATTAAGCCTGTGC |
| ZC’B161’ | TGACACCTTGTCCCGGGTTCGCTTCGAGTGCATTCAGTTC |
| ZC’B162’ | TGACACCTTGTCCCGGGTTCTCGCAAGATTTACGCAGTGT |
| ZC’B163’ | TGACACCTTGTCCCGGGTTCAACACCGATCCATAGCGATG |
| ZC’B164’ | TGACACCTTGTCCCGGGTTCAACAGGAGTCGAAGCATCTG |
| ZC’B165’ | TGACACCTTGTCCCGGGTTCCGCATTAAGGCAGCTCTAGT |
| ZC’B166’ | TGACACCTTGTCCCGGGTTCGCATCCTGTGCTCGCATATA |
| ZC’B167’ | TGACACCTTGTCCCGGGTTCGCATCGAATGGTGATCGTCT |
| ZC’B168’ | TGACACCTTGTCCCGGGTTCCGCCAGCATTAGGAGTGATT |
| ZC’B169’ | TGACACCTTGTCCCGGGTTCCGCCATAGTGGATGAGCTAC |
| ZC’B170’ | TGACACCTTGTCCCGGGTTCTATAGCGCGTTAGTTGCGAT |
| ZC’B171’ | TGACACCTTGTCCCGGGTTCCGGCGTACTGCTCCTATATG |
| ZC’B172’ | TGACACCTTGTCCCGGGTTCCTGTCGCGTCATGTCCATTA |
| ZC’B173’ | TGACACCTTGTCCCGGGTTCTCATGGTGTTGAGTCTGCAC |
| ZC’B174’ | TGACACCTTGTCCCGGGTTCGTCACAGCTCTTGCCAAATG |
| ZC’B175’ | TGACACCTTGTCCCGGGTTCTACTTTCATCATGTCGGGCG |
| ZC’B176’ | TGACACCTTGTCCCGGGTTCTTACAAGCTGTTCATTGCGC |
| ZC’B177’ | TGACACCTTGTCCCGGGTTCAACTAGCGTATCGCAGACTG |
| ZC’B178’ | TGACACCTTGTCCCGGGTTCAGCAGCACTCGTACCAGATA |
| ZC’B179’ | TGACACCTTGTCCCGGGTTCCATTCGTGGACAGTCATGGT |
| ZC’B180’ | TGACACCTTGTCCCGGGTTCCGCTTACAGCCTCTGATGAA |
| ZC’B181’ | TGACACCTTGTCCCGGGTTCCACCCGGTCGTATCTGAAAG |
| ZC’B182’ | TGACACCTTGTCCCGGGTTCCACCGATTAAACGAGCTTCG |
| ZC’B183’ | TGACACCTTGTCCCGGGTTCGCCTACAGTTCGAGAGTGTC |
| ZC’B184’ | TGACACCTTGTCCCGGGTTCCACTATCGTCCGTGAGTCAG |
| ZC’B185’ | TGACACCTTGTCCCGGGTTCCACTCGTATGACTGCACTGT |
| ZC’B186’ | TGACACCTTGTCCCGGGTTCCACTCGTCTAAGTCGGTCTG |
| ZC’B187’ | TGACACCTTGTCCCGGGTTCCGTCATCTGTGAAGAGTGCT |
| ZC’B188’ | TGACACCTTGTCCCGGGTTCCTGCGATTAGATCCGCTTGT |
| ZC’B189’ | TGACACCTTGTCCCGGGTTCAACTGCTGCACCACGATTAA |
| ZC’B190’ | TGACACCTTGTCCCGGGTTCTCAGCGCCTTTAACCAATGT |
| ZC’B191’ | TGACACCTTGTCCCGGGTTCTCAACGTATTGGGCGTGATT |
| ZC’B192’ | TGACACCTTGTCCCGGGTTCTGTAGCGATTTCGTGTAGCC |
| ZC’B193’ | TGACACCTTGTCCCGGGTTCGCGCTTCATGCCTCTTAGTA |
| ZC’B194’ | TGACACCTTGTCCCGGGTTCTGTATGGCTTTCGAGATCGC |
| ZC’B195’ | TGACACCTTGTCCCGGGTTCATTGCTCAGAAGTCGTGCAT |
| ZC’B196’ | TGACACCTTGTCCCGGGTTCGAATACGCTGTGATCGAGCT |
| ZC’B197’ | TGACACCTTGTCCCGGGTTCCCCTAGTATGCTGAACGTCG |
| ZC’B198’ | TGACACCTTGTCCCGGGTTCCCACTACATGCGACGTGATA |
| ZC’B199’ | TGACACCTTGTCCCGGGTTCCGCGGTTAGAAACTCTGGTT |
| ZC’B200’ | TGACACCTTGTCCCGGGTTCTCCATCTGTTCGTCTGCAAG |
| ZC’B201’ | TGACACCTTGTCCCGGGTTCCACAAGCATCTGTCAAGTGC |
| ZC’B202’ | TGACACCTTGTCCCGGGTTCGGCTAGACTTGTTTCGTCGA |
| ZC’B203’ | TGACACCTTGTCCCGGGTTCGGGCAGCATGTTATACGTCT |
| ZC’B204’ | TGACACCTTGTCCCGGGTTCCGCATCCATCGTGCATAATG |
| ZC’B205’ | TGACACCTTGTCCCGGGTTCCAGCGACGTATCGTAGAGTC |
| ZC’B206’ | TGACACCTTGTCCCGGGTTCCAGTGCAATCGGTCCTAGTC |
| ZC’B207’ | TGACACCTTGTCCCGGGTTCGCCCGTTCGCCAAGATTATA |
| ZC’B208’ | TGACACCTTGTCCCGGGTTCCAGGTCGCTGATACTAGCTG |
| ZC’B209’ | TGACACCTTGTCCCGGGTTCGCCGCACTTATGTTTGTAGC |
| ZC’B210’ | TGACACCTTGTCCCGGGTTCCAGGTGATCTAACGTCGGAC |
| ZC’B211’ | TGACACCTTGTCCCGGGTTCTCTTAGATTGTCGCTACGGC |
| ZC’B212’ | TGACACCTTGTCCCGGGTTCTAACACTGTCTGTGCTTCGG |
| ZC’B213’ | TGACACCTTGTCCCGGGTTCAGGCGATATAGAGCGTCTCA |
| ZC’B214’ | TGACACCTTGTCCCGGGTTCGCCAAGCATGGTTAGCAGTA |
| ZC’B215’ | TGACACCTTGTCCCGGGTTCTATCACTGTTGAGTCGTGGC |
| ZC’B216’ | TGACACCTTGTCCCGGGTTCCTGTGGCGTATGGCTCAATA |
| ZC’B217’ | TGACACCTTGTCCCGGGTTCAATATCGGCATCCGTCCATG |
| ZC’B218’ | TGACACCTTGTCCCGGGTTCAATATGAAGAGCCTGCCGTG |
| ZC’B219’ | TGACACCTTGTCCCGGGTTCGTGGTCACTTCTCTATGGCG |
| ZC’B220’ | TGACACCTTGTCCCGGGTTCTGCCGTCATTTGTCTACAGG |
| ZC’B221’ | TGACACCTTGTCCCGGGTTCTTGGTGGTTGTTGACACACA |
| ZC’B222’ | TGACACCTTGTCCCGGGTTCAAGTACATGACACCGATGGC |
| ZC’B223’ | TGACACCTTGTCCCGGGTTCAGCGCAAGTGGCATTAGAAT |
| ZC’B224’ | TGACACCTTGTCCCGGGTTCTATGCACGTTGGTGACGATT |
| ZC’B225’ | TGACACCTTGTCCCGGGTTCCCGACTACTGCGGGTTATTT |
| ZC’B226’ | TGACACCTTGTCCCGGGTTCCCGATGCGTAGTACCCTAGA |
| ZC’B227’ | TGACACCTTGTCCCGGGTTCCGTCATGCTGAGTATCGTGT |
| ZC’B228’ | TGACACCTTGTCCCGGGTTCGATCGGCATTCCAGTGACTT |
| ZC’B229’ | TGACACCTTGTCCCGGGTTCGGCACGCATGTGTATCTAGT |
| ZC’B230’ | TGACACCTTGTCCCGGGTTCTATCGCTGTTGCTAGATGGC |
| ZC’B231’ | TGACACCTTGTCCCGGGTTCCGCAACATTAGACCGACTGA |
| ZC’B232’ | TGACACCTTGTCCCGGGTTCCGCATAAATGTCACTCGCTG |
| ZC’B233’ | TGACACCTTGTCCCGGGTTCCGCATGACTGGTAATCGCTA |
| ZC’B234’ | TGACACCTTGTCCCGGGTTCCGTATCGTGCTACCTGCTAG |
| ZC’B235’ | TGACACCTTGTCCCGGGTTCTCACCATATTGTCGGTCGTG |
| ZC’B236’ | TGACACCTTGTCCCGGGTTCCTGCGCTGGCTGTAGATAAT |
| ZC’B237’ | TGACACCTTGTCCCGGGTTCCTTCAGTCCCGTTACGATGG |
| ZC’B238’ | TGACACCTTGTCCCGGGTTCCTTCGTGGCTAGTCTGTGAC |
| ZC’B239’ | TGACACCTTGTCCCGGGTTCGAATTTCCGACATCTTCGCG |
| ZC’B240’ | TGACACCTTGTCCCGGGTTCGACAAGCATTCCAGCAGCTA |
| ZC’B241’ | TGACACCTTGTCCCGGGTTCGACACAGCTCTCTATGGTGC |
| ZC’B242’ | TGACACCTTGTCCCGGGTTCCGTTTGGACATAATGCCTGC |
| ZC’B243’ | TGACACCTTGTCCCGGGTTCGCCGTATTCGCAACTCTTTG |
| ZC’B244’ | TGACACCTTGTCCCGGGTTCGGCGACCATGCTTTATTTGG |
| ZC’B245’ | TGACACCTTGTCCCGGGTTCGTATTGCACCGTGTTCCTCA |
| ZC’B246’ | TGACACCTTGTCCCGGGTTCGTCACATGGGATTCGCTTGA |
| ZC’B247’ | TGACACCTTGTCCCGGGTTCGTCGTTCCTGTGTATGGCAA |
| ZC’B248’ | TGACACCTTGTCCCGGGTTCGTTGTCAAACGTGCTTCTGG |
| ZC’B249’ | TGACACCTTGTCCCGGGTTCGTTTCGCCGGTGTTGAATAC |
| ZC’B250’ | TGACACCTTGTCCCGGGTTCTAAGCACTTGCCCATCGTTT |
| ZC’B251’ | TGACACCTTGTCCCGGGTTCTAATTGCGACGGTGTTCGTA |
| ZC’B252’ | TGACACCTTGTCCCGGGTTCTACGGCTATGTGTTATCGGC |
| ZC’B253’ | TGACACCTTGTCCCGGGTTCTCACGGTATGTTCGCGTATC |
| ZC’B254’ | TGACACCTTGTCCCGGGTTCTCCAAACTTGCTGTTCTGCA |
| ZC’B255’ | TGACACCTTGTCCCGGGTTCTCCAAATATCTTGCGCTCGT |
| ZC’B256’ | TGACACCTTGTCCCGGGTTCTCCCATTGTATCGTATGCGC |
| ZC’B257’ | TGACACCTTGTCCCGGGTTCCTACAAGTTACGACCGGCTT |
| ZC’B258’ | TGACACCTTGTCCCGGGTTCCTGAACGCTTAGGTAACCGT |
| ZC’B259’ | TGACACCTTGTCCCGGGTTCCAAATTGGTACAACCTGCCG |
| ZC’B260’ | TGACACCTTGTCCCGGGTTCCGCAAGCGTTAAACCTATGG |
| ZC’B261’ | TGACACCTTGTCCCGGGTTCCGGCTTCCAACACGTTAATG |
| ZC’B262’ | TGACACCTTGTCCCGGGTTCCTACGCCCTTACGAAGATGG |
| ZC’B263’ | TGACACCTTGTCCCGGGTTCAAAGCTAATCGCAAGTCCGT |
| ZC’B264’ | TGACACCTTGTCCCGGGTTCAACCCGCTGAACGATCATTT |
| ZC’B265’ | TGACACCTTGTCCCGGGTTCAACGAGCTAGAGTACGACGT |
| ZC’B266’ | TGACACCTTGTCCCGGGTTCAACTGTGCGAACTCAGGTTT |
| ZC’B267’ | TGACACCTTGTCCCGGGTTCCGACGGCTTAACCAGCTATT |
| ZC’B268’ | TGACACCTTGTCCCGGGTTCCGCATAGGTTAAGCGGACTT |
| ZC’B269’ | TGACACCTTGTCCCGGGTTCCGCCGCAGTTAAGGAGTATT |
| ZC’B270’ | TGACACCTTGTCCCGGGTTCCGCCGGACTTACAGTACATT |
| ZC’B271’ | TGACACCTTGTCCCGGGTTCCGCGACTCTTACCAAGGATT |
| ZC’B272’ | TGACACCTTGTCCCGGGTTCCGCGAGGATTACACGTACTT |
| ZC’B273’ | TGACACCTTGTCCCGGGTTCGTACGCCCTTCGTCGATATT |
| ZC’B274’ | TGACACCTTGTCCCGGGTTCTAAACCCATTGCGAACGTCT |
| ZC’B275’ | TGACACCTTGTCCCGGGTTCTCGCCACCTTTACGAATGTT |
| ZC’B276’ | TGACACCTTGTCCCGGGTTCGAGTGTCCTGTACGTTAGCC |
| ZC’B277’ | TGACACCTTGTCCCGGGTTCGGAACGGCTGTTATTGCTTC |
| ZC’B278’ | TGACACCTTGTCCCGGGTTCTCGTGTGGTTTAAGATGCCC |
| ZC’B279’ | TGACACCTTGTCCCGGGTTCTGTAACCTTGTGTGTACGGC |
| ZC’B280’ | TGACACCTTGTCCCGGGTTCTTAACTCTTGTGGCTACGGC |
| ZC’B281’ | TGACACCTTGTCCCGGGTTCTTAGGGCGTTTGATGTCACC |
| ZC’B282’ | TGACACCTTGTCCCGGGTTCTTCGGGTGTTTGCATAGACC |
| ZC’B283’ | TGACACCTTGTCCCGGGTTCAGAAGTACGCGATGAATCCG |
| ZC’B284’ | TGACACCTTGTCCCGGGTTCCAAAGTCGTCTCCGCTAGAG |
| ZC’B285’ | TGACACCTTGTCCCGGGTTCCACGCACCTGTAAATCGGTA |
| ZC’B286’ | TGACACCTTGTCCCGGGTTCTTTAAGCTGTTTAGGGCGCA |
| ZC’B287’ | TGACACCTTGTCCCGGGTTCAACATGCTGAACCGGAGTAC |
| ZC’B288’ | TGACACCTTGTCCCGGGTTCAATGCTATCCGCTAACGGTC |
| ZC’B289’ | TGACACCTTGTCCCGGGTTCAATTCAGTCAAGGTAGCGGC |
| ZC’B290’ | TGACACCTTGTCCCGGGTTCAGCAGTAGTGCCTAAACGTC |
| ZC’B291’ | TGACACCTTGTCCCGGGTTCTCGCGGGATTTACCATTCTG |
| ZC’B292’ | TGACACCTTGTCCCGGGTTCTCGTCTTCTTGCTCCGAAAG |
| ZC’B293’ | TGACACCTTGTCCCGGGTTCAATCAACTGAACTACGCGCT |
| ZC’B294’ | TGACACCTTGTCCCGGGTTCACGGCTGAGTACAACTAGCT |
| ZC’B295’ | TGACACCTTGTCCCGGGTTCATAACCGCTGCCATCGAATT |
| ZC’B296’ | TGACACCTTGTCCCGGGTTCATAGCGACTCGACTCCGTAT |
| ZC’B297’ | TGACACCTTGTCCCGGGTTCATATAGGATCGCGTCCGTCT |
| ZC’B298’ | TGACACCTTGTCCCGGGTTCATCACTACTGCCGCAAAGTT |
| ZC’B299’ | TGACACCTTGTCCCGGGTTCATGAAGACTCGTCCCTCGAT |
| ZC’B300’ | TGACACCTTGTCCCGGGTTCATTTAGCGAAGGTGCATCGT |
| ZC’B301’ | TGACACCTTGTCCCGGGTTCCGACGTGATGCGGTATAACT |
| ZC’B302’ | TGACACCTTGTCCCGGGTTCCGCAGACGTTAGAAGAGCTT |
| ZC’B303’ | TGACACCTTGTCCCGGGTTCCGCGGCCATTAGGATACATT |
| ZC’B304’ | TGACACCTTGTCCCGGGTTCCGTGCCGGTTAATGAGCTAT |
| ZC’B305’ | TGACACCTTGTCCCGGGTTCGCACGAGCTTCCTAAACTGT |
| ZC’B306’ | TGACACCTTGTCCCGGGTTCGCCTATGCTTCCGATACGTT |
| ZC’B307’ | TGACACCTTGTCCCGGGTTCGCTCTGCGTTCCCTAAATGT |
| ZC’B308’ | TGACACCTTGTCCCGGGTTCACAGCGTATCGGATCAGGTA |
| ZC’B309’ | TGACACCTTGTCCCGGGTTCGCGACGATTCGAGGTCATTA |
| ZC’B310’ | TGACACCTTGTCCCGGGTTCTCCAGGTGTCTTGAGTTCGA |
| ZC’B311’ | TGACACCTTGTCCCGGGTTCTATGCGACTTGCTGACTCTG |
| ZC’B312’ | TGACACCTTGTCCCGGGTTCAGCTCTCTCGCCAGTGATAA |
| ZC’B313’ | TGACACCTTGTCCCGGGTTCGTTCATCATTCTGCTGCACG |
| ZC’B314’ | TGACACCTTGTCCCGGGTTCAATCGCCCTGACACATGAAG |
| ZC’B315’ | TGACACCTTGTCCCGGGTTCCAGATCACTGCGTCAACTGA |
| ZC’B316’ | TGACACCTTGTCCCGGGTTCGCGACATGGTCATCTATGCT |
| ZC’B317’ | TGACACCTTGTCCCGGGTTCGAGCAGCGTTATGAGCATCT |
| ZC’B318’ | TGACACCTTGTCCCGGGTTCATGCGCCATGCTAAAGACTT |
| ZC’B319’ | TGACACCTTGTCCCGGGTTCCAGCAAGTGCGTAGAGATCA |
| ZC’B320’ | TGACACCTTGTCCCGGGTTCCGTGTGAATGAATGGCCTCT |
| ZC’B321’ | TGACACCTTGTCCCGGGTTCGAGCATAATTCACGCGATGG |
| ZC’B322’ | TGACACCTTGTCCCGGGTTCGTTTGCTATTCATGGCGAGC |
| ZC’B323’ | TGACACCTTGTCCCGGGTTCCAGGAAGATGCTACGATCCG |
| ZC’B324’ | TGACACCTTGTCCCGGGTTCTGAACATCTCTTGCTTGCGA |
| ZC’B325’ | TGACACCTTGTCCCGGGTTCCCGCTACATGCAGCTTAGAA |
| ZC’B326’ | TGACACCTTGTCCCGGGTTCCCTGAGAGTGTACGCTGATC |
| ZC’B327’ | TGACACCTTGTCCCGGGTTCACGACATATCAATAGCGCGT |
| ZC’B328’ | TGACACCTTGTCCCGGGTTCATAATCGTGAGCAGTCGTCG |
| ZC’B329’ | TGACACCTTGTCCCGGGTTCCAGTCTCGTGGATAGCACTC |
| ZC’B330’ | TGACACCTTGTCCCGGGTTCTGTGAGCTTGTTACTACGGC |
| ZC’B331’ | TGACACCTTGTCCCGGGTTCCGAACATCTGTCACAATGCG |
| ZC’B332’ | TGACACCTTGTCCCGGGTTCCGAACTGATGCTGAATCGTG |
| ZC’B333’ | TGACACCTTGTCCCGGGTTCCATACAGATCGCTCGTGAGG |
| ZC’B334’ | TGACACCTTGTCCCGGGTTCCTCCCAATGCGTGCGTATAT |
| ZC’B335’ | TGACACCTTGTCCCGGGTTCCATAGCAGTGTAGCCGACTC |
| ZC’B336’ | TGACACCTTGTCCCGGGTTCCGAGCAAGTGTGACAGCTAT |
| ZC’B337’ | TGACACCTTGTCCCGGGTTCTCATCTTTGTAGCTCAGGCG |
| ZC’B338’ | TGACACCTTGTCCCGGGTTCTCATGCAGTGTCTTGACTCG |
| ZC’B339’ | TGACACCTTGTCCCGGGTTCTGATCTATTGTCTACGCGGC |
| ZC’B340’ | TGACACCTTGTCCCGGGTTCTACTGCTGTTGAGTGATGCC |
| ZC’B341’ | TGACACCTTGTCCCGGGTTCCATCCAGCTCGACGTATGAG |
| ZC’B342’ | TGACACCTTGTCCCGGGTTCCGATGCTATCTACTCGGTGC |
| ZC’B343’ | TGACACCTTGTCCCGGGTTCGTCAGTTGCATCAGATTGCG |
| ZC’B344’ | TGACACCTTGTCCCGGGTTCCAATGCTCAAGCCTAGTGCT |
| ZC’B345’ | TGACACCTTGTCCCGGGTTCGCAGTGGATTCAAGTGCTCT |
| ZC’B346’ | TGACACCTTGTCCCGGGTTCGCTCTTCGGAGATGTGTCAA |
| ZC’B347’ | TGACACCTTGTCCCGGGTTCGTCCTCGCTTCGTCTATGAG |
| ZC’B348’ | TGACACCTTGTCCCGGGTTCTAGCACTCTGTGTCTCTGCA |
| ZC’B349’ | TGACACCTTGTCCCGGGTTCGCATCAGATCGTATGTGGCA |
| ZC’B350’ | TGACACCTTGTCCCGGGTTCGTCGGCGATTCTCTAGTCTG |
| ZC’B351’ | TGACACCTTGTCCCGGGTTCTGCCAGATTGTGCGGAATTA |
| ZC’B352’ | TGACACCTTGTCCCGGGTTCAGAGCGAGTGACGCATACTA |
| ZC’B353’ | TGACACCTTGTCCCGGGTTCCACAGACATGCAGCAGTGTA |
| ZC’B354’ | TGACACCTTGTCCCGGGTTCCATGAATCTGACTGCGCTCT |
| ZC’B355’ | TGACACCTTGTCCCGGGTTCATCGAGTAGCTCTCACGTCA |
| ZC’B356’ | TGACACCTTGTCCCGGGTTCGCTGTCTATCGTGTCTCCAG |
| ZC’B357’ | TGACACCTTGTCCCGGGTTCATCGTCATGCGCCTACATAG |
| ZC’B358’ | TGACACCTTGTCCCGGGTTCGCTTCAAGTTCATGCGTCTG |
| ZC’B359’ | TGACACCTTGTCCCGGGTTCTAGTCGCGTTGGCTATATGC |
| ZC’B360’ | TGACACCTTGTCCCGGGTTCTGCGTAAATTGGATTGTCGC |
| ZC’B361’ | TGACACCTTGTCCCGGGTTCAGCACCATCTCGACGATAGT |
| ZC’B362’ | TGACACCTTGTCCCGGGTTCGATACCTCTGTAGCGTGTCG |
| ZC’B363’ | TGACACCTTGTCCCGGGTTCGATACGCATGTCTCCATCGT |
| ZC’B364’ | TGACACCTTGTCCCGGGTTCTAGCTGTGTTGACATCTGGC |
| ZC’B365’ | TGACACCTTGTCCCGGGTTCCGCGTGCAGTTTATGAAGTT |
| ZC’B366’ | TGACACCTTGTCCCGGGTTCGGCGCGTCTTGTATAAACTT |
| ZC’B367’ | TGACACCTTGTCCCGGGTTCCGACCTTATTGTAACGCCAT |
| ZC’B368’ | TGACACCTTGTCCCGGGTTCCGAGGATGTATTATTCCGTG |
| ZC’B369’ | TGACACCTTGTCCCGGGTTCTTGACTGTCCTTCCGCTAGG |
| ZC’B370’ | TGACACCTTGTCCCGGGTTCCTATTAGCGTGTATGCCAGG |
| ZC’B371’ | TGACACCTTGTCCCGGGTTCGGCCAATAACTTAGAGCATC |
| ZC’B372’ | TGACACCTTGTCCCGGGTTCTGCTCCGTGCGTAATACATC |
| ZC’B373’ | TGACACCTTGTCCCGGGTTCATACAGATGGGTAAATCGGC |
| ZC’B374’ | TGACACCTTGTCCCGGGTTCCGCTAGGCAGCTAACATCAA |
| ZC’B375’ | TGACACCTTGTCCCGGGTTCCACCGCAGTTGTTCTGGGTT |
| ZC’B376’ | TGACACCTTGTCCCGGGTTCACCCGTATCTGTTGGTCGGT |
| ZC’B377’ | TGACACCTTGTCCCGGGTTCAGACGCTCTCGTTCTTGGCT |
| ZC’B378’ | TGACACCTTGTCCCGGGTTCAGTGATTCGCTTCCGTCTGG |
| ZC’B379’ | TGACACCTTGTCCCGGGTTCATTCGTGGGTCTCGACATCG |
| ZC’B380’ | TGACACCTTGTCCCGGGTTCAATGCGAAGTGTGAGCACCC |
| ZC’B381’ | TGACACCTTGTCCCGGGTTCAATGCAGAGTCGTCGGAACC |
| ZC’B382’ | TGACACCTTGTCCCGGGTTCATGAGATCAGGTGCCGAACC |
| ZC’B383’ | TGACACCTTGTCCCGGGTTCTAACATCACTGGCCGGAGGA |
| ZC’B384’ | TGACACCTTGTCCCGGGTTCAGTGACCTCAAGTCGCCGAA |
| ZD’C1’ | TACATAGCTGCAGGCCACTACCCAGTGCTGACATCTATGA |
| ZD’C2’ | TACATAGCTGCAGGCCACTACCGCTTACCAGACTGAGTTA |
| ZD’C3’ | TACATAGCTGCAGGCCACTACCGGTGGGTAGCAATATACT |
| ZD’C4’ | TACATAGCTGCAGGCCACTACCGGTTACCCACATGATAGT |
| ZD’C5’ | TACATAGCTGCAGGCCACTAGTGGACTCTTCTGCATCATG |
| ZD’C6’ | TACATAGCTGCAGGCCACTATATCATGCTTGCTAGGCAGT |
| ZD’C7’ | TACATAGCTGCAGGCCACTATCGTACTTGGTCTGATGGAC |
| ZD’C8’ | TACATAGCTGCAGGCCACTAAAGTCTAGGAGTCGCATCAG |
| ZD’C9’ | TACATAGCTGCAGGCCACTACACTGCACTGGACTACTGAT |
| ZD’C10’ | TACATAGCTGCAGGCCACTACGATCACATGGAATGGGAAC |
| ZD’C11’ | TACATAGCTGCAGGCCACTACGCTTCAGGAACTGTAGGTA |
| ZD’C12’ | TACATAGCTGCAGGCCACTACGGACGGTTAATTTCTTCCC |
| ZD’C13’ | TACATAGCTGCAGGCCACTACGGCAGTCTGGTAGAAATCT |
| ZD’C14’ | TACATAGCTGCAGGCCACTACGGCTATTCCCAGAGCTTAT |
| ZD’C15’ | TACATAGCTGCAGGCCACTACGGGATACTGTACCCTGTAG |
| ZD’C16’ | TACATAGCTGCAGGCCACTAGAGCTTCGGAGAGTCCTATC |
| ZD’C17’ | TACATAGCTGCAGGCCACTAACATGCAGTCAAGTCCTCAT |
| ZD’C18’ | TACATAGCTGCAGGCCACTAATTATCTCACCTGGCAGCAT |
| ZD’C19’ | TACATAGCTGCAGGCCACTACAGGATCATCGTGACTGAGT |
| ZD’C20’ | TACATAGCTGCAGGCCACTACTATGGAATGCCTCGTCTCT |
| ZD’C21’ | TACATAGCTGCAGGCCACTACTCAATGGGATTCAACGGAG |
| ZD’C22’ | TACATAGCTGCAGGCCACTACTCACGTCTCCTGATAGGTG |
| ZD’C23’ | TACATAGCTGCAGGCCACTACTCAGGCCGTATCTGCTATT |
| ZD’C24’ | TACATAGCTGCAGGCCACTACTGGTATCTGGCTGGTACAT |
| ZD’C25’ | TACATAGCTGCAGGCCACTAGAAAGAAGTGTGAGCCATCC |
| ZD’C26’ | TACATAGCTGCAGGCCACTAGAAGAGGTTAGCCGTCCTTA |
| ZD’C27’ | TACATAGCTGCAGGCCACTACGTGTAGATGGGCTAATCCT |
| ZD’C28’ | TACATAGCTGCAGGCCACTACGTTATATGAATGGCTGGCC |
| ZD’C29’ | TACATAGCTGCAGGCCACTACGTTGTGGGAAGTCCTATCT |
| ZD’C30’ | TACATAGCTGCAGGCCACTAGATTCAGTGCATGGGTCTTC |
| ZD’C31’ | TACATAGCTGCAGGCCACTAGCAACACCTTTATTGTGGGT |
| ZD’C32’ | TACATAGCTGCAGGCCACTAGCAATGCTTGAAACAATGGG |
| ZD’C33’ | TACATAGCTGCAGGCCACTAGCCTGGTATTCAGCTATGGT |
| ZD’C34’ | TACATAGCTGCAGGCCACTAGCCTTAGTGCTGATGTATGC |
| ZD’C35’ | TACATAGCTGCAGGCCACTAGCGACTCCTGTTACCTATGT |
| ZD’C36’ | TACATAGCTGCAGGCCACTAGGACCCTCTGGTGGTATCTA |
| ZD’C37’ | TACATAGCTGCAGGCCACTAGGAGCTGATGTTAGACCTCA |
| ZD’C38’ | TACATAGCTGCAGGCCACTAGGATATACTTCCGTCGCCTT |
| ZD’C39’ | TACATAGCTGCAGGCCACTAGGCAATTAGGGTCTCTGCTA |
| ZD’C40’ | TACATAGCTGCAGGCCACTAGGTCAATTCAATGGTGTGCT |
| ZD’C41’ | TACATAGCTGCAGGCCACTAGGTCACTGTGTGGAACTCTA |
| ZD’C42’ | TACATAGCTGCAGGCCACTAGGTGGTTATCGAGATCCCTT |
| ZD’C43’ | TACATAGCTGCAGGCCACTAGTCTTATTCGATGTGGGCAC |
| ZD’C44’ | TACATAGCTGCAGGCCACTAGTGACTTCTCCTTCCTGAGG |
| ZD’C45’ | TACATAGCTGCAGGCCACTAGTGTTCTACCTGTGTGGACA |
| ZD’C46’ | TACATAGCTGCAGGCCACTAGTTGGTGAAATGTGTCTCCC |
| ZD’C47’ | TACATAGCTGCAGGCCACTATACTTATTCTGTCGGAGGGC |
| ZD’C48’ | TACATAGCTGCAGGCCACTATAGATCGTTAGTTCCTGGCC |
| ZD’C49’ | TACATAGCTGCAGGCCACTATAGATTGGCACATGGTGTCT |
| ZD’C50’ | TACATAGCTGCAGGCCACTATATCTCTTGAGTGTGGGCAC |
| ZD’C51’ | TACATAGCTGCAGGCCACTATCTATCTGTTGCCCTCGAAG |
| ZD’C52’ | TACATAGCTGCAGGCCACTATGACATTTAGGGTGGTTCCC |
| ZD’C53’ | TACATAGCTGCAGGCCACTATGTGTTTGTGGATGACCAAC |
| ZD’C54’ | TACATAGCTGCAGGCCACTATTCAAATTGGTGTTGGACCG |
| ZD’C55’ | TACATAGCTGCAGGCCACTATTTCCACTTTGTACCCAGGG |
| ZD’C56’ | TACATAGCTGCAGGCCACTAAAACAGGGTCCGATACCATT |
| ZD’C57’ | TACATAGCTGCAGGCCACTAAACGAACTGAACCCTGACTT |
| ZD’C58’ | TACATAGCTGCAGGCCACTAAAGCCCGGTGACTAGAAATT |
| ZD’C59’ | TACATAGCTGCAGGCCACTAAATAACCATGAACCCGGTCT |
| ZD’C60’ | TACATAGCTGCAGGCCACTACGGCAACTTAGGAAGGTCTA |
| ZD’C61’ | TACATAGCTGCAGGCCACTATTCTCCCTTGTTCAACGGAA |
| ZD’C62’ | TACATAGCTGCAGGCCACTAAATGGAAGGAATAGGCCCTC |
| ZD’C63’ | TACATAGCTGCAGGCCACTAAGGTATAATAAGGTGCCCGC |
| ZD’C64’ | TACATAGCTGCAGGCCACTAAAATGCAAACATCCTTCCGG |
| ZD’C65’ | TACATAGCTGCAGGCCACTAAACCCACCTAGTCAGAAGTG |
| ZD’C66’ | TACATAGCTGCAGGCCACTAAACTACAGTCCCATCAAGGG |
| ZD’C67’ | TACATAGCTGCAGGCCACTAAAGTCCAATCTCCACGGTAG |
| ZD’C68’ | TACATAGCTGCAGGCCACTAGTTCCTGCTTATTCCAACGG |
| ZD’C69’ | TACATAGCTGCAGGCCACTATGCTAAGGTTTCCTACTCGG |
| ZD’C70’ | TACATAGCTGCAGGCCACTATGTCTGGGTTTCCTAACAGG |
| ZD’C71’ | TACATAGCTGCAGGCCACTAACCTCCCGTCAGAGAGTATT |
| ZD’C72’ | TACATAGCTGCAGGCCACTAACTTCGGAGACATGACCTTT |
| ZD’C73’ | TACATAGCTGCAGGCCACTAAGAATAGGTGACAGGACCCT |
| ZD’C74’ | TACATAGCTGCAGGCCACTAAGGCAACGTCCCATCTAATT |
| ZD’C75’ | TACATAGCTGCAGGCCACTAAGGGAAGTGACGACCTATCT |
| ZD’C76’ | TACATAGCTGCAGGCCACTAAGGTATAGGGTAGCCACCAT |
| ZD’C77’ | TACATAGCTGCAGGCCACTAATACGCATTACCCACAAGGT |
| ZD’C78’ | TACATAGCTGCAGGCCACTACTAGGGCGTTACATCTCCTT |
| ZD’C79’ | TACATAGCTGCAGGCCACTAGAAGCCCTTATCAGTACGGT |
| ZD’C80’ | TACATAGCTGCAGGCCACTAGAGGAGGTTACGTCACTCTT |
| ZD’C81’ | TACATAGCTGCAGGCCACTAGAGTACGGTGGTCCAACTAT |
| ZD’C82’ | TACATAGCTGCAGGCCACTAGCGGAGTGTTACTACCTCTT |
| ZD’C83’ | TACATAGCTGCAGGCCACTAGGATAGACTTGACCGACCTT |
| ZD’C84’ | TACATAGCTGCAGGCCACTAGGCCTACGTGGTTAGAATCT |
| ZD’C85’ | TACATAGCTGCAGGCCACTAATAGGGAGTCGTCACTGAAC |
| ZD’C86’ | TACATAGCTGCAGGCCACTAATGCCCTGTAAGGTGACTTC |
| ZD’C87’ | TACATAGCTGCAGGCCACTACAAGGAGTGGAAGATCCCTC |
| ZD’C88’ | TACATAGCTGCAGGCCACTAGTTGCCATTAAGTGGGTCTC |
| ZD’C89’ | TACATAGCTGCAGGCCACTATAGGTTGCGGTAGATTCTCC |
| ZD’C90’ | TACATAGCTGCAGGCCACTATATAGGTGTTGCGGATTCCC |
| ZD’C91’ | TACATAGCTGCAGGCCACTATATTGGTGTAGTGTCCGACC |
| ZD’C92’ | TACATAGCTGCAGGCCACTATCAAGACATTGGGTAGGTCC |
| ZD’C93’ | TACATAGCTGCAGGCCACTACAAGAAACTGTCAAGGCTGG |
| ZD’C94’ | TACATAGCTGCAGGCCACTACACCCGCCTAGTAGTAGATG |
| ZD’C95’ | TACATAGCTGCAGGCCACTACAGACTAATCTCCGTCAGGG |
| ZD’C96’ | TACATAGCTGCAGGCCACTACCAGCAACTTAACAGAGTGG |
| ZD’C97’ | TACATAGCTGCAGGCCACTAAGGTACGCTCCTAGTCCATA |
| ZD’C98’ | TACATAGCTGCAGGCCACTAGAACCGGATGGGTCTAATCA |
| ZD’C99’ | TACATAGCTGCAGGCCACTAGACCGGCTTATCAGTCCTTA |
| ZD’C100’ | TACATAGCTGCAGGCCACTAGCCCTTACTCGTTATGGTCA |
| ZD’C101’ | TACATAGCTGCAGGCCACTAGTTCACTTGACTTCCTGGGA |
| ZD’C102’ | TACATAGCTGCAGGCCACTATGATCCTTTATATCCCGGCG |
| ZD’C103’ | TACATAGCTGCAGGCCACTATTCTCCTATTTCGCCAGAGG |
| ZD’C104’ | TACATAGCTGCAGGCCACTAAAACATCATCCCAGAGTCGT |
| ZD’C105’ | TACATAGCTGCAGGCCACTAACTCCCATGCACAATAGGTT |
| ZD’C106’ | TACATAGCTGCAGGCCACTAACTCCCGCTCGATAGATAGT |
| ZD’C107’ | TACATAGCTGCAGGCCACTAACTCTCCGTCACATAGGGAT |
| ZD’C108’ | TACATAGCTGCAGGCCACTAAGGCAGACTGACTAGACCTT |
| ZD’C109’ | TACATAGCTGCAGGCCACTAAGGCTCACTGCACTTAAACT |
| ZD’C110’ | TACATAGCTGCAGGCCACTACACACCCATGATACGGTAGT |
| ZD’C111’ | TACATAGCTGCAGGCCACTACAGGTCCATGACTCGTACTT |
| ZD’C112’ | TACATAGCTGCAGGCCACTACAGGTTACCACCGCTATGAT |
| ZD’C113’ | TACATAGCTGCAGGCCACTACCCTACCTGCGATAAGTGAT |
| ZD’C114’ | TACATAGCTGCAGGCCACTACCGGGTCGTGAGTATCATAT |
| ZD’C115’ | TACATAGCTGCAGGCCACTACTAGTTCGGCAGGCTTAGAT |
| ZD’C116’ | TACATAGCTGCAGGCCACTAGACCGCTCTGTATAATGGGT |
| ZD’C117’ | TACATAGCTGCAGGCCACTAGAGGACCCTTCAAGCTATGT |
| ZD’C118’ | TACATAGCTGCAGGCCACTATACAGGCCGTTAATTCTGCT |
| ZD’C119’ | TACATAGCTGCAGGCCACTACCTGTAATGAGATCGGGTCA |
| ZD’C120’ | TACATAGCTGCAGGCCACTAGGACTCCTTCGCCATGATTA |
| ZD’C121’ | TACATAGCTGCAGGCCACTAGGCTGCCTTCCTATTACTGA |
| ZD’C122’ | TACATAGCTGCAGGCCACTAGGTGTATTCCCTGGTATGCA |
| ZD’C123’ | TACATAGCTGCAGGCCACTAATATGTGGGAAGGCATCCTC |
| ZD’C124’ | TACATAGCTGCAGGCCACTAGATAATTGCACACCCTGGTC |
| ZD’C125’ | TACATAGCTGCAGGCCACTAGATGTCGATGGGTATCCCTC |
| ZD’C126’ | TACATAGCTGCAGGCCACTATAAGCAGATGTTTGTTGCCC |
| ZD’C127’ | TACATAGCTGCAGGCCACTATAGCTGGGTTCTTATCGAGC |
| ZD’C128’ | TACATAGCTGCAGGCCACTATAGGCAAGTGTTCTGGTCTC |
| ZD’C129’ | TACATAGCTGCAGGCCACTAAATCAGCTCAGATACTCGGG |
| ZD’C130’ | TACATAGCTGCAGGCCACTATCTGCACATTTAGCATTGGG |
| ZD’C131’ | TACATAGCTGCAGGCCACTAAGGAAGCATGAGATCAGCTC |
| ZD’C132’ | TACATAGCTGCAGGCCACTATCAAGCCATTGTACCACTGT |
| ZD’C133’ | TACATAGCTGCAGGCCACTAACAGGTCATCGAACTCTCAG |
| ZD’C134’ | TACATAGCTGCAGGCCACTATAAGCAGCTCTTGGAACAGT |
| ZD’C135’ | TACATAGCTGCAGGCCACTAATGTCCAGGAATGCGAGATT |
| ZD’C136’ | TACATAGCTGCAGGCCACTAGAGCTGCATGTGGACCTATA |
| ZD’C137’ | TACATAGCTGCAGGCCACTATCTTGGTGTTGATGGCAAAC |
| ZD’C138’ | TACATAGCTGCAGGCCACTAGATGTGCCTGGCTCTTACTA |
| ZD’C139’ | TACATAGCTGCAGGCCACTAATTGGGCACGATGCACTATA |
| ZD’C140’ | TACATAGCTGCAGGCCACTACAGGGCGCTGTAGACATATA |
| ZD’C141’ | TACATAGCTGCAGGCCACTAGGGCTGATTGCACATATCTG |
| ZD’C142’ | TACATAGCTGCAGGCCACTATGTCCAGCTTTCTACTGAGG |
| ZD’C143’ | TACATAGCTGCAGGCCACTACAAAGCCATGTAGACCATCG |
| ZD’C144’ | TACATAGCTGCAGGCCACTAGGTCAGTTGAGCAGCTATTC |
| ZD’C145’ | TACATAGCTGCAGGCCACTAGGTCCACATTATTTGCTGCA |
| ZD’C146’ | TACATAGCTGCAGGCCACTACTCACAGCTTCAGACAATGG |
| ZD’C147’ | TACATAGCTGCAGGCCACTAGGTCCATTGAGATGACTTGC |
| ZD’C148’ | TACATAGCTGCAGGCCACTATGACTTCTTGTCCATCCGAG |
| ZD’C149’ | TACATAGCTGCAGGCCACTAATACTCCCTGCACAGATGAG |
| ZD’C150’ | TACATAGCTGCAGGCCACTACTCAGCTTAGGCCGCTATAT |
| ZD’C151’ | TACATAGCTGCAGGCCACTAGGTGATAATTCCTCGCTGTG |
| ZD’C152’ | TACATAGCTGCAGGCCACTATACCAGCATGTTCTGCATCT |
| ZD’C153’ | TACATAGCTGCAGGCCACTAATACTGCATATCCCAGGTCG |
| ZD’C154’ | TACATAGCTGCAGGCCACTACAGTGTATGAAGTGGCATCC |
| ZD’C155’ | TACATAGCTGCAGGCCACTATCAGTATCTTGCGATCCTGG |
| ZD’C156’ | TACATAGCTGCAGGCCACTAATAGGCCATCTGCCCAATAG |
| ZD’C157’ | TACATAGCTGCAGGCCACTAGCTCCTCCTTCATGGAATGT |
| ZD’C158’ | TACATAGCTGCAGGCCACTAACTGGCACCCTCAATGATAG |
| ZD’C159’ | TACATAGCTGCAGGCCACTACATCATGTGCCTCACTAGGA |
| ZD’C160’ | TACATAGCTGCAGGCCACTACTCTGCACTGTCATCAATGG |
| ZD’C161’ | TACATAGCTGCAGGCCACTATGCAATGATTTGGTGTGACC |
| ZD’C162’ | TACATAGCTGCAGGCCACTACTGAATGTGAATGCAGGCTT |
| ZD’C163’ | TACATAGCTGCAGGCCACTATGCATGAGTTTGTAATGGCC |
| ZD’C164’ | TACATAGCTGCAGGCCACTATCCTGAGTTGTCATAGTGGC |
| ZD’C165’ | TACATAGCTGCAGGCCACTATCCTGATCTTGGTACTCACG |
| ZD’C166’ | TACATAGCTGCAGGCCACTACACATCCCTGAGCATAGTGA |
| ZD’C167’ | TACATAGCTGCAGGCCACTAGCTGTACCTGTATGTCATGC |
| ZD’C168’ | TACATAGCTGCAGGCCACTAGTCTGCTTGAATCTAGTGGC |
| ZD’C169’ | TACATAGCTGCAGGCCACTATCCTGGGCTTTAATCTCAGC |
| ZD’C170’ | TACATAGCTGCAGGCCACTATCCTTCGATTGGTAGATGGC |
| ZD’C171’ | TACATAGCTGCAGGCCACTAGCTGTGCATTCCCTTCATAG |
| ZD’C172’ | TACATAGCTGCAGGCCACTAGTGACCACTTCTCCTGATGT |
| ZD’C173’ | TACATAGCTGCAGGCCACTAGTGAGGTTGCAGTCATCTTC |
| ZD’C174’ | TACATAGCTGCAGGCCACTACTGGCTCTGGATCTAGTCTC |
| ZD’C175’ | TACATAGCTGCAGGCCACTAAACAGGCATCCTAAGCATCT |
| ZD’C176’ | TACATAGCTGCAGGCCACTAATCCAGGGTGATATGCTACG |
| ZD’C177’ | TACATAGCTGCAGGCCACTACGCATGGGTGAGCTATATCT |
| ZD’C178’ | TACATAGCTGCAGGCCACTAATCTTCATCACCTGACGAGG |
| ZD’C179’ | TACATAGCTGCAGGCCACTAACTGAGACTGCAATCCATGA |
| ZD’C180’ | TACATAGCTGCAGGCCACTAATATGGGAGGGCTCACTCAT |
| ZD’C181’ | TACATAGCTGCAGGCCACTACATCACATGAGGGTTCAGGA |
| ZD’C182’ | TACATAGCTGCAGGCCACTAGCAGGTCATGTGATGCTATC |
| ZD’C183’ | TACATAGCTGCAGGCCACTATACTTTGCCTCTCTGGACAG |
| ZD’C184’ | TACATAGCTGCAGGCCACTAGCGAAAGCTGGGAATCTATC |
| ZD’C185’ | TACATAGCTGCAGGCCACTACGTAAACGTGAGGGTCCTAT |
| ZD’C186’ | TACATAGCTGCAGGCCACTACGTACATTACCTGCCATTGG |
| ZD’C187’ | TACATAGCTGCAGGCCACTAAACCGCCTCATCAAATAGGT |
| ZD’C188’ | TACATAGCTGCAGGCCACTATGACAAGATTTGCCAACTGG |
| ZD’C189’ | TACATAGCTGCAGGCCACTACGCTCAGATGCTGGAGATAT |
| ZD’C190’ | TACATAGCTGCAGGCCACTACAGCTCCTGCCAGTAGATAG |
| ZD’C191’ | TACATAGCTGCAGGCCACTAGAGGTGACTTAGCTTTCTGC |
| ZD’C192’ | TACATAGCTGCAGGCCACTATAGGGAACTTGGGACACTCT |
| ZD’C193’ | TACATAGCTGCAGGCCACTAATTGTGGAGGGCTATCACTC |
| ZD’C194’ | TACATAGCTGCAGGCCACTAATTTGAATGAACTGCCTGCC |
| ZD’C195’ | TACATAGCTGCAGGCCACTACCGACAGTGGGCTATGATAT |
| ZD’C196’ | TACATAGCTGCAGGCCACTACTACAGAATGGGCTAGTGCT |
| ZD’C197’ | TACATAGCTGCAGGCCACTAGGATATGCTTCCTCCTCTGG |
| ZD’C198’ | TACATAGCTGCAGGCCACTACACTTCCGGCATCTATGTTG |
| ZD’C199’ | TACATAGCTGCAGGCCACTAGACTGCTATGGATGGACTCC |
| ZD’C200’ | TACATAGCTGCAGGCCACTACTGGCTACTGCTGGATTAGT |
| ZD’C201’ | TACATAGCTGCAGGCCACTATCGGGCTCTTTAGATCATGG |
| ZD’C202’ | TACATAGCTGCAGGCCACTAAAGGCGCATCTCCATTATCT |
| ZD’C203’ | TACATAGCTGCAGGCCACTACACTGACATGATGAAGGTGC |
| ZD’C204’ | TACATAGCTGCAGGCCACTAAATATCCGGTCATGCACCAT |
| ZD’C205’ | TACATAGCTGCAGGCCACTAACATCAGCTATCCCGGAATG |
| ZD’C206’ | TACATAGCTGCAGGCCACTAGTCTATGTTCCATGATGCGG |
| ZD’C207’ | TACATAGCTGCAGGCCACTATCCTATCATTGGTGAGGTGC |
| ZD’C208’ | TACATAGCTGCAGGCCACTATTGTGCAATTTGGGCTAGAC |
| ZD’C209’ | TACATAGCTGCAGGCCACTAGGCAGTCTTCGGTATGATCT |
| ZD’C210’ | TACATAGCTGCAGGCCACTAAATTCGATGAAGGGACAGCT |
| ZD’C211’ | TACATAGCTGCAGGCCACTACCCTATCCTGCGAGTAGATG |
| ZD’C212’ | TACATAGCTGCAGGCCACTACCCTCCTATGAGCTGATGAG |
| ZD’C213’ | TACATAGCTGCAGGCCACTACCCTGAAATGAGTGGTCTCT |
| ZD’C214’ | TACATAGCTGCAGGCCACTACCGAGGCCGTACTCATATAG |
| ZD’C215’ | TACATAGCTGCAGGCCACTACCGATAATGGCTCATCCTGA |
| ZD’C216’ | TACATAGCTGCAGGCCACTACCGCCTTAACACTATGGGAT |
| ZD’C217’ | TACATAGCTGCAGGCCACTACTTCCGGGCATGTGAGATAT |
| ZD’C218’ | TACATAGCTGCAGGCCACTATGGATCACTTTGTCCTGAGG |
| ZD’C219’ | TACATAGCTGCAGGCCACTAGGCACTGATGTTCACTGATC |
| ZD’C220’ | TACATAGCTGCAGGCCACTACGATGTGTGAGGCAATATCC |
| ZD’C221’ | TACATAGCTGCAGGCCACTACGTACCTGTAGGATGCTCTT |
| ZD’C222’ | TACATAGCTGCAGGCCACTAGAGTTCGACCCTCTTGTCAT |
| ZD’C223’ | TACATAGCTGCAGGCCACTACTCTCTGTACGGTCCTGATG |
| ZD’C224’ | TACATAGCTGCAGGCCACTACTCTTACCTCGTATGGCAGT |
| ZD’C225’ | TACATAGCTGCAGGCCACTACTGAGGTTAGATCGGCTCTC |
| ZD’C226’ | TACATAGCTGCAGGCCACTACTGATATAGGTCGTGGCTCC |
| ZD’C227’ | TACATAGCTGCAGGCCACTACTTCGGAACCTGTATGAGCT |
| ZD’C228’ | TACATAGCTGCAGGCCACTACTTCTGGAGGTGTCGTACAA |
| ZD’C229’ | TACATAGCTGCAGGCCACTAGAAGGCCATGTGGACAATAC |
| ZD’C230’ | TACATAGCTGCAGGCCACTAGAAGGTAGTGTACTGCCTCC |
| ZD’C231’ | TACATAGCTGCAGGCCACTAGAATGCCATCTGCTTTGTCA |
| ZD’C232’ | TACATAGCTGCAGGCCACTAGACATGGGCCAGTGTATCTT |
| ZD’C233’ | TACATAGCTGCAGGCCACTACTAATCATGCCTGGGTCGAT |
| ZD’C234’ | TACATAGCTGCAGGCCACTACTACTAATGCTGAGTGGGCT |
| ZD’C235’ | TACATAGCTGCAGGCCACTAGCAGTAGATGGTCTGTTCCT |
| ZD’C236’ | TACATAGCTGCAGGCCACTAGCAGTCTGTGGATAGTCTCC |
| ZD’C237’ | TACATAGCTGCAGGCCACTAGCATGAACTGTAGTGGTCAC |
| ZD’C238’ | TACATAGCTGCAGGCCACTAGCCACTATTCGAGGTAGGTT |
| ZD’C239’ | TACATAGCTGCAGGCCACTAGCTATACCTTGAACCGGGAT |
| ZD’C240’ | TACATAGCTGCAGGCCACTAGCTTCATGTCCTCTGTCAAG |
| ZD’C241’ | TACATAGCTGCAGGCCACTAGCTTCCGGTGTATTAGGTCA |
| ZD’C242’ | TACATAGCTGCAGGCCACTAGCTTGATGGAATGTCTCCTG |
| ZD’C243’ | TACATAGCTGCAGGCCACTAGGCCCATCTTTGTTTGGAAA |
| ZD’C244’ | TACATAGCTGCAGGCCACTAGGCCTTCTCCACGTATGTAT |
| ZD’C245’ | TACATAGCTGCAGGCCACTAGGCGTGAGTGTTATACCTCT |
| ZD’C246’ | TACATAGCTGCAGGCCACTAGGGTCGTTGAAGTTACTCCT |
| ZD’C247’ | TACATAGCTGCAGGCCACTAGGGTGCTGTCTATGTACTCA |
| ZD’C248’ | TACATAGCTGCAGGCCACTATCAAAGGTTCTTCTCTGCCA |
| ZD’C249’ | TACATAGCTGCAGGCCACTATCAGGATATGTTCTCCGTGG |
| ZD’C250’ | TACATAGCTGCAGGCCACTATCAGGCTTGGTATGATGTCC |
| ZD’C251’ | TACATAGCTGCAGGCCACTATCCTGTCTTGGTAGATCGGA |
| ZD’C252’ | TACATAGCTGCAGGCCACTAAGTTAAGCCAGGTTTCCGAA |
| ZD’C253’ | TACATAGCTGCAGGCCACTACCTTAAAGTCCGTGGGCTTA |
| ZD’C254’ | TACATAGCTGCAGGCCACTACGAGGGTTACAAAGGCTTAC |
| ZD’C255’ | TACATAGCTGCAGGCCACTAAAGGTAACTATGAAGGCCCG |
| ZD’C256’ | TACATAGCTGCAGGCCACTACCCACGCTTAACAAATAGGG |
| ZD’C257’ | TACATAGCTGCAGGCCACTACGGGTCCGTGTATAAAGTCT |
| ZD’C258’ | TACATAGCTGCAGGCCACTACTACCAAGTTACGGACCTGT |
| ZD’C259’ | TACATAGCTGCAGGCCACTACTCTACGGTTAGCCGGTATT |
| ZD’C260’ | TACATAGCTGCAGGCCACTACTGGCGGGTTAAGTCCTATT |
| ZD’C261’ | TACATAGCTGCAGGCCACTAGTAACAGGTTCGTCCCTCTT |
| ZD’C262’ | TACATAGCTGCAGGCCACTATCCACCAATTTAACCAGGGT |
| ZD’C263’ | TACATAGCTGCAGGCCACTAAACCGAGCTAGGGAGACTTA |
| ZD’C264’ | TACATAGCTGCAGGCCACTAAATCCCACTCCAGGGTAGTA |
| ZD’C265’ | TACATAGCTGCAGGCCACTAACGGAGGGTCTAAGTCACTA |
| ZD’C266’ | TACATAGCTGCAGGCCACTATGATACCGTTTAGTGGGTCC |
| ZD’C267’ | TACATAGCTGCAGGCCACTATTAGAGTATTTCGGGTGCCC |
| ZD’C268’ | TACATAGCTGCAGGCCACTACCTCCAATTAAAGCGTCTGG |
| ZD’C269’ | TACATAGCTGCAGGCCACTAAAACTGACAGAATCCCGTCT |
| ZD’C270’ | TACATAGCTGCAGGCCACTAAACACCACTCATACGGGATT |
| ZD’C271’ | TACATAGCTGCAGGCCACTAAACAGAATCGACAGTTCCCT |
| ZD’C272’ | TACATAGCTGCAGGCCACTAACAATCTGGAAGGTGGCTTT |
| ZD’C273’ | TACATAGCTGCAGGCCACTAATTTCCCAACAGTCTAGCGT |
| ZD’C274’ | TACATAGCTGCAGGCCACTACGGTAGGCTGGCTAACTAAT |
| ZD’C275’ | TACATAGCTGCAGGCCACTAGTAAGGGCTTCTACACGTCT |
| ZD’C276’ | TACATAGCTGCAGGCCACTATGGGCCACTTTGAAGGTATT |
| ZD’C277’ | TACATAGCTGCAGGCCACTAAATATGCCAAGCCTCCGTAA |
| ZD’C278’ | TACATAGCTGCAGGCCACTAACAGAGACTGACCCGTACTA |
| ZD’C279’ | TACATAGCTGCAGGCCACTAGCGGGTAGTGTATCCTATCC |
| ZD’C280’ | TACATAGCTGCAGGCCACTAGGGTTATGGTGCTCTATCCC |
| ZD’C281’ | TACATAGCTGCAGGCCACTATCTAGTTGGTTGGTAGCACC |
| ZD’C282’ | TACATAGCTGCAGGCCACTATCTGGCTTTAGATTAGGGCC |
| ZD’C283’ | TACATAGCTGCAGGCCACTATCTGGGAATTGGTTCTAGCC |
| ZD’C284’ | TACATAGCTGCAGGCCACTATGATTCAATTTAAGCCCGGC |
| ZD’C285’ | TACATAGCTGCAGGCCACTATGGAGGAGTTTCCGATTCTC |
| ZD’C286’ | TACATAGCTGCAGGCCACTATGGAGTTCTTTGGAGTCACC |
| ZD’C287’ | TACATAGCTGCAGGCCACTATGTACCTATTTCAGTCGGGC |
| ZD’C288’ | TACATAGCTGCAGGCCACTAACCCGGAATCTAGCATACTG |
| ZD’C289’ | TACATAGCTGCAGGCCACTAGAGAGCCCTTATGTACCTCG |
| ZD’C290’ | TACATAGCTGCAGGCCACTAGCAAAGTGTTACCTCCTGTG |
| ZD’C291’ | TACATAGCTGCAGGCCACTACCACCAGTGATCGGGTATTA |
| ZD’C292’ | TACATAGCTGCAGGCCACTACCTCTGGGCATCCGTATTAA |
| ZD’C293’ | TACATAGCTGCAGGCCACTATTTACATGGTTTGAGGGCCA |
| ZD’C294’ | TACATAGCTGCAGGCCACTAAGAGACTATCGGTGAAAGCC |
| ZD’C295’ | TACATAGCTGCAGGCCACTAAGGAAAGTGAGACGCCTATC |
| ZD’C296’ | TACATAGCTGCAGGCCACTATCAGTTCCTTCTTCGACAGG |
| ZD’C297’ | TACATAGCTGCAGGCCACTATCCGAGTCTTTACACAGTGG |
| ZD’C298’ | TACATAGCTGCAGGCCACTATCCTAACGTGTCCCTTGATG |
| ZD’C299’ | TACATAGCTGCAGGCCACTATCTCTCGGTTTACTGACAGG |
| ZD’C300’ | TACATAGCTGCAGGCCACTAAAGCGATCTGACCAATCCTT |
| ZD’C301’ | TACATAGCTGCAGGCCACTAACACCTGAGAGGGAGTACAT |
| ZD’C302’ | TACATAGCTGCAGGCCACTAACAGCAAATGACCCTAGCTT |
| ZD’C303’ | TACATAGCTGCAGGCCACTAACCAGAATGACACGATCCTT |
| ZD’C304’ | TACATAGCTGCAGGCCACTAACGGATATAGCCCGATCTCT |
| ZD’C305’ | TACATAGCTGCAGGCCACTAACTAAACCTGCACAGTCAGT |
| ZD’C306’ | TACATAGCTGCAGGCCACTAATGTGTAACCAGTGCTCCTT |
| ZD’C307’ | TACATAGCTGCAGGCCACTACAACCACCTGACTAGAGTGT |
| ZD’C308’ | TACATAGCTGCAGGCCACTACGCTAGAGTTACAGGAGCTT |
| ZD’C309’ | TACATAGCTGCAGGCCACTACGTTCCCATACCTGATGAGT |
| ZD’C310’ | TACATAGCTGCAGGCCACTACTAGACCATTAGGCCAGCTT |
| ZD’C311’ | TACATAGCTGCAGGCCACTAGCAGTAGCTTCCAGTACCTT |
| ZD’C312’ | TACATAGCTGCAGGCCACTAGCATACAGTTCCCGGACTAT |
| ZD’C313’ | TACATAGCTGCAGGCCACTAACAGTCACTAGGCCCTGATA |
| ZD’C314’ | TACATAGCTGCAGGCCACTACTCGGTTAGACTGATGGTCA |
| ZD’C315’ | TACATAGCTGCAGGCCACTACTGACCCTTAGTGCCATGTA |
| ZD’C316’ | TACATAGCTGCAGGCCACTAGCAGGTGGTCTGAACTCTTA |
| ZD’C317’ | TACATAGCTGCAGGCCACTATATTTGGGTGTCTGGCACAA |
| ZD’C318’ | TACATAGCTGCAGGCCACTATGAGGCTTTATCTCCTGGGA |
| ZD’C319’ | TACATAGCTGCAGGCCACTATGGCACTTTATTGGGTGACA |
| ZD’C320’ | TACATAGCTGCAGGCCACTATTGGACACTTTGGATGGTCA |
| ZD’C321’ | TACATAGCTGCAGGCCACTACAGGTCGTGAGGCTACTATC |
| ZD’C322’ | TACATAGCTGCAGGCCACTAGCTAGGATTCCCTGACTGTC |
| ZD’C323’ | TACATAGCTGCAGGCCACTAGGAAGCCATTCATTTGTTGC |
| ZD’C324’ | TACATAGCTGCAGGCCACTAGGGATGTCTGGTCATACCTC |
| ZD’C325’ | TACATAGCTGCAGGCCACTAGGTTAATGTCTGTGATCGCC |
| ZD’C326’ | TACATAGCTGCAGGCCACTATCTCTGCATTGGTCTCAAGG |
| ZD’C327’ | TACATAGCTGCAGGCCACTATCTCTGTATTGATGGGAGCC |
| ZD’C328’ | TACATAGCTGCAGGCCACTAGAACACTCTGTGAGGCTGTA |
| ZD’C329’ | TACATAGCTGCAGGCCACTATATGTCTTCTATGCGAGGGC |
| ZD’C330’ | TACATAGCTGCAGGCCACTAGACAAGTCTGGTGAGCAATC |
| ZD’C331’ | TACATAGCTGCAGGCCACTAGGCTTGAATGGCATGATCTC |
| ZD’C332’ | TACATAGCTGCAGGCCACTATCAATGGTTGGCATGGATTC |
| ZD’C333’ | TACATAGCTGCAGGCCACTAGATTAGACTGGTGCTCTGCT |
| ZD’C334’ | TACATAGCTGCAGGCCACTAGAATGCAGTCTATGGCAGTC |
| ZD’C335’ | TACATAGCTGCAGGCCACTAGGTACACCTGTCTCTGTCTG |
| ZD’C336’ | TACATAGCTGCAGGCCACTAACCTGATGCACAGAGCTATC |
| ZD’C337’ | TACATAGCTGCAGGCCACTACCTGATGCTACCTCTGAGTG |
| ZD’C338’ | TACATAGCTGCAGGCCACTAGGTGAATCTTCCTGATGCTG |
| ZD’C339’ | TACATAGCTGCAGGCCACTATCAGCACATTGGTACACTGT |
| ZD’C340’ | TACATAGCTGCAGGCCACTAGCACTCCCTTATGCAGTAGT |
| ZD’C341’ | TACATAGCTGCAGGCCACTACTCCATAATGCTGATGCAGG |
| ZD’C342’ | TACATAGCTGCAGGCCACTACTCCATCTTAGCAGAGCTGT |
| ZD’C343’ | TACATAGCTGCAGGCCACTATGAGGCATTGTGAGAATCCT |
| ZD’C344’ | TACATAGCTGCAGGCCACTAACTATGAATCCAGTGCAGCT |
| ZD’C345’ | TACATAGCTGCAGGCCACTAGCTCAACCTTCATGCAGATT |
| ZD’C346’ | TACATAGCTGCAGGCCACTAGCTCAGACTTCCTACGATGT |
| ZD’C347’ | TACATAGCTGCAGGCCACTATCCATCTCTTCTGTGCAAGG |
| ZD’C348’ | TACATAGCTGCAGGCCACTATCCCAGCTTGTCAGTAGTTG |
| ZD’C349’ | TACATAGCTGCAGGCCACTACACACTCCTGATGAGAGCTT |
| ZD’C350’ | TACATAGCTGCAGGCCACTACACAGAGCTGAGTCGGATAT |
| ZD’C351’ | TACATAGCTGCAGGCCACTATAGGGCTGTTGGCACTTATC |
| ZD’C352’ | TACATAGCTGCAGGCCACTATCCTTGCATTGGTAGTCAGG |
| ZD’C353’ | TACATAGCTGCAGGCCACTACATGCTGGTCCCTACTAGAG |
| ZD’C354’ | TACATAGCTGCAGGCCACTACTGCTCTCTCTCTGATGGAG |
| ZD’C355’ | TACATAGCTGCAGGCCACTAGCATGTCGTGGGATACTCTA |
| ZD’C356’ | TACATAGCTGCAGGCCACTAGCTGTCCGTGGTGATATACT |
| ZD’C357’ | TACATAGCTGCAGGCCACTATAGGTGTTGATTAGCTGCCA |
| ZD’C358’ | TACATAGCTGCAGGCCACTATGCTAGGATTTCATGGGTCC |
| ZD’C359’ | TACATAGCTGCAGGCCACTACATGCACTTAACCCAGATGG |
| ZD’C360’ | TACATAGCTGCAGGCCACTACTGCGCCCTTATATCAATGG |
| ZD’C361’ | TACATAGCTGCAGGCCACTACGGCTCAATGGCTATAACTG |
| ZD’C362’ | TACATAGCTGCAGGCCACTACTGTGACTGACTGTAGCTCT |
| ZD’C363’ | TACATAGCTGCAGGCCACTACTGTGAGCTGCGGTATCTAT |
| ZD’C364’ | TACATAGCTGCAGGCCACTAGGAGCGTGTTCAGACTATCT |
| ZD’C365’ | TACATAGCTGCAGGCCACTACACGTATGGTTTAGACGGTT |
| ZD’C366’ | TACATAGCTGCAGGCCACTACGGAGACATTGTTGTAGCTT |
| ZD’C367’ | TACATAGCTGCAGGCCACTAGTATAACCCTTTGGACACGT |
| ZD’C368’ | TACATAGCTGCAGGCCACTAGTTAATCCCTGTTACCAGCT |
| ZD’C369’ | TACATAGCTGCAGGCCACTAGCTATTAGACTGGCTTATCG |
| ZD’C370’ | TACATAGCTGCAGGCCACTAGGCAGCACTATTACAAAGTC |
| ZD’C371’ | TACATAGCTGCAGGCCACTAGGCTCCTACTAATTGCTATC |
| ZD’C372’ | TACATAGCTGCAGGCCACTACTACGGCTAGGTATATGAGC |
| ZD’C373’ | TACATAGCTGCAGGCCACTACATGGCTATGAGTGAAGCTA |
| ZD’C374’ | TACATAGCTGCAGGCCACTAGCTGGGACTACTAGGAAATA |
| ZD’C375’ | TACATAGCTGCAGGCCACTAGGACGCACGTTTAGATTGTT |
| ZD’C376’ | TACATAGCTGCAGGCCACTAGGTGAAGCCTTTAGAATCGT |
| ZD’C377’ | TACATAGCTGCAGGCCACTACCGTAAAGATTTGTGAGGCT |
| ZD’C378’ | TACATAGCTGCAGGCCACTAGGCATGATTCTTAGACCTTG |
| ZD’C379’ | TACATAGCTGCAGGCCACTAGGCGACTCTATTCTGGTATG |
| ZD’C380’ | TACATAGCTGCAGGCCACTACCGTATTCCTCTGTAGATCG |
| ZD’C381’ | TACATAGCTGCAGGCCACTACCTAAGTCGATTGAAGGAAG |
| ZD’C382’ | TACATAGCTGCAGGCCACTATTGACGCAGATTAGCTCATC |
| ZD’C383’ | TACATAGCTGCAGGCCACTATTGAATATACGTCCTCACGC |
| ZD’C384’ | TACATAGCTGCAGGCCACTATGAGCACACATTAGGCTACC |
| ZE’D1’ | CGCGACCTGTACAGAATTACCCAACTTACCAAGCGTGATT |
| ZE’D2’ | CGCGACCTGTACAGAATTACTGCCTGCATTTCAGTACATG |
| ZE’D3’ | CGCGACCTGTACAGAATTACGTCGTGCATTCTATCATCGG |
| ZE’D4’ | CGCGACCTGTACAGAATTACTATTGTTTAGTCAACGCGGC |
| ZE’D5’ | CGCGACCTGTACAGAATTACCCATCGAGTATCGTCTACGT |
| ZE’D6’ | CGCGACCTGTACAGAATTACCCATTTGAACTAACTGGCGT |
| ZE’D7’ | CGCGACCTGTACAGAATTACCCCGCTGATCTACGTGTATA |
| ZE’D8’ | CGCGACCTGTACAGAATTACCCGGTAGATCGCTATATCGT |
| ZE’D9’ | CGCGACCTGTACAGAATTACCCGTATGTAAATCGTGCTCC |
| ZE’D10’ | CGCGACCTGTACAGAATTACCCGTCAGTTAAACGACTCTG |
| ZE’D11’ | CGCGACCTGTACAGAATTACTCGGTGCATTTAGCAATCTG |
| ZE’D12’ | CGCGACCTGTACAGAATTACCGACACGTCTCTACGATGTA |
| ZE’D13’ | CGCGACCTGTACAGAATTACCGACGTTAAGAAGGTGCTAC |
| ZE’D14’ | CGCGACCTGTACAGAATTACCGACTTCGCCACAATAGTTT |
| ZE’D15’ | CGCGACCTGTACAGAATTACCGAGTATGGCGGTAACTACA |
| ZE’D16’ | CGCGACCTGTACAGAATTACCGAGTTCAAAGCATGTCCAT |
| ZE’D17’ | CGCGACCTGTACAGAATTACCGGAATTAACACTGCTTCGA |
| ZE’D18’ | CGCGACCTGTACAGAATTACGACGACCATGCGTATTCTTT |
| ZE’D19’ | CGCGACCTGTACAGAATTACGACGCTGTTAAAGCTCTGTT |
| ZE’D20’ | CGCGACCTGTACAGAATTACTGTAAATCTTGTGATGGCGC |
| ZE’D21’ | CGCGACCTGTACAGAATTACTCACACATTGTCACAGATGC |
| ZE’D22’ | CGCGACCTGTACAGAATTACTGAATTATTGTCACTGCGGC |
| ZE’D23’ | CGCGACCTGTACAGAATTACATTCCGAGAACTCTGCATCA |
| ZE’D24’ | CGCGACCTGTACAGAATTACCTGCTTCACAATATCGCTGT |
| ZE’D25’ | CGCGACCTGTACAGAATTACCTTACACTGCGTTCAGACTG |
| ZE’D26’ | CGCGACCTGTACAGAATTACGAAAGCTATGTGCTCTCGTC |
| ZE’D27’ | CGCGACCTGTACAGAATTACGAAATACGTGTTACCCGGTC |
| ZE’D28’ | CGCGACCTGTACAGAATTACGAAATCGTTAGTCAGCTCGG |
| ZE’D29’ | CGCGACCTGTACAGAATTACCGTGAGTTAAATCGAGGCTC |
| ZE’D30’ | CGCGACCTGTACAGAATTACCGTGATGCTCAATGCTAGTT |
| ZE’D31’ | CGCGACCTGTACAGAATTACCGTGCATGTAATGACTGCTT |
| ZE’D32’ | CGCGACCTGTACAGAATTACCGTGTATCTAATGCTCCGTG |
| ZE’D33’ | CGCGACCTGTACAGAATTACCGTTAGATCAGATTACCGCG |
| ZE’D34’ | CGCGACCTGTACAGAATTACGCAAATGTGGCTCTGTACTT |
| ZE’D35’ | CGCGACCTGTACAGAATTACGCAACTCTTCGGAAAGTGTT |
| ZE’D36’ | CGCGACCTGTACAGAATTACGCAATTAGCACGTATTCGGT |
| ZE’D37’ | CGCGACCTGTACAGAATTACGCGAATGATGTTACAGTCGT |
| ZE’D38’ | CGCGACCTGTACAGAATTACGCGCTATCTTCAGTCCTGTA |
| ZE’D39’ | CGCGACCTGTACAGAATTACGGCAAATGTTACTCGTGTGT |
| ZE’D40’ | CGCGACCTGTACAGAATTACGGTAGTACGGTGCTCTTACA |
| ZE’D41’ | CGCGACCTGTACAGAATTACGGTTACGTTCGATTATGGCC |
| ZE’D42’ | CGCGACCTGTACAGAATTACGGTTTCAACACTGTGGTTCA |
| ZE’D43’ | CGCGACCTGTACAGAATTACGTAATCTTGACTCTGTGCGG |
| ZE’D44’ | CGCGACCTGTACAGAATTACGTCTTTCATGTACTGGACGC |
| ZE’D45’ | CGCGACCTGTACAGAATTACGTGCAATGTTGGACAGACTT |
| ZE’D46’ | CGCGACCTGTACAGAATTACGTGTCATGTTATCGTGGACC |
| ZE’D47’ | CGCGACCTGTACAGAATTACGTGTTCGTGAATGTTAGCCA |
| ZE’D48’ | CGCGACCTGTACAGAATTACGTTAAGTCTGTCTTCACGGC |
| ZE’D49’ | CGCGACCTGTACAGAATTACGTTGATAGTAGTTCTGCCGC |
| ZE’D50’ | CGCGACCTGTACAGAATTACTACGTTCTTCTGATAGCGGG |
| ZE’D51’ | CGCGACCTGTACAGAATTACTAGCTTCTTGATCTCGACGG |
| ZE’D52’ | CGCGACCTGTACAGAATTACTAGTTTCAAAGCTCTTGGCG |
| ZE’D53’ | CGCGACCTGTACAGAATTACTATTGATGTTATGCGACGGC |
| ZE’D54’ | CGCGACCTGTACAGAATTACTCAAACTATTGTTGTGGGCG |
| ZE’D55’ | CGCGACCTGTACAGAATTACTCTTGATGTTGTCCGAGGAA |
| ZE’D56’ | CGCGACCTGTACAGAATTACTGAATTTAGAGTTGTCGCCG |
| ZE’D57’ | CGCGACCTGTACAGAATTACTTAAGTTTGTGGTACAGCGC |
| ZE’D58’ | CGCGACCTGTACAGAATTACTCGTTTAATTGTGAACCCGG |
| ZE’D59’ | CGCGACCTGTACAGAATTACTTAAACGATTTCGCTTCGGG |
| ZE’D60’ | CGCGACCTGTACAGAATTACTTAATTGGTTTCACGGACGG |
| ZE’D61’ | CGCGACCTGTACAGAATTACAAATTATAGCAACGGGCTCG |
| ZE’D62’ | CGCGACCTGTACAGAATTACACGACAGGTGATTTGTTTCG |
| ZE’D63’ | CGCGACCTGTACAGAATTACTCGTGAACTTTAATTCGCGG |
| ZE’D64’ | CGCGACCTGTACAGAATTACTTATTAACTTTCGCCCGCAG |
| ZE’D65’ | CGCGACCTGTACAGAATTACTTCAGAAATTGTCCGTTCGG |
| ZE’D66’ | CGCGACCTGTACAGAATTACACGAAGGATGAAGCGTACTT |
| ZE’D67’ | CGCGACCTGTACAGAATTACAGAATTATCAACGCTCGGGT |
| ZE’D68’ | CGCGACCTGTACAGAATTACAGCGTGTAACCGAATCTCTT |
| ZE’D69’ | CGCGACCTGTACAGAATTACAGGCACTTACCACACGTTAT |
| ZE’D70’ | CGCGACCTGTACAGAATTACCTCGCTCCTTACGGTAGATT |
| ZE’D71’ | CGCGACCTGTACAGAATTACGCAAACAATTACCTGTCCGT |
| ZE’D72’ | CGCGACCTGTACAGAATTACGTGGCGTGTTATTATCGACC |
| ZE’D73’ | CGCGACCTGTACAGAATTACGTTAGAGATTCGTTCCGTGC |
| ZE’D74’ | CGCGACCTGTACAGAATTACTAACGAAATTGACACTGGGC |
| ZE’D75’ | CGCGACCTGTACAGAATTACTAACTTTGTACTTGACGCGC |
| ZE’D76’ | CGCGACCTGTACAGAATTACTAATTTGTGTAAGTGGCCGC |
| ZE’D77’ | CGCGACCTGTACAGAATTACTACGTTTGTGTAATAGGCGC |
| ZE’D78’ | CGCGACCTGTACAGAATTACTAGTTTAGTTAATGCGGCGC |
| ZE’D79’ | CGCGACCTGTACAGAATTACTATTTCACAACTGTTCGGGC |
| ZE’D80’ | CGCGACCTGTACAGAATTACTTTATGTCTTGGCGGAAAGC |
| ZE’D81’ | CGCGACCTGTACAGAATTACTTTCGGAATTTGTAGGCAGC |
| ZE’D82’ | CGCGACCTGTACAGAATTACAATGCTAACAATCTACGCCG |
| ZE’D83’ | CGCGACCTGTACAGAATTACAATTGACTGAAAGGGCATCG |
| ZE’D84’ | CGCGACCTGTACAGAATTACCTAATGGCTCGGTATCGTTG |
| ZE’D85’ | CGCGACCTGTACAGAATTACTCAACAGGTTCTTTGGTCGA |
| ZE’D86’ | CGCGACCTGTACAGAATTACTCCTTATCTTGTAACCGCGA |
| ZE’D87’ | CGCGACCTGTACAGAATTACTGAACTTTACTCTTCGGGCA |
| ZE’D88’ | CGCGACCTGTACAGAATTACTAACCGTATTGCATAACGCG |
| ZE’D89’ | CGCGACCTGTACAGAATTACTACCGCGTTGTAGAAATGTG |
| ZE’D90’ | CGCGACCTGTACAGAATTACTACGCAAATTGAATGTCGGG |
| ZE’D91’ | CGCGACCTGTACAGAATTACTACGGCGTTGTAACAGATTG |
| ZE’D92’ | CGCGACCTGTACAGAATTACTGCAAATTGGGAGTTCTTCG |
| ZE’D93’ | CGCGACCTGTACAGAATTACTGCCGTAGTTTCAACGTATG |
| ZE’D94’ | CGCGACCTGTACAGAATTACTTAGTATGTTTCACACGGCG |
| ZE’D95’ | CGCGACCTGTACAGAATTACTTTAAGTCTTTGCAGAGCGG |
| ZE’D96’ | CGCGACCTGTACAGAATTACAACGATCTGACAATACGGCT |
| ZE’D97’ | CGCGACCTGTACAGAATTACAACGTCGCTATCTCCAGAAT |
| ZE’D98’ | CGCGACCTGTACAGAATTACAAGAATATCATCCGAGCGGT |
| ZE’D99’ | CGCGACCTGTACAGAATTACCACCACAATGCTAAAGTCGT |
| ZE’D100’ | CGCGACCTGTACAGAATTACCATGCGTATAAATCGGTCGT |
| ZE’D101’ | CGCGACCTGTACAGAATTACGAATGCGGTTCAACAGTCTT |
| ZE’D102’ | CGCGACCTGTACAGAATTACGAGGTCGCTTCACTACTCTT |
| ZE’D103’ | CGCGACCTGTACAGAATTACCCGTAGCCTGCGTACATATA |
| ZE’D104’ | CGCGACCTGTACAGAATTACCCGTCGTATGAGTGCTAGTA |
| ZE’D105’ | CGCGACCTGTACAGAATTACAGCTATTAGAAACATCGCGC |
| ZE’D106’ | CGCGACCTGTACAGAATTACATAATAAATGAGGCGCTGGC |
| ZE’D107’ | CGCGACCTGTACAGAATTACGTGGGCAGTTCTTATATCGC |
| ZE’D108’ | CGCGACCTGTACAGAATTACGTTAGAAATTATGCGTGCGC |
| ZE’D109’ | CGCGACCTGTACAGAATTACTACATTGCGATAACTTCGGC |
| ZE’D110’ | CGCGACCTGTACAGAATTACTACTGTTCTCTTCGAGGAGC |
| ZE’D111’ | CGCGACCTGTACAGAATTACAATCACGTCATAATCAGCGC |
| ZE’D112’ | CGCGACCTGTACAGAATTACGAAATAGTTAGCGCGATGCT |
| ZE’D113’ | CGCGACCTGTACAGAATTACGATGACGTTCAGTGCAATGT |
| ZE’D114’ | CGCGACCTGTACAGAATTACAATGCACAACTCTCACAGTG |
| ZE’D115’ | CGCGACCTGTACAGAATTACAGCTTACAGCACGTATCCAT |
| ZE’D116’ | CGCGACCTGTACAGAATTACGTTGCGAAGATGTTGACTCA |
| ZE’D117’ | CGCGACCTGTACAGAATTACGTTGCGTCCACTTGCATATA |
| ZE’D118’ | CGCGACCTGTACAGAATTACGAACTGTCGATGATGGTCAC |
| ZE’D119’ | CGCGACCTGTACAGAATTACTGAAGACATTTAGCGTCGTG |
| ZE’D120’ | CGCGACCTGTACAGAATTACAGTACAATGCCATACGCTCT |
| ZE’D121’ | CGCGACCTGTACAGAATTACTCAAGTTATTGCGATGTCGG |
| ZE’D122’ | CGCGACCTGTACAGAATTACTGAAGTAATTGTAGCTGCGC |
| ZE’D123’ | CGCGACCTGTACAGAATTACTGGTGATATTTCAATGGCGC |
| ZE’D124’ | CGCGACCTGTACAGAATTACAGTAGACGTGCATAGCAGTT |
| ZE’D125’ | CGCGACCTGTACAGAATTACTGAAATATGGTTATGCGCCG |
| ZE’D126’ | CGCGACCTGTACAGAATTACTAATGTGCTTGATGCTGAGC |
| ZE’D127’ | CGCGACCTGTACAGAATTACTAATTCGATTGATGCGCTGG |
| ZE’D128’ | CGCGACCTGTACAGAATTACATTTAGCATACATCGTCGCG |
| ZE’D129’ | CGCGACCTGTACAGAATTACTACAGCGGTTGCTAAATGTG |
| ZE’D130’ | CGCGACCTGTACAGAATTACCAAAGCGTGCTCCTACATAG |
| ZE’D131’ | CGCGACCTGTACAGAATTACCAGTCAGCTCTAAGTCGTCA |
| ZE’D132’ | CGCGACCTGTACAGAATTACTGTGAAGTTGTTAGCATCGC |
| ZE’D133’ | CGCGACCTGTACAGAATTACACGCACGATCTACATCTGAA |
| ZE’D134’ | CGCGACCTGTACAGAATTACACGCCAGATGACGATATGAT |
| ZE’D135’ | CGCGACCTGTACAGAATTACTACGCCAATTCGCCATTATG |
| ZE’D136’ | CGCGACCTGTACAGAATTACTGTGATTATTTACGCGCAGG |
| ZE’D137’ | CGCGACCTGTACAGAATTACGTACTACATTCGTGCGATGG |
| ZE’D138’ | CGCGACCTGTACAGAATTACTCATCATTGGTAATTCGGCG |
| ZE’D139’ | CGCGACCTGTACAGAATTACACTCAAATGCACATACCGTG |
| ZE’D140’ | CGCGACCTGTACAGAATTACTGTGTCATTGTGTAACAGCG |
| ZE’D141’ | CGCGACCTGTACAGAATTACGCAGTAAATTCAATCGCGTG |
| ZE’D142’ | CGCGACCTGTACAGAATTACGCAGTACATGGTAGTTCAGC |
| ZE’D143’ | CGCGACCTGTACAGAATTACTAGAATGCTTGCCGTTCATG |
| ZE’D144’ | CGCGACCTGTACAGAATTACTGCAAAGCTGTGATTGTCTC |
| ZE’D145’ | CGCGACCTGTACAGAATTACATCAAAGCTGAGCATACGTG |
| ZE’D146’ | CGCGACCTGTACAGAATTACCAATCTAGTATCAGTGGCGC |
| ZE’D147’ | CGCGACCTGTACAGAATTACCAATCTCATGCGATCAACGT |
| ZE’D148’ | CGCGACCTGTACAGAATTACTGCAAGCATTTATTCTCCGG |
| ZE’D149’ | CGCGACCTGTACAGAATTACTGCAATTATTCTTGAGCGGG |
| ZE’D150’ | CGCGACCTGTACAGAATTACCACAAATATGCAACTCGTGC |
| ZE’D151’ | CGCGACCTGTACAGAATTACCTGAATATGCAGTACGCTGG |
| ZE’D152’ | CGCGACCTGTACAGAATTACGCAGTTCATAGAGTGTGCTC |
| ZE’D153’ | CGCGACCTGTACAGAATTACGTCCGTCATGTGTTAGATGC |
| ZE’D154’ | CGCGACCTGTACAGAATTACATCATAAGTGCGACGAGTGA |
| ZE’D155’ | CGCGACCTGTACAGAATTACCATCTCAGTGCTACGTCATG |
| ZE’D156’ | CGCGACCTGTACAGAATTACTAGCACATTGCGTTAGTGTC |
| ZE’D157’ | CGCGACCTGTACAGAATTACTGCATTGTTAATATCGGGCG |
| ZE’D158’ | CGCGACCTGTACAGAATTACTGCGAACCTTTGACAATCTG |
| ZE’D159’ | CGCGACCTGTACAGAATTACCACATCGAGTCAGTCGTAGT |
| ZE’D160’ | CGCGACCTGTACAGAATTACGTCTGCGCTTCTATTCAAGG |
| ZE’D161’ | CGCGACCTGTACAGAATTACTGCGAGAATTTATATGGCGC |
| ZE’D162’ | CGCGACCTGTACAGAATTACCACATGAGTCGAAGTGATCG |
| ZE’D163’ | CGCGACCTGTACAGAATTACCATGCGTGTCAACTGATCTT |
| ZE’D164’ | CGCGACCTGTACAGAATTACCATGCTATGCACTGATCTCG |
| ZE’D165’ | CGCGACCTGTACAGAATTACAGCAACGTCATCACGATCTA |
| ZE’D166’ | CGCGACCTGTACAGAATTACCGCTACCATGATCTAGTCGT |
| ZE’D167’ | CGCGACCTGTACAGAATTACTTGATGTCTTTGAAGCGAGC |
| ZE’D168’ | CGCGACCTGTACAGAATTACTGCTGGAATTTAGTTGACGC |
| ZE’D169’ | CGCGACCTGTACAGAATTACATGAACATCTATCGCTCGCT |
| ZE’D170’ | CGCGACCTGTACAGAATTACCTCGTCTCTGAGTCTGTCAA |
| ZE’D171’ | CGCGACCTGTACAGAATTACGTCAAAGATTCTCCGCTCTG |
| ZE’D172’ | CGCGACCTGTACAGAATTACTGATGTTATTGTAGCGCAGC |
| ZE’D173’ | CGCGACCTGTACAGAATTACTTAATGATTGTGGCTGACGC |
| ZE’D174’ | CGCGACCTGTACAGAATTACACTGAAGATGCACCGCTATA |
| ZE’D175’ | CGCGACCTGTACAGAATTACTCATGTAATTGGATGCGCTG |
| ZE’D176’ | CGCGACCTGTACAGAATTACCATCAGCATGAGTACGACTG |
| ZE’D177’ | CGCGACCTGTACAGAATTACAACTATATCGCATAAGCGCG |
| ZE’D178’ | CGCGACCTGTACAGAATTACTATAGAAATTGCAGCTCGCG |
| ZE’D179’ | CGCGACCTGTACAGAATTACCACTATACTCGGTGAGCTGT |
| ZE’D180’ | CGCGACCTGTACAGAATTACTCTTCTAGTTGTCGAGCAGG |
| ZE’D181’ | CGCGACCTGTACAGAATTACCGTCAGTCTCCGTGTCATAT |
| ZE’D182’ | CGCGACCTGTACAGAATTACCTGATAGTGCTGTAAGTCGC |
| ZE’D183’ | CGCGACCTGTACAGAATTACTCGGACGCTTTAGATGACTT |
| ZE’D184’ | CGCGACCTGTACAGAATTACTCGAACGTGTCATTGGTACT |
| ZE’D185’ | CGCGACCTGTACAGAATTACTCCACAGCTTTACCGTATGT |
| ZE’D186’ | CGCGACCTGTACAGAATTACTAATCCATTGCACTGATGCG |
| ZE’D187’ | CGCGACCTGTACAGAATTACTAATCGAATTGATGGCAGGC |
| ZE’D188’ | CGCGACCTGTACAGAATTACGCGCTTATGTATAGGTCTGC |
| ZE’D189’ | CGCGACCTGTACAGAATTACCAGGCGTATGCGATAATCTC |
| ZE’D190’ | CGCGACCTGTACAGAATTACGAATGCGATTAGTGCGAACT |
| ZE’D191’ | CGCGACCTGTACAGAATTACCGCCTGTTACAAAGTCTGTT |
| ZE’D192’ | CGCGACCTGTACAGAATTACCGCGTAAATGGCTAGGATAC |
| ZE’D193’ | CGCGACCTGTACAGAATTACCATATCGACGTGGTACAGGA |
| ZE’D194’ | CGCGACCTGTACAGAATTACGTCGGATGTGTGCATATCTC |
| ZE’D195’ | CGCGACCTGTACAGAATTACTTACAACTTGGTTTACGGGC |
| ZE’D196’ | CGCGACCTGTACAGAATTACTAAACAATTCGACGTTCGGG |
| ZE’D197’ | CGCGACCTGTACAGAATTACCAAGGAGATGTAACGACTGC |
| ZE’D198’ | CGCGACCTGTACAGAATTACCATTGAACAAACTGTCACGC |
| ZE’D199’ | CGCGACCTGTACAGAATTACCGAACGAATGCTGACTACTC |
| ZE’D200’ | CGCGACCTGTACAGAATTACTCAACAAATTCTATCGGCGG |
| ZE’D201’ | CGCGACCTGTACAGAATTACGAAGTCCTGTGAGTCGATCT |
| ZE’D202’ | CGCGACCTGTACAGAATTACCTACACTGGTACTCGCTGAT |
| ZE’D203’ | CGCGACCTGTACAGAATTACGTTGCTTTGTAATAACGGCG |
| ZE’D204’ | CGCGACCTGTACAGAATTACGCGTATGTGAGTCGTCTCTA |
| ZE’D205’ | CGCGACCTGTACAGAATTACGCCATACCTGTGTACTCGTA |
| ZE’D206’ | CGCGACCTGTACAGAATTACCATCAACATTAAACGCGCTG |
| ZE’D207’ | CGCGACCTGTACAGAATTACTGCTTTGTTATGAAGGCGAC |
| ZE’D208’ | CGCGACCTGTACAGAATTACTGGAGCTGTTTATGTGCAAC |
| ZE’D209’ | CGCGACCTGTACAGAATTACTCTAAACGTGTCTATTGCGC |
| ZE’D210’ | CGCGACCTGTACAGAATTACGGCAAGATTATGCGTCTCTC |
| ZE’D211’ | CGCGACCTGTACAGAATTACACGATCAATCCTCATAGCGT |
| ZE’D212’ | CGCGACCTGTACAGAATTACGTCTAGTTGCATAGCTGTGC |
| ZE’D213’ | CGCGACCTGTACAGAATTACCTTGATACGACTGTCATGGC |
| ZE’D214’ | CGCGACCTGTACAGAATTACCACCTCCGTGACGTAGTATT |
| ZE’D215’ | CGCGACCTGTACAGAATTACCCGAACCTGCGTAACTATTG |
| ZE’D216’ | CGCGACCTGTACAGAATTACCCGAATGTGAAATCATGGCT |
| ZE’D217’ | CGCGACCTGTACAGAATTACCCGCTCGTCTAGCATATAGT |
| ZE’D218’ | CGCGACCTGTACAGAATTACGACTTCAATCGACTTCGCAT |
| ZE’D219’ | CGCGACCTGTACAGAATTACCGTCGATCTCGTCTCATAGT |
| ZE’D220’ | CGCGACCTGTACAGAATTACCTTCTGATGATCTATGCGCG |
| ZE’D221’ | CGCGACCTGTACAGAATTACCGCAAATCTTACCATCGTGT |
| ZE’D222’ | CGCGACCTGTACAGAATTACCGCACACCTGTTTATTCGTA |
| ZE’D223’ | CGCGACCTGTACAGAATTACCGCACCAATGTCTAACAAGT |
| ZE’D224’ | CGCGACCTGTACAGAATTACCGGTGTCGTAACCTCTCTTA |
| ZE’D225’ | CGCGACCTGTACAGAATTACCGTAAATCTGCCTCTCGATG |
| ZE’D226’ | CGCGACCTGTACAGAATTACCGTACAAATCGTTAAAGCGC |
| ZE’D227’ | CGCGACCTGTACAGAATTACCGTACTTCACCACAGTTGAG |
| ZE’D228’ | CGCGACCTGTACAGAATTACGATATTCGGAGTCGTTCAGC |
| ZE’D229’ | CGCGACCTGTACAGAATTACTCACAGCATGTTGGCTAATG |
| ZE’D230’ | CGCGACCTGTACAGAATTACTGTAGATTTATTCACGGCGC |
| ZE’D231’ | CGCGACCTGTACAGAATTACATTGCCTCAACTCGTCATTG |
| ZE’D232’ | CGCGACCTGTACAGAATTACCTTAGCCGTCGTTATGACAG |
| ZE’D233’ | CGCGACCTGTACAGAATTACCTTGACGGCGGTGTACTATA |
| ZE’D234’ | CGCGACCTGTACAGAATTACCTAAGTAATCTGGTCCGCGA |
| ZE’D235’ | CGCGACCTGTACAGAATTACCTAATCGTGCTTCAAACGCT |
| ZE’D236’ | CGCGACCTGTACAGAATTACGCAGCCGATTTAGTTTCGTA |
| ZE’D237’ | CGCGACCTGTACAGAATTACGCTAGTTGAACGATGTGTCC |
| ZE’D238’ | CGCGACCTGTACAGAATTACGCTATCTGTGGACTTATGCG |
| ZE’D239’ | CGCGACCTGTACAGAATTACGCTATGTGTAAGTCTCGTGC |
| ZE’D240’ | CGCGACCTGTACAGAATTACGCTGTTCCGTAAGTGTGATC |
| ZE’D241’ | CGCGACCTGTACAGAATTACGCTTAACGTGGATTCGAGTC |
| ZE’D242’ | CGCGACCTGTACAGAATTACGCTTAGCGTTATCTCGATGG |
| ZE’D243’ | CGCGACCTGTACAGAATTACGCTTTACTAACTGTCTCGGC |
| ZE’D244’ | CGCGACCTGTACAGAATTACGGCTGGCGTTGAATTTCTAA |
| ZE’D245’ | CGCGACCTGTACAGAATTACGTCAAGACTTATTCGCGTGT |
| ZE’D246’ | CGCGACCTGTACAGAATTACGTCAGCAATGTTGAAGCTCT |
| ZE’D247’ | CGCGACCTGTACAGAATTACGTTTCTAACTCTGTCGGCAG |
| ZE’D248’ | CGCGACCTGTACAGAATTACGTTTGCGAATAATGTGTGCC |
| ZE’D249’ | CGCGACCTGTACAGAATTACTAAACCGGGTGATTGATCGT |
| ZE’D250’ | CGCGACCTGTACAGAATTACTAACGTGTAAGTTGACTGCG |
| ZE’D251’ | CGCGACCTGTACAGAATTACTCAACGTGTAGGTTCGTGTA |
| ZE’D252’ | CGCGACCTGTACAGAATTACTCAATGAATTATGTGGCGGC |
| ZE’D253’ | CGCGACCTGTACAGAATTACTCGAATTGTATGTTAGCCGC |
| ZE’D254’ | CGCGACCTGTACAGAATTACTCGGTTTAGATGATGTGCCA |
| ZE’D255’ | CGCGACCTGTACAGAATTACAATTTAGGAAAGCTACGCGG |
| ZE’D256’ | CGCGACCTGTACAGAATTACACTACGAATCGCGGTTAAGA |
| ZE’D257’ | CGCGACCTGTACAGAATTACACTTAGGCACAGTCGGTTAA |
| ZE’D258’ | CGCGACCTGTACAGAATTACGGTAAACTTCGTCAGCCTTC |
| ZE’D259’ | CGCGACCTGTACAGAATTACTAATTGGGTAAAGTTCGCGC |
| ZE’D260’ | CGCGACCTGTACAGAATTACTCGTAGTATTGGGTTCGACC |
| ZE’D261’ | CGCGACCTGTACAGAATTACTGTTAAAGTTTCGGACTGGC |
| ZE’D262’ | CGCGACCTGTACAGAATTACCCTGATTAAACGTAGTGCGG |
| ZE’D263’ | CGCGACCTGTACAGAATTACCGCCAACTTAATTCTTTGCG |
| ZE’D264’ | CGCGACCTGTACAGAATTACGCGAACCGTTACTTACTCTG |
| ZE’D265’ | CGCGACCTGTACAGAATTACTTTAACAGTTTGGCTCGACG |
| ZE’D266’ | CGCGACCTGTACAGAATTACAACACTACTCCGAAGCAGTT |
| ZE’D267’ | CGCGACCTGTACAGAATTACAACCACGATCAGTAGGTGTT |
| ZE’D268’ | CGCGACCTGTACAGAATTACAATAATCATCCCGGTCACGT |
| ZE’D269’ | CGCGACCTGTACAGAATTACAATATAATGACCGCCTCCGT |
| ZE’D270’ | CGCGACCTGTACAGAATTACACCGCACGTACTCAATAAGT |
| ZE’D271’ | CGCGACCTGTACAGAATTACCCGGTGCGTGAGAATAATTT |
| ZE’D272’ | CGCGACCTGTACAGAATTACCGACGGAATTAAACATGCCT |
| ZE’D273’ | CGCGACCTGTACAGAATTACTCCGCCAGTTTAGAAGAGTT |
| ZE’D274’ | CGCGACCTGTACAGAATTACGACACGGTTATACGAATGGC |
| ZE’D275’ | CGCGACCTGTACAGAATTACGATCGAGTTAAGTTCACGGC |
| ZE’D276’ | CGCGACCTGTACAGAATTACGCTACTAATGGTTACGGTGC |
| ZE’D277’ | CGCGACCTGTACAGAATTACGCTCTAGTTCCCGTTAATGC |
| ZE’D278’ | CGCGACCTGTACAGAATTACTCGGTAAGTTTAATGTGCGC |
| ZE’D279’ | CGCGACCTGTACAGAATTACTGGAAAGATTTATACGCGGC |
| ZE’D280’ | CGCGACCTGTACAGAATTACTGTCGAAATTGTACTTGGGC |
| ZE’D281’ | CGCGACCTGTACAGAATTACTGTGTGAGTTTCGTAAAGCC |
| ZE’D282’ | CGCGACCTGTACAGAATTACTTGAATATGGTTTCGGCGAC |
| ZE’D283’ | CGCGACCTGTACAGAATTACACCGTAAATGAATCACGCTG |
| ZE’D284’ | CGCGACCTGTACAGAATTACACGGAATATCCTACGCTCAG |
| ZE’D285’ | CGCGACCTGTACAGAATTACACTCTACATACGGTGACGTG |
| ZE’D286’ | CGCGACCTGTACAGAATTACATTTCTCGTTACACCGCATG |
| ZE’D287’ | CGCGACCTGTACAGAATTACGCGTTAAGTTCCCTGATCTG |
| ZE’D288’ | CGCGACCTGTACAGAATTACAAATGTCCAACGTCACAGTC |
| ZE’D289’ | CGCGACCTGTACAGAATTACAATGAACTCACGTAATCGCC |
| ZE’D290’ | CGCGACCTGTACAGAATTACAATTAAGGCACTATACGCGC |
| ZE’D291’ | CGCGACCTGTACAGAATTACTAGCAGTTGCGGTTAATTCG |
| ZE’D292’ | CGCGACCTGTACAGAATTACTCAAATAATTGCGTGGTCGG |
| ZE’D293’ | CGCGACCTGTACAGAATTACTCGATCCGTTTCAAATGGTG |
| ZE’D294’ | CGCGACCTGTACAGAATTACTGAACACGTTTGAAGACTGG |
| ZE’D295’ | CGCGACCTGTACAGAATTACTGACGAAATTGGACGTTCTG |
| ZE’D296’ | CGCGACCTGTACAGAATTACACGCTCGTCACCGATAATAT |
| ZE’D297’ | CGCGACCTGTACAGAATTACATAACCAGTGATACGAGCGT |
| ZE’D298’ | CGCGACCTGTACAGAATTACATGTTAGCGAACCTGTAGCT |
| ZE’D299’ | CGCGACCTGTACAGAATTACCGAGCCAGTTAAGAGAGTCT |
| ZE’D300’ | CGCGACCTGTACAGAATTACCGCAAATGTCCCATCGTATT |
| ZE’D301’ | CGCGACCTGTACAGAATTACCGTAGTACCGTCTCTGACAT |
| ZE’D302’ | CGCGACCTGTACAGAATTACCTAAGCCATTACGCGACATT |
| ZE’D303’ | CGCGACCTGTACAGAATTACGATGAACGTGCCTTCGATTT |
| ZE’D304’ | CGCGACCTGTACAGAATTACGCCGCCAATTCAAGATTAGT |
| ZE’D305’ | CGCGACCTGTACAGAATTACGCCGTAGTTGAAGAGCATTT |
| ZE’D306’ | CGCGACCTGTACAGAATTACGCGCTTCTGGAAAGTTATGT |
| ZE’D307’ | CGCGACCTGTACAGAATTACGCGTTAAATGTATGGTGCCT |
| ZE’D308’ | CGCGACCTGTACAGAATTACTATGAACGTGGGTTGCTACT |
| ZE’D309’ | CGCGACCTGTACAGAATTACTCACCCGCTTGTAACTATGT |
| ZE’D310’ | CGCGACCTGTACAGAATTACACACGACTGAATCCAGCTTA |
| ZE’D311’ | CGCGACCTGTACAGAATTACCACTAGACTGCGTACACGTA |
| ZE’D312’ | CGCGACCTGTACAGAATTACCGTATGTAACCTGCTCTCGA |
| ZE’D313’ | CGCGACCTGTACAGAATTACGAAGATGGTCTCACGTCGTA |
| ZE’D314’ | CGCGACCTGTACAGAATTACTCACAATTCGGTCTTCTGGA |
| ZE’D315’ | CGCGACCTGTACAGAATTACTCGACCCATGTTTATCGCTA |
| ZE’D316’ | CGCGACCTGTACAGAATTACTGACGGCTTGTGAAGACTTA |
| ZE’D317’ | CGCGACCTGTACAGAATTACTGTCGATTTATTCGGAGGCA |
| ZE’D318’ | CGCGACCTGTACAGAATTACCACGACATCTTTATTTGCGC |
| ZE’D319’ | CGCGACCTGTACAGAATTACCGCGAGAATGGTAACTCATC |
| ZE’D320’ | CGCGACCTGTACAGAATTACGGATTACCTGTTATCGCTGC |
| ZE’D321’ | CGCGACCTGTACAGAATTACGGCAAGAATTGAAATCGTGC |
| ZE’D322’ | CGCGACCTGTACAGAATTACGTAACGTATGTGTGACGCTC |
| ZE’D323’ | CGCGACCTGTACAGAATTACGTTATGCAGTCTTCTCGCAG |
| ZE’D324’ | CGCGACCTGTACAGAATTACGTTCAGGATTCTGTCGATGC |
| ZE’D325’ | CGCGACCTGTACAGAATTACGCGAGGTCTGTCGTATATCT |
| ZE’D326’ | CGCGACCTGTACAGAATTACATGCGCTACAAGTCTCTTCT |
| ZE’D327’ | CGCGACCTGTACAGAATTACTAAGCATTGCAGGTGATTCG |
| ZE’D328’ | CGCGACCTGTACAGAATTACTCAATCATTCGTTCTCGCAG |
| ZE’D329’ | CGCGACCTGTACAGAATTACTGAATAAATTGCTCGTGGGC |
| ZE’D330’ | CGCGACCTGTACAGAATTACGCAACGCATTCCATACATGA |
| ZE’D331’ | CGCGACCTGTACAGAATTACTGAATGCAGTTCTGTTGAGC |
| ZE’D332’ | CGCGACCTGTACAGAATTACTAAGACAGTGTGTGCTGCTA |
| ZE’D333’ | CGCGACCTGTACAGAATTACTATTTGGATTATGGCAGCGC |
| ZE’D334’ | CGCGACCTGTACAGAATTACTCAAAGGCTTGCTTCTGATG |
| ZE’D335’ | CGCGACCTGTACAGAATTACTGAACAGTTGTATTGCTGCC |
| ZE’D336’ | CGCGACCTGTACAGAATTACCAGCGTAGTGGATACAGTCT |
| ZE’D337’ | CGCGACCTGTACAGAATTACTACAAGAATTGCAGGCTGTG |
| ZE’D338’ | CGCGACCTGTACAGAATTACGCACATACTGTCTGAGCATG |
| ZE’D339’ | CGCGACCTGTACAGAATTACTACATACTTGCAGTGCGTTC |
| ZE’D340’ | CGCGACCTGTACAGAATTACTGAGACATTGTGCAAATCGG |
| ZE’D341’ | CGCGACCTGTACAGAATTACTGTGAATATGTGTGTGCCAC |
| ZE’D342’ | CGCGACCTGTACAGAATTACGCACCGTCTGTCGATCTATA |
| ZE’D343’ | CGCGACCTGTACAGAATTACTCAGATGTTGTAATCGTGCG |
| ZE’D344’ | CGCGACCTGTACAGAATTACTCAGCGTGTTGTAATGATGC |
| ZE’D345’ | CGCGACCTGTACAGAATTACGTAATGAATGTGTGCTCGCT |
| ZE’D346’ | CGCGACCTGTACAGAATTACTACGTGAATTGCGATCCATG |
| ZE’D347’ | CGCGACCTGTACAGAATTACTGATAAGCTGTTAATGCCGC |
| ZE’D348’ | CGCGACCTGTACAGAATTACGCAGATCCTCGTTATGATGC |
| ZE’D349’ | CGCGACCTGTACAGAATTACGTATGCAGTGCTCTTCGATG |
| ZE’D350’ | CGCGACCTGTACAGAATTACGCAGCGCATGTTAATGAATG |
| ZE’D351’ | CGCGACCTGTACAGAATTACGTATGTGTGCTGTGTACCAC |
| ZE’D352’ | CGCGACCTGTACAGAATTACTGATGAATTGTCTTAGCGCG |
| ZE’D353’ | CGCGACCTGTACAGAATTACATATGCGATGAAGTCGAGCT |
| ZE’D354’ | CGCGACCTGTACAGAATTACGCTCGCTATTAGTTCAGCTC |
| ZE’D355’ | CGCGACCTGTACAGAATTACTTATCTGATTTCGCACACGG |
| ZE’D356’ | CGCGACCTGTACAGAATTACCTCTGTCAGCTCGCTAGTTA |
| ZE’D357’ | CGCGACCTGTACAGAATTACGCTCTACATGCGTGTTCTAG |
| ZE’D358’ | CGCGACCTGTACAGAATTACTAGAGACTTCTTCGGCTGTG |
| ZE’D359’ | CGCGACCTGTACAGAATTACTGCACGGATTTCACATGATG |
| ZE’D360’ | CGCGACCTGTACAGAATTACCACAATGCTCCCGATGATTT |
| ZE’D361’ | CGCGACCTGTACAGAATTACCTGAGCAATGTCTACACTGC |
| ZE’D362’ | CGCGACCTGTACAGAATTACTAGATTGATTGCGATGCTGC |
| ZE’D363’ | CGCGACCTGTACAGAATTACCACACTGTACGACGATCTTG |
| ZE’D364’ | CGCGACCTGTACAGAATTACTAGCATAGTTGGCACGTCTT |
| ZE’D365’ | CGCGACCTGTACAGAATTACAACCACGCGGGTGTTTGTTT |
| ZE’D366’ | CGCGACCTGTACAGAATTACGGCGAACGCTTTCTGATCTT |
| ZE’D367’ | CGCGACCTGTACAGAATTACACGTGCTGCTTTCGGCATGT |
| ZE’D368’ | CGCGACCTGTACAGAATTACGGAGCGGAATTTCATTTCGT |
| ZE’D369’ | CGCGACCTGTACAGAATTACTTCACATACGTTCGAGCGGG |
| ZE’D370’ | CGCGACCTGTACAGAATTACATAACTCGCGGTCGAAACGG |
| ZE’D371’ | CGCGACCTGTACAGAATTACCTCGGTCCTGCGAATTATGC |
| ZE’D372’ | CGCGACCTGTACAGAATTACAATGAAACCTGCTCGGCGAC |
| ZE’D373’ | CGCGACCTGTACAGAATTACAGATCCCGCGATATACCGGA |
| ZE’D374’ | CGCGACCTGTACAGAATTACTGGCACCTCGCTAAATGGAA |
| ZE’D375’ | CGCGACCTGTACAGAATTACCGGCACCTGTGTTATCACTT |
| ZE’D376’ | CGCGACCTGTACAGAATTACCAGCTCGCGTGTTCTACTGT |
| ZE’D377’ | CGCGACCTGTACAGAATTACCGGGATGACGTTCTAAACAT |
| ZE’D378’ | CGCGACCTGTACAGAATTACGGTGATTGAGTTCCCGTAAT |
| ZE’D379’ | CGCGACCTGTACAGAATTACGACTTGCCATCGTAGAACTG |
| ZE’D380’ | CGCGACCTGTACAGAATTACCCACAATCTTGGACGTAATG |
| ZE’D381’ | CGCGACCTGTACAGAATTACCAGTTCTACGATGGCAAGTC |
| ZE’D382’ | CGCGACCTGTACAGAATTACGTGACATCCAATTAGATCCC |
| ZE’D383’ | CGCGACCTGTACAGAATTACAGGATCAATCGTCAATCACC |
| ZE’D384’ | CGCGACCTGTACAGAATTACATACAGGACCGTCAGAGCGA |
| ZEE001 | GTAATTCTGTACAGGTCGCGATAATCAGCGGGAATCAGGC |
| ZEE002 | GTAATTCTGTACAGGTCGCGGCACTAACTTCACAGAGATC |
| ZEE003 | GTAATTCTGTACAGGTCGCGCCTTGAGCATAATCGAGATC |
| ZEE004 | GTAATTCTGTACAGGTCGCGACATGAGCCATAAGTGTCTC |
| ZEE005 | GTAATTCTGTACAGGTCGCGTATAGTCACGCAGCACGACG |
| ZEE006 | GTAATTCTGTACAGGTCGCGGCCGAGATCACAATATGCCA |
| ZEE007 | GTAATTCTGTACAGGTCGCGTCGCCCACTACAATGAGAGA |
| ZEE008 | GTAATTCTGTACAGGTCGCGCTTCAGAGTAGACTAGATGG |
| ZEE009 | GTAATTCTGTACAGGTCGCGGAGTACGCTCCATAGCTCTG |
| ZEE010 | GTAATTCTGTACAGGTCGCGGCCAGATTGAAACACTGCCA |
| ZF’E001’ | CTAGTACTCGAGATTCTGCCGCCTGATTCCCGCTGATTAT |
| ZF’E002’ | CTAGTACTCGAGATTCTGCCGATCTCTGTGAAGTTAGTGC |
| ZF’E003’ | CTAGTACTCGAGATTCTGCCGATCTCGATTATGCTCAAGG |
| ZF’E004’ | CTAGTACTCGAGATTCTGCCGAGACACTTATGGCTCATGT |
| ZF’E005’ | CTAGTACTCGAGATTCTGCCCGTCGTGCTGCGTGACTATA |
| ZF’E006’ | CTAGTACTCGAGATTCTGCCTGGCATATTGTGATCTCGGC |
| ZF’E007’ | CTAGTACTCGAGATTCTGCCTCTCTCATTGTAGTGGGCGA |
| ZF’E008’ | CTAGTACTCGAGATTCTGCCCCATCTAGTCTACTCTGAAG |
| ZF’E009’ | CTAGTACTCGAGATTCTGCCCAGAGCTATGGAGCGTACTC |
| ZF’E010’ | CTAGTACTCGAGATTCTGCCTGGCAGTGTTTCAATCTGGC |
| ZFZG | GGCAGAATCTCGAGTACTAGAGAGCATGCACATATCTCCC |
| T7’ZG’ | GCGCTTAATACGACTCACTATAGGGAGATATGTGCATGCTCT |
